# A Fluorinated BODIPY-Based Zirconium Metal–Organic Framework for *In Vivo* Enhanced Photodynamic Therapy

**DOI:** 10.1021/jacs.3c12416

**Published:** 2024-01-04

**Authors:** Xu Chen, Bárbara B. Mendes, Yunhui Zhuang, João Conniot, Sergio Mercado Argandona, Francesca Melle, Diana P. Sousa, David Perl, Alexandru Chivu, Hirak K. Patra, William Shepard, João Conde, David Fairen-Jimenez

**Affiliations:** † The Adsorption & Advanced Materials Laboratory (A^2^ML), Department of Chemical Engineering & Biotechnology, University of Cambridge, Philippa Fawcett Drive, Cambridge CB3 0AS, U.K.; ‡ ToxOmics, NOVA Medical School, Faculdade de Ciências Médicas, NMS|FCM, Universidade Nova de Lisboa, Lisboa 2775-405, Portugal; § Synchrotron SOLEIL-UR1, L’Orme des Merisiers, Départementale 128, 91190 Saint-Aubin, France; ∥ Department of Surgical Biotechnology, University College London, London NW3 2PF, U.K.

## Abstract

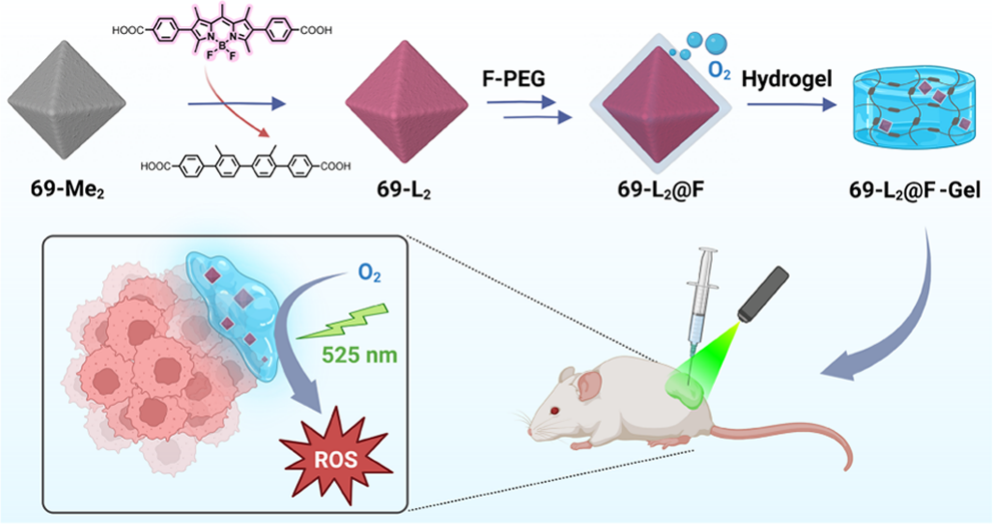

Photodynamic therapy (PDT), an emergent noninvasive cancer treatment, is largely dependent on the presence of efficient photosensitizers (PSs) and a sufficient oxygen supply. However, the therapeutic efficacy of PSs is greatly compromised by poor solubility, aggregation tendency, and oxygen depletion within solid tumors during PDT in hypoxic microenvironments. Despite the potential of PS-based metal–organic frameworks (MOFs), addressing hypoxia remains challenging. Boron dipyrromethene (BODIPY) chromophores, with excellent photostability, have exhibited great potential in PDT and bioimaging. However, their practical application suffers from limited chemical stability under harsh MOF synthesis conditions. Herein, we report the synthesis of the first example of a Zr-based MOF, namely, 69-L_2_, exclusively constructed from the BODIPY-derived ligands via a single-crystal to single-crystal post-synthetic exchange, where a direct solvothermal method is not applicable. To increase the PDT performance in hypoxia, we modify 69-L_2_ with fluorinated phosphate-functionalized methoxy poly(ethylene glycol). The resulting 69-L_2_@F is an oxygen carrier, enabling tumor oxygenation and simultaneously acting as a PS for reactive oxygen species (ROS) generation under LED irradiation. We demonstrate that 69-L_2_@F has an enhanced PDT effect in triple-negative breast cancer MDA-MB-231 cells under both normoxia and hypoxia. Following positive results, we evaluated the *in vivo* activity of 69-L_2_@F with a hydrogel, enabling local therapy in a triple-negative breast cancer mice model and achieving exceptional antitumor efficacy in only 2 days. We envision BODIPY-based Zr-MOFs to provide a solution for hypoxia relief and maximize efficacy during *in vivo* PDT, offering new insights into the design of promising MOF-based PSs for hypoxic tumors.

## Introduction

Photodynamic therapy (PDT) has emerged as an important tool for cancer treatment due to its noninvasive nature, high selectivity for the target tissue, and minimal side effects as compared to conventional medical treatments such as chemotherapy and radiotherapy.^1^ With relative safety, PDT usually acts as adjuvant therapy combined with other treatments in recurrent tumors, in which light-activated photosensitizers (PSs) promote the generation of reactive oxygen species (ROS) to induce cytotoxic effects.^2−4^ However, most reported PDT processes heavily depend on an adequate oxygen supply.^4^ Further, due to rapid cell proliferation and the fact that tumor cells are distant from functional vessels, tumors’ microenvironment is highly hypoxic,^4−7^ thereby compromising the therapeutic efficacy of PDT.^4,8^ Various strategies have been developed to address this issue by increasing the intracellular O_2_ concentration, including hyperbaric oxygen inhalation, reoxygenation using oxygen carriers, or *in situ* O_2_ generation.^4^ Among them, reoxygenation using fluorinated polymers has gained considerable attention due to their chemical inertness and remarkable oxygen solubility, making them ideal artificial oxygen carriers for enhanced PDT application. For example, fluorinated polymers have effectively coupled with PSs, such as porphyrin,^9,10^ IR780,^11^ and boron dipyrromethene (BODIPY)^12^ to relieve tumor hypoxia and improve PDT efficacy.

While metal–organic frameworks (MOFs) have recently emerged as promising PSs for PDT,^13,14^ the existing approaches to alleviate hypoxia using MOFs primarily focus on the intracellular conversion of H_2_O_2_ to O_2_.^15−21^ Alternatively, MOFs containing high valence metal ions, such as Cu (II),^22−24^ Mn (III),^25,26^ and Mn (IV),^27^ have been explored to eliminate intracellular glutathione (GSH) levels.^28^ Despite significant progress achieved in enhanced PDT using MOF by hypoxia relief and GSH depletion,^29,30^ the relatively low intracellular H_2_O_2_ level and high expression of GSH can affect the cell’s reoxygenation and ROS generation capability. Thus, developing a novel strategy to improve the PDT efficiency of MOFs is still in great demand. Herein, we aim to overcome this limitation by building MOF-based PSs with self-oxygen-carrying capabilities. Looking at potential oxygen delivery carriers, highly fluorinated compounds, such as biocompatible perfluorocarbons (PFCs), show high affinity for oxygen molecules and have exhibited great potential.^10,31^ However, due to their poor aqueous solubility, PFCs usually have been formed as short-lived emulsions. On the other hand, hydrophilic polymers such as PEG can promote the aqueous solubility of highly fluorinated compounds.^32,33^ Although PFCs and fluorinated polymers have been widely explored as artificial oxygen carriers, there is very limited research that simultaneously explores the use of MOFs for oxygen delivery and PSs.

As a promising PS, BODIPY has shown great potential in biomedical applications, including fluorescent imaging and sensing,^34^ light harvesting, and PDT.^35,36^ BODIPY exhibits excellent photophysical stability, high molar absorption coefficients, and fluorescence quantum yields, together with a skeleton that is easily functionalized.^37,38^ While previous research has successfully combined BODIPY-based ligands with low-valent metal ions, such as Zn^2+^ and the toxic Cd^2+^,^39−44^ for constructing single-crystalline MOFs, these structures have often exhibited poor chemical stability, potentially limiting their utility in biomedical applications. To date, multiple endeavors have been directed to introduce BODIPY molecules into Zr-based MOFs. However, current strategies are predominantly limited to the post-modification of preexisting ligands or metal ions within MOFs, in which the information regarding the precise positioning and orientation of the immobilized BODIPY moieties is still unclear. To the best of our knowledge, no Zr-based single-crystalline MOFs using BODIPY as a ligand have been reported so far.

In this work, we report an oxygen self-enriched nanoplatform for PDT application through the combination of a fluoropolymer with a BODIPY-based MOF. We first synthesized a BODIPY-based Zr-MOF, namely, 69-L_2_, through post-synthetic ligand exchange (PSE). Owing to the matched ligand lengths, we selected the 2-fold interpenetrated 69-Me_2_ as the parent MOF for PSE. The PSE process occurred in a single-crystal to single-crystal (SC-SC) fashion, and importantly, the successful incorporation of the ditopic BODIPY-derived ligands was confirmed by single-crystal X-ray diffraction (SCXRD). To relieve hypoxia in future PDT *in vivo* experiments, we introduced a perfluorooctyl group into a hydrophilic phosphate-functionalized methoxy poly(ethylene glycol), grafting it onto the external surface of 69-L_2_, leading to the formation of 69-L_2_@F. Upon LED light irradiation, this new system exhibits not only excellent oxygen-carrying capabilities but also improved ROS generation capability compared to a control, 69-L_2_@P, lacking fluorous functionalization. We then combined confocal laser scanning microscopy (CLSM) imaging and transmission electron microscopy (TEM) to examine *in vitro* the internalization of 69-L_2_, 69-L_2_@P, and 69-L_2_@F into triple-negative breast cancer MDA-MB-231 cells. We demonstrated the enhanced PDT efficacy of 69-L_2_@F vs 69-L_2_@P during *in vitro* studies under both normoxic and hypoxic conditions. Finally, *in vivo* studies on triple-negative, luciferase-expressing MDA-MB-231 breast cancer mice models demonstrated significant photodynamic action for tumor growth inhibition of a 69-L_2_@F system combined with a hydrogel for local activity with LED illumination. Overall, our findings provide a feasible way to prepare a BODIPY-based Zr-MOF, whereas the fluorinated nanoplatform demonstrates great potential for hypoxia PDT applications, especially under hypoxic conditions that mimic deep-seated tumors.

## Results and Discussion

### Design Rationale and Characterization of 69-L_2_

**Figure 1:**
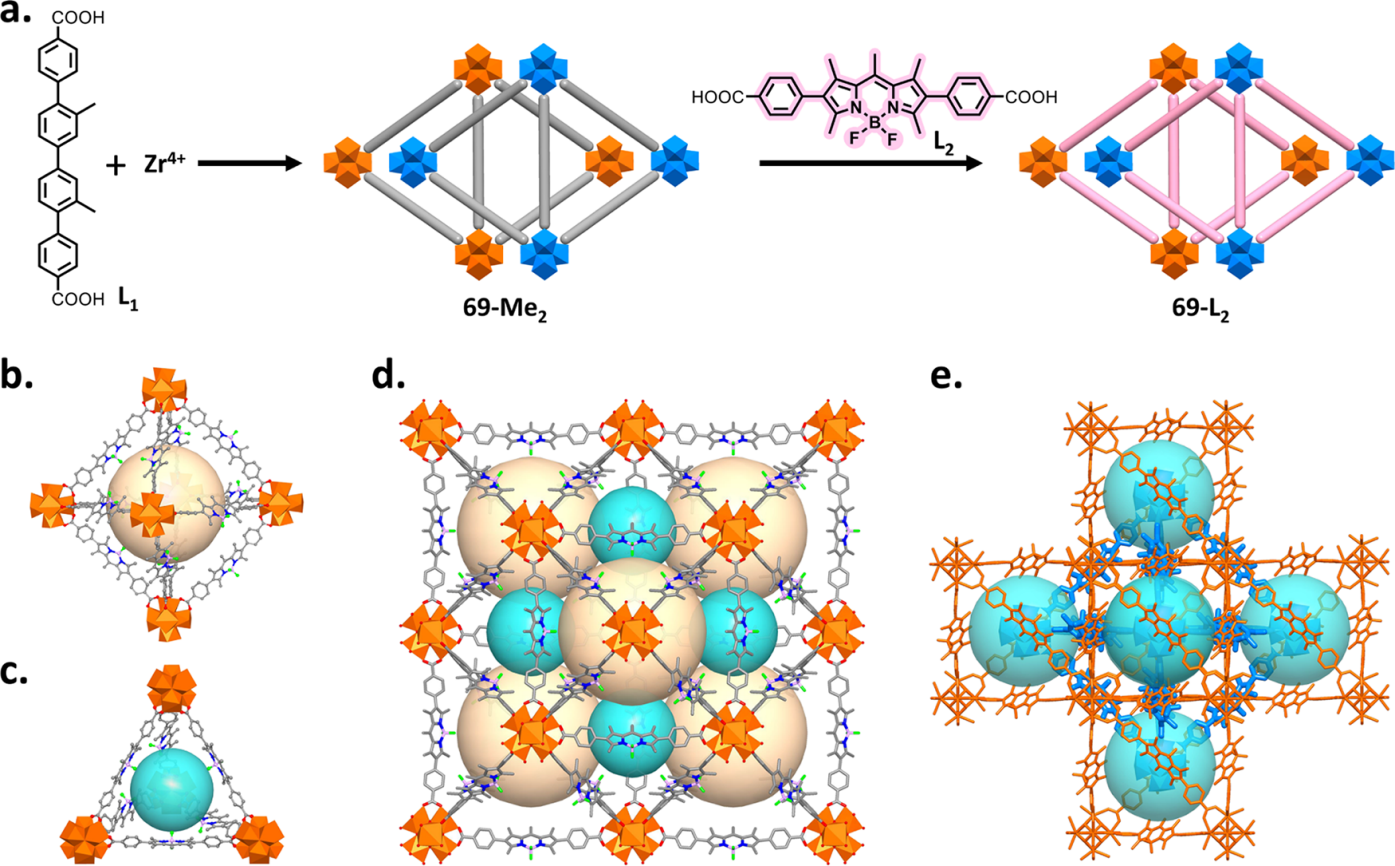
Synthesis and structure of 69-L_2_. (a) Illustration of the synthesis through PSE. (b) Octahedral cavity, (c) Tetrahedral cavity, (d) Their packing mode in the non-interpenetrated fcu network. (e) Structure of the 2-fold interpenetrated network in which the parent and second interpenetrating networks are depicted in brown and blue, respectively. Zr_6_O_8_, brown and blue polyhedra; O, red; b, pink; f, green; C, gray. Hydrogen atoms, guest solvents, and disorder are omitted for clarity.

**Figure 2:**
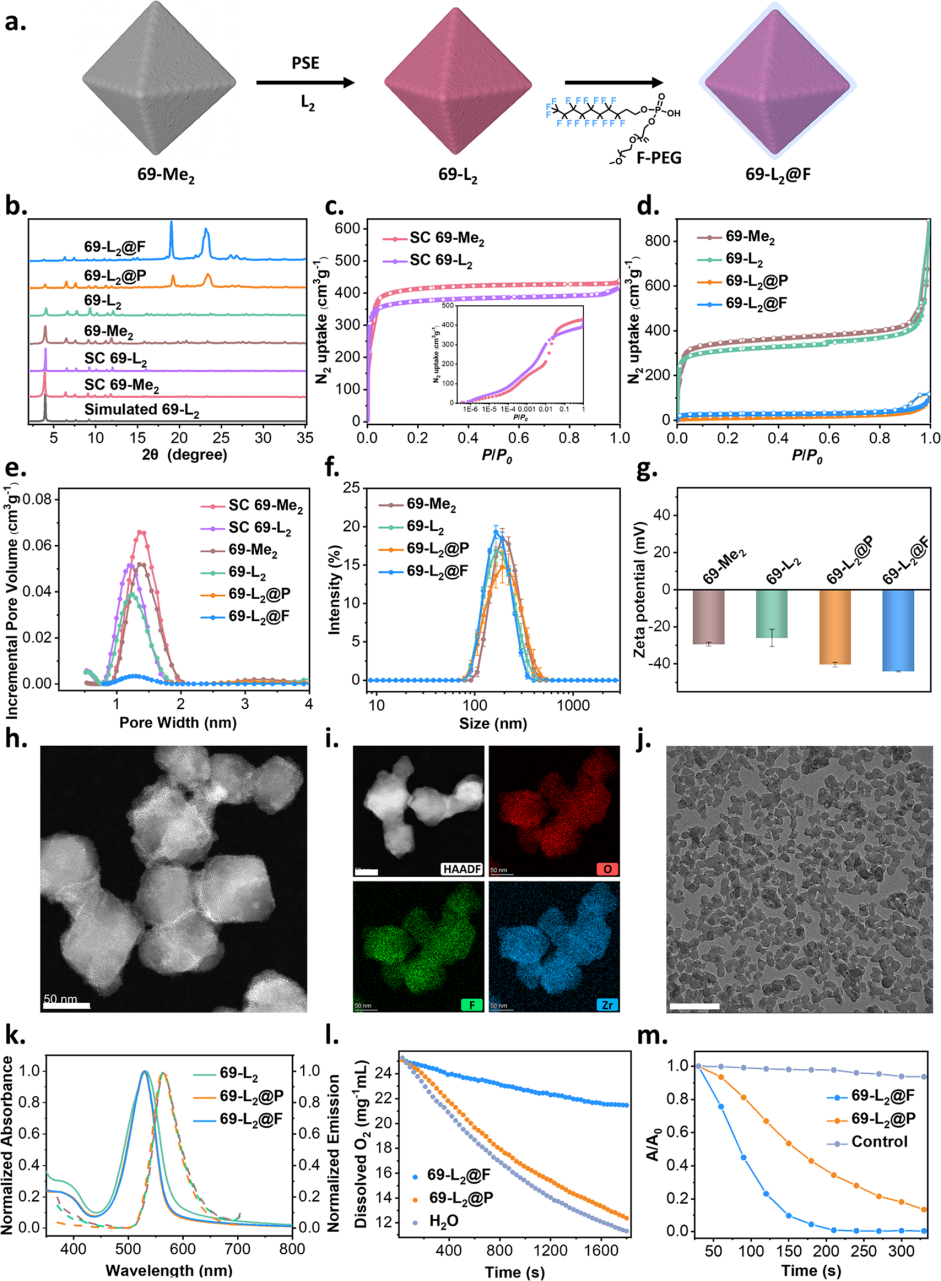
Characterization 69-L_2_, 69-L_2_@P, and 69-L_2_@F. (a) Representation of the PSE and PEGylation. (b) Simulated and experimental PXRD patterns. (c, d) Experimental N_2_ isotherm at 77 K; inset: N_2_ isotherm at 77 K plotted with a logarithmic *x*-axis to highlight the differences in the low-pressure region. (e) PSD obtained with the NLDFT method. (f) Intensity-average diameter and (g) Zeta potential of aqueous suspensions of 69-Me_2_, 69-L_2_, 69-L_2_@P, and 69-L_2_@F (*n* = 3). (h) HAADF-STEM image and (i) EDS mapping of 69-L_2_. Scale bar: 50 nm. (j) TEM image of 69-L_2_@F. Scale bar: 500 nm. (k) UV–vis (solid line) and fluorescence (dashed line, λ_exc_ = 395 nm) spectra of the aqueous solutions of 69-L_2_, 69-L_2_@P, and 69-L_2_@F. (l) Time-dependent changes of dissolved oxygen concentrations in 69-L_2_@F or 69-L_2_@P at the same 69-L_2_ concentrations of 2 mg mL^–1^. (m) DPBF degradation rate curves with 69-L_2_@F and 69-L_2_@P in MeOH.

We first synthesized a carboxylate-functionalized BODIPY ligand (L_2_) via Suzuki coupling, followed by the deprotection of *tert*-butyl esters with trifluoroacetic acid (TFA) in dichloromethane at room temperature (Scheme S2 and Figures S1–S7). Our initial attempts to build a Zr-based MOF with L_2_ were unsuccessful using direct solvothermal methods, likely due to the limited chemical stability of the BODIPY core in the long-term presence of strong acids and high temperatures.^45^ Inspired by previous PSE strategies employing ligands with identical lengths,^46,47^ we hypothesized that L_2_ could replace the ligand (L_1_) of 69-Me_2_ since L_2_ is similar in length to L_1_ (Figures 1a and S44). 69-Me_2_ is a 2-fold interpenetrated *fcu* network with identical ligand–metal node connectivity to that of the UiO-66 to UiO-68 series MOFs (Figure S45).^48,49^ As expected, the single crystals of 69-Me_2_ (denoted as SC 69-Me_2_) changed from colorless to red after incubation in a DMF solution of L_2_ at 70 °C (Figure S20), indicating the successful incorporation of L_2_. ^1^H NMR study on a digested sample shows that L_2_ entirely replaced L_1_ in 69-Me_2_ after 4 days (Figure S21); we denoted this new material as 69-L_2_ and its crystals as SC 69-L_2_. Most importantly, this transformation process takes place in an SC-SC manner, where the SCXRD analysis clearly shows the successful replacement of L_1_ by L_2_. Identical to the parent 69-Me_2_, SCXRD reveals that 69-L_2_, formulated as [Zr_6_O_4_(OH)_4_(L_2_)_6_], is also a 2-fold interpenetrated network, and crystallizes in the cubic space group *Fd3̅m* with a = 38.395 Å. Each hexanuclear [Zr_6_(μ_3_-O)_4_(μ_3_–OH)_4_] cluster is connected to 12 bidentate carboxylate groups from 12 independent L_2_, where the neighboring [Zr_6_(μ_3_-O)_4_(μ_3_–OH)_4_] clusters are linked by one L_2_ ligand, thereby forming an independent 3D framework in *fcu* topology with two types of cavities (Figure 1d): octahedral (2.26 nm, pale yellow, Figure 1b) and tetrahedral (1.31 nm, cyan, Figure 1c). Notably, each L_2_ is disordered on the crystallographic symmetry axis. Two such identical networks are staggered with respect to one another, affording the final 69-L_2_, in which the [Zr_6_(μ_3_-O)_4_(μ_3_–OH)_4_] clusters and three L_2_ ligands within the same trigonal face of the second network partially interpenetrate the tetrahedral and octahedral cavities of the first network, respectively (Figure 1e); 69-L_2_ possesses compromised cavities with diameters of 1.31 nm (Figure S45a). Despite the presence of 2-fold interpenetration, PLATON calculation suggests a 54.5% void volume available for guest inclusion in 69-L_2_.^50^Figure 2b–e shows the powder X-ray diffraction (PXRD) patterns, 77 K N_2_ isotherms, and DFT pore size distribution (PSD) of SC 69-Me_2_ and SC 69-L_2_. Furthermore, beyond our utilization of the meso-methyl BODIPY-based ligand L_2_, we successfully extended this SC-SC transformation methodology to incorporate the comparably bulky meso-phenyl BODIPY-based ligand L_3_, obtaining the single crystal, termed 69-L_3_ (Scheme S3, Figures S8–S14, S44, S45, Tables S1 and S3). To the best of our knowledge, this represents the first example of Zr-based MOFs constructed exclusively from BODIPY-derived ligands through an SC-SC process, since most of the reported BODIPY-containing Zr-based MOFs have been prepared via post-synthetic modification.^51−53^

### Optimization of Nanosized 69-L_2_, 69-L_2_@P, and 69-L_2_@F

To further explore 69-L_2_ for PDT, we next prepared nanosized 69-L_2_ (denoted as 69-L_2_) via PSE on nanosized 69-Me_2_ (denoted as 69-Me_2_). Figure 2a shows the strategy. 69-Me_2_ was synthesized via a solvothermal reaction of L_1_, Zr_6_O_8_ clusters, and acetic acid in DMF at 120 °C (see the Supporting Information for full details). PXRD shows good agreement within 69-L_2_, 69-Me_2_, their single-crystal analogues, and the simulated pattern, indicating phase purity (Figure 2b). Figure 2c,d shows typical Type I isotherms of microporous materials, with a sharp uptake at low relative pressure (*P*/*P*_0_ < 0.01) for SC 69-L_2_ and the related nanoparticles, 69-L_2_, consistent with their parent 69-Me_2_ material. We note that N_2_ uptake at *P*/*P*_0_ = 0.8 decreases from 428 to 393 cm^3^g^–1^ (SC 69-Me_2_ to SC 69-L_2_) and from 407 to 327 m^3^g^–1^ (69-Me_2_ to 69-L_2_ nanoparticles) for micro- and nanosized MOFs in each case after the PSE process with Brunauer–Emmett–Teller (BET) areas—calculated using BETSI^54^ and based on the extended Rouquerol criteria—decreasing from 1639 to 1522 m^2^g^–1^ and from 1388 to 1197 m^2^g^–1^, respectively, (Section S6, Supporting Information). We attribute the lower N_2_ uptakes and BET areas to the slightly increased volume and molecular weight of L_2_ ligands (Figure S44), which could be further confirmed by the decrease in PSD from 1.38 to 1.20 nm (Figure 2e). Additionally, dynamic light scattering (DLS) of an aqueous suspension of 69-L_2_ indicates z-average size and zeta potential of 171 ± 5 nm and −26 ± 5 mV, respectively, identical to the parent 69-Me_2_ (Figure 2f,g). We also note that the particle size obtained through DLS is 2–3 times larger than the TEM and the scan electron microscopy (SEM) sizes, due to the presence of aggregates (Figures 2h and S22 and S23). Specifically, TEM shows that 69-L_2_ nanoparticles exist in the form of aggregation consisting of a few nanoparticles, and each particle exhibits distorted octahedral morphology with a diameter of around 80 nm (Figure 2h). Moreover, high-angle annular dark-field scanning transmission electron microscopy (HAADF-STEM) imaging reveals the existence of highly oriented channels with a lattice spacing of 1.2 nm (Figure S23b), which is slightly smaller than 1.3 nm of the parent 69-Me_2_ (Figure S23a), consistent with the single-crystal structure and the decreased PSD obtained by 77 K N_2_ isotherm (Figures 1e and 2e, and S45a). Further energy-dispersive X-ray spectroscopy (EDS) mapping analysis confirms the uniformly distributed B and F elements within the 69-L_2_ nanoparticles (Figure 2i).

To relieve the hypoxia challenge of solid tumors in future *in vivo* studies, for translational feasibility, and to improve the colloidal stability of the MOF system, we began by considering the PEGylation strategy we recently reported.^55^ In this study, we demonstrated that the introduction of a phosphate-functionalized methoxy polyethylene glycol (P-PEG) onto Zr-based MOFs could simultaneously enhance their colloidal and chemical stability. Given the high oxygen affinity of fluorinated polymers,^32^ we envisioned that integrating fluorinated polymer into P-PEG would combine the merits of fluorinated polymers and P-PEG. Thus, we introduced here a perfluorooctyl segment into the P-PEG, yielding a double-tailed phosphate known as F-PEG (Scheme S4). NMR spectra and size exclusion chromatography with multiangle light scattering (SEC-MALS) confirm the formation and purity of F-PEG (Figures S15–S19). We then performed the PEGylation of 69-L_2_ using F-PEG under the same reported protocol and denoted the resulting composite as 69-L_2_@F (see the Supporting Information for full details). We further prepared 69-L_2_@P by using P-PEG as a control. The amounts of P-PEG and F-PEG in 69-L_2_@P and 69-L_2_@F are 21.7 and 17.7 wt %, respectively, determined by inductively coupled plasma-optical emission spectroscopy (ICP-OES) by measuring the ratio of P to Zr (Table S4), and confirmed using thermogravimetric analysis (TGA) (Figure S29). Consistent with our previous findings,^55^ the crystallinity of 69-L_2_ is preserved after performing the PEGylation using P-PEG and F-PEG, with two new peaks centered at 2θ = 19° and 23°, respectively (Figures 2b and S27). In addition, 77 K N_2_ isotherms show that 69-L_2_@P and 69-L_2_@F adsorb 24 and 37 cm^3^g^–1^ N_2_ at *P*/*P*_0_ = 0.8, respectively, with BET areas decreasing from 1197 to 38 m^2^g^–1^ and 104 m^2^g^–1^, respectively (Section S6, Supporting Information), suggesting the blockage of nitrogen at 77 K toward the internal porosity of 69-L_2_. Fourier transform infrared (FT-IR) spectra indicate the existence of PEG within 69-L_2_@P and 69-L_2_@F (Figure S28). TEM images show the well-preserved morphology after PEGylation with P-PEG and F-PEG, consisting of a few aggregated nanoparticles with diameters of 170 nm, consistent with the parent 69-L_2_ (Figures 2j and S24). Moreover, HAADF-STEM imaging demonstrates the preservation of highly ordered mesopores after PEGylation (Figure S24). The z-average sizes of 69-L_2_@P and 69-L_2_@F are 188 ± 3 and 171 ± 1 nm, respectively, obtained by DLS analysis (Figure 2f). Furthermore, 69-L_2_@P and 69-L_2_@F display more negative zeta potentials of −41 ± 1 and −45 ± 1 mV compared to the parent 69-L_2_ (Figure 2g).

Following the external surface modification of 69-L_2_ with F-PEG, we assessed its impact on the colloidal stability of 69-L_2_. Briefly, we treated bare 69-L_2_ and 69-L_2_@F in water and PBS (pH = 7.4), then monitored hydrodynamic size changes using DLS. As shown in Figure S31, 69-L_2_@F remained stable for up to 8 days, with minimal negligible changes in hydrodynamic size. Although the size of 69-L_2_@F began to increase after 8 days, they still outperformed the bare 69-L_2_, which experienced a rapid increase in size to approximately 400 nm within just 1 day. When treating them in PBS (pH = 7.4), 69-L_2_@F maintained its hydrodynamic size for up to 30 h, after which it underwent rapid aggregation. In contrast, bare 69-L_2_ aggregated dramatically under identical conditions. We also examined the morphological changes for MOF particles using TEM. Both bare 69-L_2_ and 69-L_2_@F retained their morphologies when suspended in water for up to 3 weeks (Figure S25). However, when exposed to PBS (pH = 7.4), bare 69-L_2_ decomposed rapidly after 4 h and degraded completely at 16 h, while the morphologies of 69-L_2_@F remained intact for up to 48 h before particles began to degrade or aggregate (Figure S26). These findings highlight the pronounced improvement in both colloidal stability and chemical stability conferred by F-PEG, consistent with our previous findings.^55^

### Evaluation of ROS Generation

We next studied the photophysical properties and ROS generation capability of 69-L_2_, 69-L_2_@P, and 69-L_2_@F. As shown in Figure S30, solvents have negligible influence on the position of the absorbance spectra of 69-L_2_, which possess identical absorbance bands in DMF, EtOH, and H_2_O, with two typical peaks centered at 530 and 380 nm, originating from the π–π* transitions and the charge-transfer transitions of the BODIPY ligands.^56^ In comparison, solvents appear to have a minor impact on the emission spectra, where the related emission maximum exhibits slight shifts depending on the solvent used (Figure S30). In addition, 69-L_2_, 69-L_2_@P, and 69-L_2_@F have nearly identical absorbance and fluorescence spectra (Figure 2k), suggesting a negligible effect of the PEGylation process on emission. Considering the excellent oxygen affinity of the perfluorooctyl segment,^32^ we then evaluated the oxygen-carrying ability of 69-L_2_@F in water, using water and 69-L_2_@P as controls. The changes in the dissolved oxygen concentration were monitored using a dissolved oxygen meter. Figure 2l shows the time-resolved dissolved O_2_ concentration as a function of the increased time. 69-L_2_@F suspension exhibited a noticeably higher O_2_ concentration compared to those of 69-L_2_@P and pure water, where the presence of PEG component has a negligible effect on O_2_ solubilization, consistent with previous studies.^57^ More specifically, the initial dissolved O_2_ concentration of around 25 mg/mL decreases, after 30 min, to 21.46, 12.36, and 11.31 mg/mL for 69-L_2_@F, 69-L_2_@P, and 69-L_2_, respectively (Figure 2l). These results demonstrate the excellent oxygen-carrying capability of 69-L_2_@F, which can potentially enhance the therapeutic effect of PDT. We further assessed the ROS generation ability of 69-L_2_@F and 69-L_2_@P in air-saturated MeOH using a commercial trapping agent, 1,3-diphenylisobenzofuran (DPBF). Figure S32 shows the degradation rate curves of DPBF, a common ROS quencher, when exposed to 69-L_2_@F and 69-L_2_@P in MeOH under continuous LED light irradiation (525 nm, high-power LED, 3.1 W, SOLIS-525C, ThorLabs), whereas 69-L_2_@F shows enhanced ROS generation capability with an 80% decrease in the relative absorbance at 410 nm after 120 s (Figure 2m). For comparison, 69-L_2_@F takes 270 s to achieve an 80% decrease, whereas the control group exhibits a negligible decline of DPBF absorbance under the same irradiation treatment (Figure S32c). Overall, these results demonstrate the 69-L_2_@F could act as an efficient PS for PDT applications while simultaneously holding the capability of carrying O_2_.

### Impact of Enhanced PDT on Breast Cancer Cells’ Phenotype

**Figure 3:**
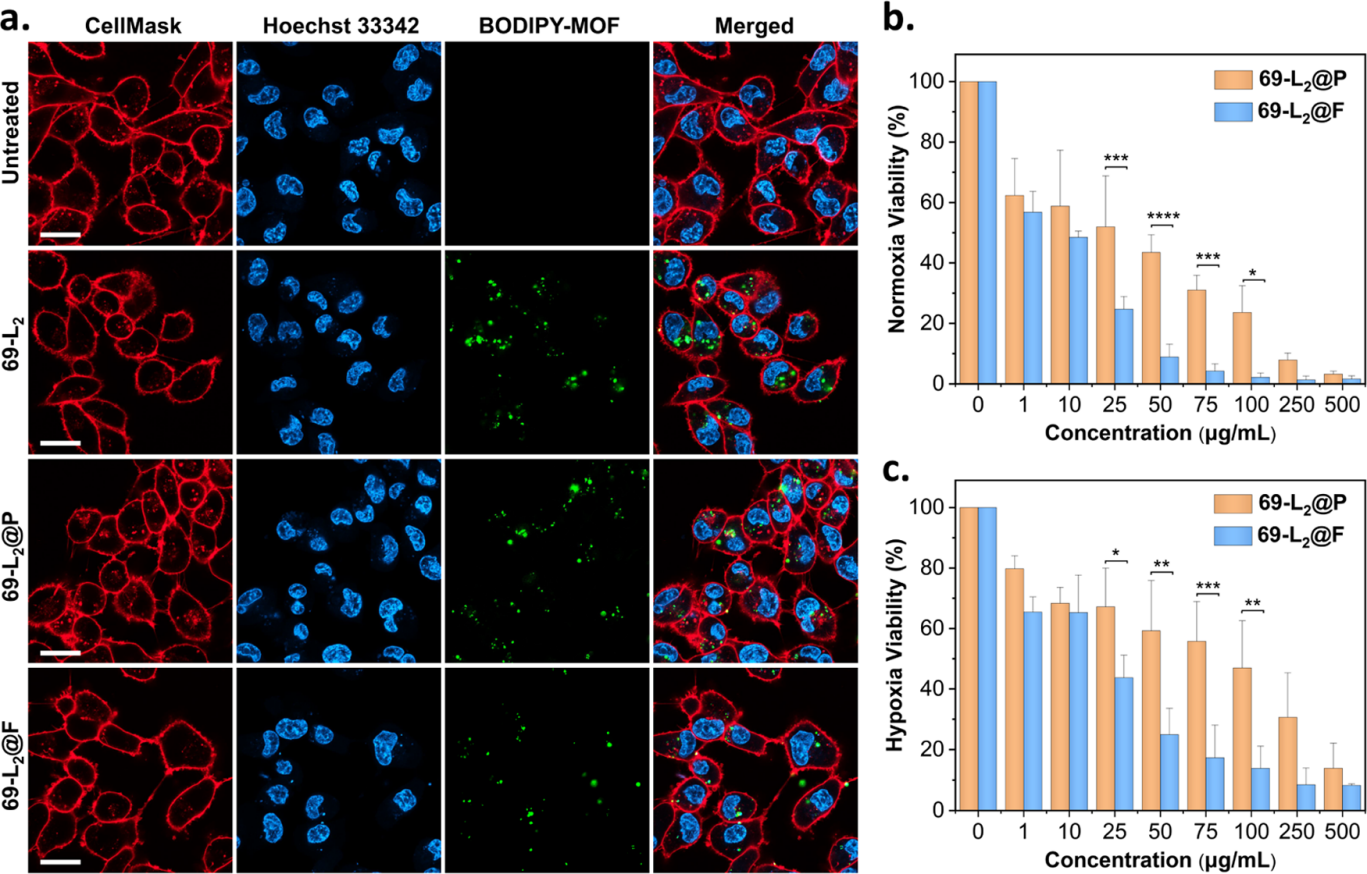
Intracellular uptake and photocytotoxicity of 69-L_2_, 69-L_2_@P, and 69-L_2_@F. (a) CLSM images of MDA-MB-231 cells after incubating with 69-L_2_, 69-L_2_@P, and 69-L_2_@F for 24 h at a concentration of 100 μg/mL; scale bar, 20 μm. MDA-MB-231 cells’ viability was measured using the methyl thiazolyl tetrazolium (MTT) assay. (b) Cells were incubated under normoxic conditions and (c) cells were incubated under hypoxic conditions with 1% O_2_ after LED light irradiation. Concentrations are based on bare 69-L_2_. Data are presented as mean ± SD (*n* = 3; ****p < 0.001, ***p < 0.001, **p < 0.01, *p < 0.05, two-way ANOVA followed by a Sidak’s test for multiple comparisons).

Breast cancer is one of the most commonly diagnosed cancers worldwide.^58^ Triple-negative breast cancer, a subset of breast cancer, is considered the most aggressive and invasive.^59^ Here, we selected triple-negative breast cancer cell MDA-MB-231 and studied the PDT effect of our BODIPY-based MOFs. We first examined the biocompatibility and intracellular uptake of 69-L_2_, 69-L_2_@P, and 69-L_2_@F. As shown in Figure S33, 69-L_2_@P and 69-L_2_@F exhibit negligible cytotoxicity after incubation for 24 h in dark conditions, even at concentrations of 500 μg/mL. In comparison, bare 69-L_2_ shows a reduction of over 30% in viability under the same concentration of 500 μg/mL. Altogether, this is consistent with our previous findings, where the presence of PEG around the nanosized MOFs could significantly improve their biocompatibility due to the limited aggregation of the nanoparticles.^55^ To validate the cellular uptake of our BODIPY-based MOFs, we performed CLSM imaging. Figure 3a shows increased fluorescence signals of the BODIPY core in MDA-MB-231 cells after incubation cells with 69-L_2_, 69-L_2_@P, or 69-L_2_@F for 24 h; Videos S1–S3 and Figure S35 show the 3D z-stack CLSM, where 69-L_2_, 69-L_2_@P, and 69-L_2_@F are internalized and accumulated inside the cells. Figure S34 shows the TEM imaging of the heavy-metal-stained MDA-MB-231 cells where we observe the presence of the cell membrane and the nucleus as well as the internalized 69-L_2_@P and 69-L_2_@F nanoparticles. Taken together, these results suggest excellent biocompatibility and successful internalization of 69-L_2_@P and 69-L_2_@F required for PDT.

Encouraged by these observations and the wide application of BODIPY-based PSs in PDT,^35,36^ we evaluated the PDT efficacy of 69-L_2_@F under both normoxia and hypoxia conditions *in vitro*, using 69-L_2_@P as a control. Figure 3b shows that, upon light irradiation, MDA-MB-231 cells incubated with 69-L_2_@P and 69-L_2_@F exhibit more than 40% reduction in viability at MOF concentrations as low as 10 μg/mL under normoxic conditions. When increasing the concentration to 25–100 μg/mL, 69-L_2_@F shows a significant reduction in cell viability compared to 69-L_2_@P, suggesting its superior *in vitro* PDT efficacy. The difference in cell viability could be attributed to the O_2_ enrichment of fluorous tags around 69-L_2_, which would attract the O_2_ within the media, thus improving the ROS generation and PDT effect. Unlike in normoxia, 69-L_2_@P and 69-L_2_@F show a similar trend in viability but to a lesser extent at low concentrations under hypoxic conditions—with 69-L_2_@F having a consistently higher efficacy than 69-L_2_@P. Cell viabilities under hypoxic conditions are slightly higher than the same concentrations under normoxia (Figure 3c). We attribute this finding to the insufficient O_2_ supply in hypoxia for ROS generation. Notably, at concentrations over 25 μg/mL, 69-L_2_@F shows higher toxicity against MDA-MB-231 cells in hypoxia than those treated with 69-L_2_@P. Again, the excellent oxygen-carrying capability provided by perfluorooctyl-containing 69-L_2_@F could lead to higher concentrations of oxygen and hence higher PDT efficacy against MDA-MB-231 cells *in vitro* when irradiated by LED light, favoring PDT treatment with deep-seated tumors.

**Figure 4:**
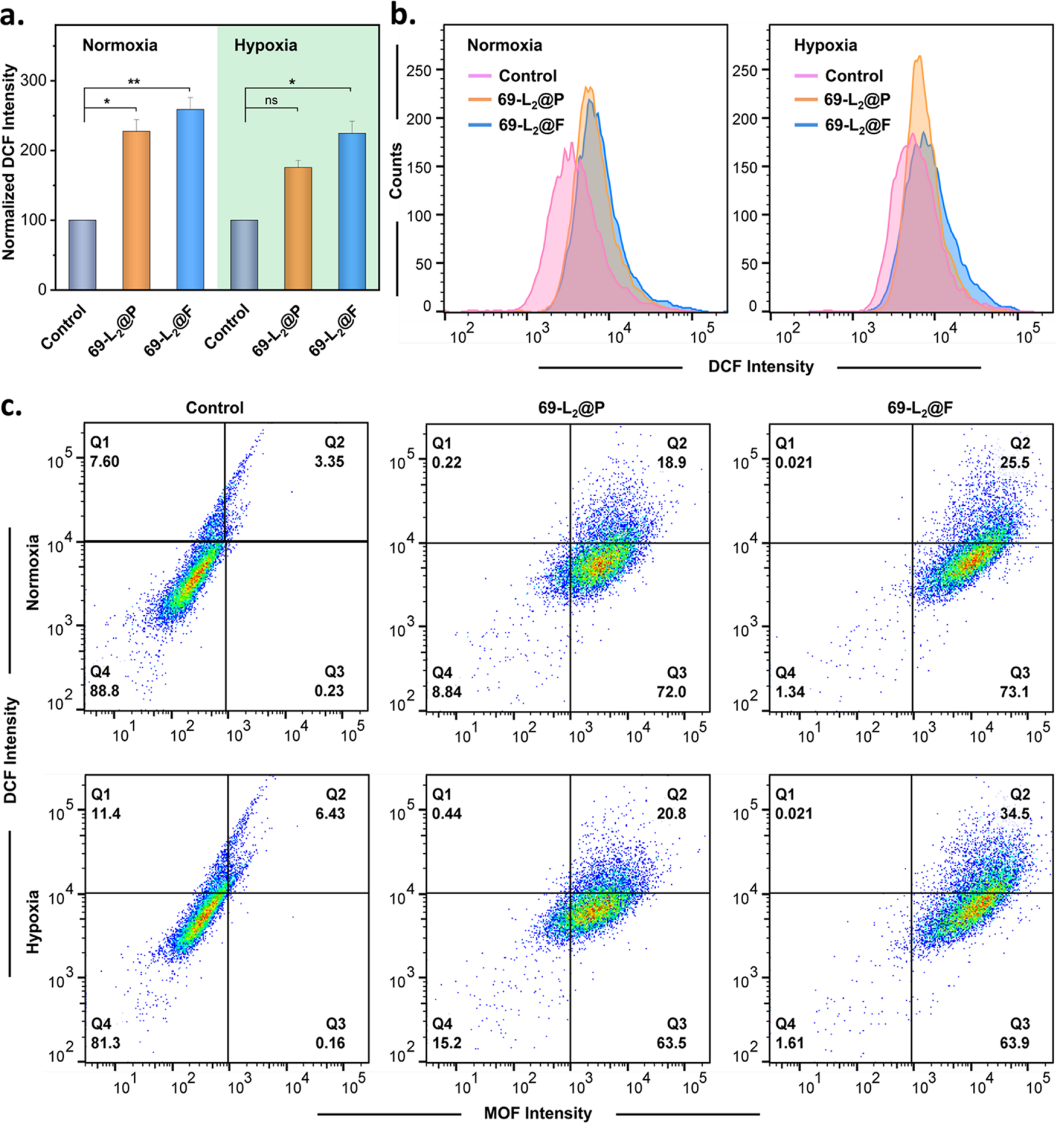
ROS generation of 69-L_2_@P and 69-L_2_@F. MDA-MB-231 cells were treated with 69-L_2_@P or 69-L_2_@F for 24 h at a concentration of 25 μg/mL based on 69-L_2_, followed by green LED light irradiation. ROS generation was measured by the DCF fluorescence intensity using flow cytometry. (a) Normalized DCF fluorescent intensity (*n* = 5; **p < 0.01, *p < 0.05, two-way ANOVA followed by a Sidak’s test for multiple comparisons). (b) Overlapped histograms of DCF intensity for normoxic and hypoxic cells. (c) Flow cytometry analysis of cells with MOF intensity vs DCF intensity.

To assess the intracellular ROS generation capability of 69-L_2_@P and 69-L_2_@F, we selected 2′,7′-dichlorodihydrofluorescein diacetate (DCF-DA) as a probe to detect intracellular ROS levels. Briefly, MDA-MB-231 cells were incubated with 25 and 100 μg/mL of either 69-L_2_@P or 69-L_2_@F for 24 h before being irradiated with an LED light for 10 min. Figure S36 shows how all of the MDA-MB-231 cells treated with 69-L_2_, 69-L_2_@P, or 69-L_2_@F at 25 and 100 μg/mL concentrations exhibit dim-green fluorescence from DCF before light irradiation. After 10 min of LED light treatment, the intracellular fluorescence becomes bright green, demonstrating the successful generation of ROS. Figure 4a shows the intracellular ROS level when treated with 69-L_2_@P and 69-L_2_@F, quantified by the fluorescence intensity of DCF; ROS levels are 3.5- and 4-fold higher than the control group in normoxia. This further suggests that the photocytotoxicity observed in normoxia was due to the significant increase in intracellular ROS generated by the BODYIPY core (Figure 4b), leading to enhanced oxidative toxicity. Figure 4a shows the normalized DCF fluorescent intensity of approximately 175 and 225 for 69-L_2_@P and 69-L_2_@F in the case of hypoxic cells. We observed a ca. 1.8- and 2.3-fold increase for 69-L_2_@P and 69-L_2_@F, respectively, compared to the control but to a lesser extent when compared to normoxia. Again, we note that 69-L_2_@F generated more intracellular ROS than 69-L_2_@P under both normoxia and hypoxia conditions (Figures 4a,b, and S37). Indeed, the difference in ROS generation between 69-L_2_@F and 69-L_2_@P under hypoxia is approximately 12% higher than normoxia, possibly due to a slight increase in O_2_ concentration caused by the oxygen-carrying 69-L_2_@F at the cellular level. Figure 4c shows the DCF intensity as a function of BODIPY-MOFs intensity quantified by flow cytometry; 69-L_2_@P and 69-L_2_@F had similar cellular uptakes under both normoxia and hypoxia compared to the related controls. In addition, we used IncuCyte to perform real-time monitoring of the live MDA-MB-231 cells, which were treated with 69-L_2_, 69-L_2_@P, or 69-L_2_@F (see the Supporting Information for full details). As shown in Figures S38 and S39, the untreated cells proliferated throughout the observation period, regardless of light irradiation. Similar trends were also found in the treated cells without light irradiation (Figure S39 and Video S4). In contrast, MDA-MB-231 cells exhibited significant changes in morphology and movement after light treatment (Figure S39 and Video S5). All of these solidly showcase the promising potential of 69-L_2_@F for PDT against hypoxic tumors in real setups.

### *In Vivo* Therapeutic Efficacy and Biodistribution

**Figure 5:**
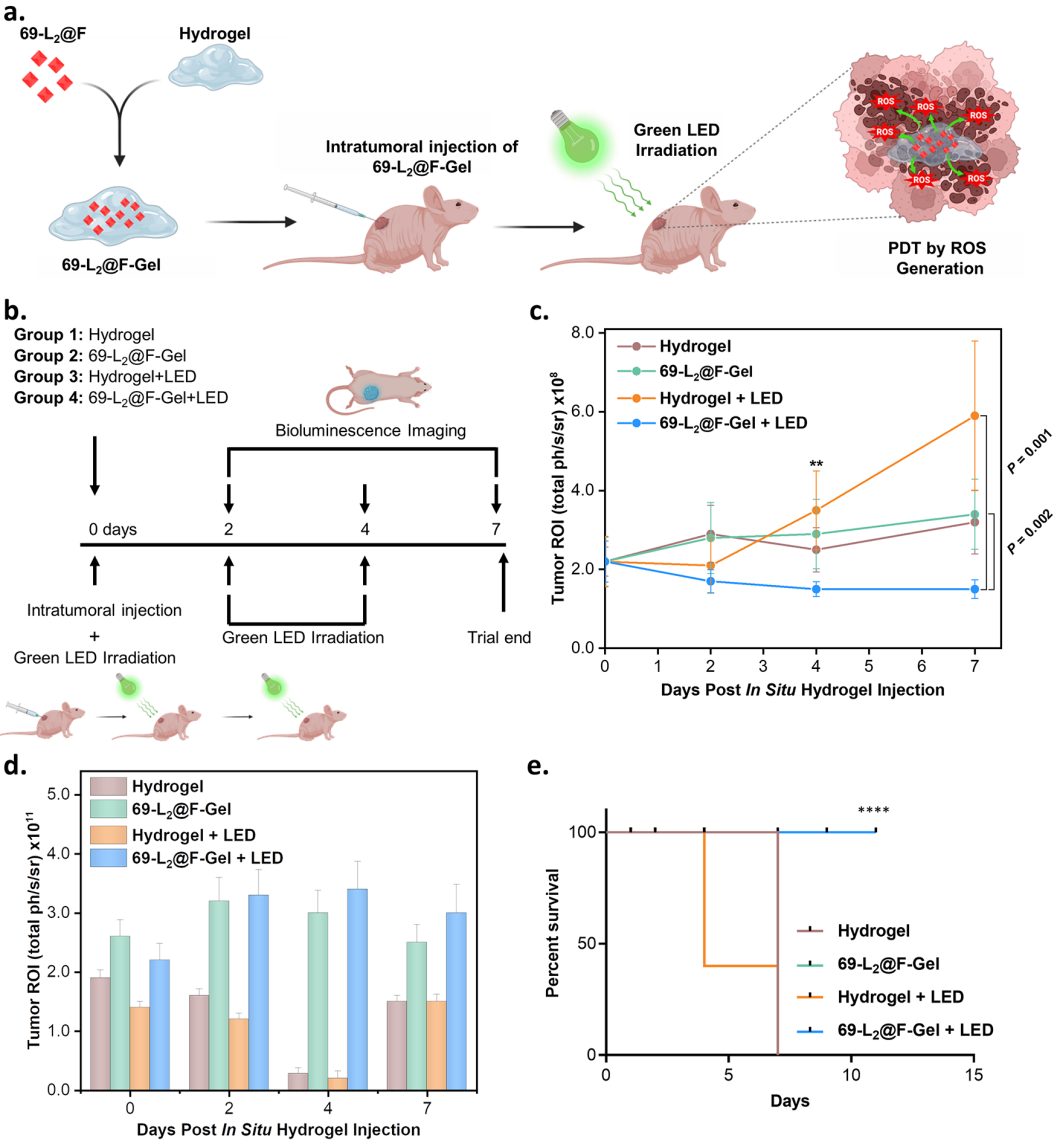
In vivo studies. (a) Schematic showing the preparation of 69-L_2_@F-Gel and their photodynamic therapeutic mechanism. (b) In vivo experimental setup. (c) Tumor size (*n* = 5) following treatment (statistical analysis was performed with multiple *t* tests, **P < 0.01). (d) Fluorescence signal of 69-L_2_@F after intratumoral injections. (e) Kaplan–Meier survival curve after treatment (statistical analysis was performed with Log-rank (Mantel–Cox) test, ****P < 0.0001). Survival cutoff criteria included tumor ulceration or compassionate euthanasia, when the aggregate tumor burden >50% difference between treated groups and controls, or if the tumor impeded eating, urination, defecation, or ambulation.

We proceeded to study the *in vivo* therapeutic efficacy and biodistribution of 69-L_2_@F in a triple-negative luciferase-expressing MDA-MB-231 breast cancer model in mice. Here, we used a localized therapy, developing a hydrogel scaffold to allow the delivery of a higher “effective” local dose of the MOF. This not only enhances MOFs’ therapeutic stability but also minimizes side effects and reduces the clearance of the therapeutic agent by the body’s metabolic and excretory systems. Consequently, the treatment remains at the tumor site for a longer duration, potentially improving its therapeutic effect. Previously, we developed several localized therapies that surpassed the efficacy of systemic approaches. These innovations pave the way for effective neoadjuvant therapy, treating nonresectable tumors, or performing washout procedures post-tumor resection to prevent recurrence.^60−63^ In fact, the systemic administration of the MOFs presented in this study may lead to their nonspecific accumulation in various organs, resulting in unwanted off-target effects. More importantly, their intrinsic photodynamic capacity, crucial to their therapeutic function, might be at risk of compromise due to their widespread distribution in the body. With this in mind, 69-L_2_@F was loaded in a hydrogel as a localized delivery depot, denoted as 69-L_2_@F-Gel, and mice received a single intratumoral injection of the treatment (Figure 5a). SEM imaging shows the well-maintained morphology after forming the 69-L_2_@F-Gel composite (Figure S40); EDS mapping confirms the homogeneous distribution of 69-L_2_@F within the hydrogel (Figure S41). Following intratumoral injection of 69-L_2_@F-Gel, the mice were exposed to a green LED (69-L_2_@F-Gel + LED). Figure 5b depicts the therapeutic scheme, with the green LED exposure repeated on days 2 and 4 following intratumoral injection. Using a live imaging system, we investigated tumor progression inhibition by luciferase expression. Body weight remained unaltered during the experiment (Figure S42), suggesting the biocompatibility of the treatment without associated toxicity or side effects. Figure 5c shows the evolution of tumor size following the treatment; a tumor region of interest (ROI) of 1.5 × 10^8^ and 2.9 × 10^8^ were obtained for the 69-L_2_@F-Gel + LED and the 69-L_2_@F-Gel treatments, respectively. The 69-L_2_@F-Gel + LED treatment significantly reduced the tumor size at day 4 (*n* = 5, *P* = 0.002) compared to the control hydrogel + LED, with more than 80% (*n* = 5, *P* = 0.001) reduction at day 7 after intratumoral injection. The tumor regression shown here is consistent with the hypoxia *in vitro* results (Figure 3c), where MDA-MB-231 cell viability significantly decreased at day 3 of treatment with 69-L_2_@F + LED. In the *in vivo* studies, we tracked 69-L_2_@F fluorescently, showing that 69-L_2_@F remained at the tumor site for 7 days after its injection (Figure 5d). In the past, Conde and co-workers have shown that hydrogels are able to sense and differentially react with the disease microenvironment, potentiating targeted drug release and uptake in certain disease settings. These hydrogels also prove to be incredibly efficient for tumor size reduction (ca. 80–85%) 14 days post-gel implantation.^60,62−64^ Taking one step forward, the cellulose-based hydrogels developed in this study were injected inside the tumor tissue, leading to the *in situ* activation of 69-L_2_@F with LED light, allowing the production of ROS from the inside to the outside of the tumor microenvironment. Overall, the 69-L_2_@F-Gel + LED treatment inhibited tumor growth significantly more than the control groups, resulting in a more potent effect in terms of speed, reducing more than 80% of the tumor in only 7 days. Remarkably, tumor regression occurs after a single injection with no adverse effects in mice. Moreover, the inhibition of tumor growth significantly increased mice survival (*P* < 0.0001) up to day 11 in the 69-L_2_@F-Gel + LED group (Figure 5e), when compared to the other groups, which survived up to day 7, reinforcing the effectiveness of the treatment.

**Figure 6:**
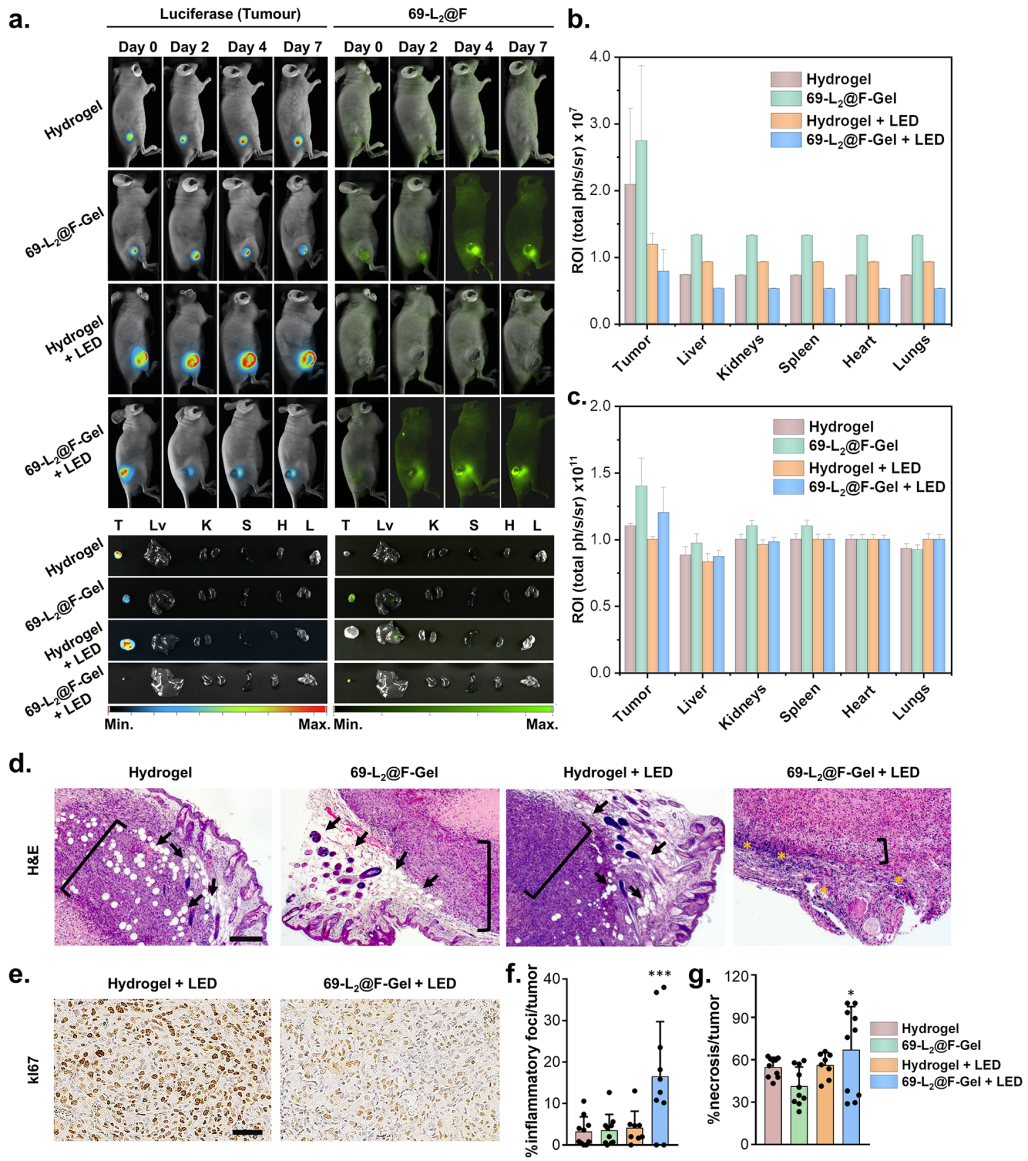
(a) Live imaging of athymic Balb/C female nude mice with triple-negative breast tumor xenograft implanted with hydrogel or 69-L_2_@F-Gel with or without green LED irradiation (*n* = 5 per group). *Ex vivo* images of breast tumors and whole organs (T, tumor; Lv, liver; K, kidneys; S, spleen; H, heart; L, lung) are also presented. (b) Luminescence signal of luciferase-expressing MDA-MB-231 cells in breast tumors and whole organs at day 7 post *in situ* hydrogel injection. (c) Fluorescence signal of 69-L_2_@F in breast tumors and whole organs at day 7 post *in situ* hydrogel injection. (d) Hematoxylin and eosin (H&E) stains of tumors from treated groups with hydrogel or 69-L_2_@F-Gel with or without green LED irradiation. Tumor front, bracket; Adipocytes, arrow; and necrotizing tissue, asterisk. Scale bar: 200 μm. (e) Immunohistochemistry staining of KI-67 (proliferation marker) of tumors from treated groups with hydrogel or 69-L_2_@F-Gel after green LED irradiation, as seen by the brown coloring. Scale bar: 50 μm. (f) Percentage of inflammatory foci/tumor in the four groups tested (statistical analysis was performed with one-way ANOVA test, ***P < 0.001). (g) Percentage of necrosis/tumor in the four groups tested (statistical analysis was performed with one-way ANOVA test, *P < 0.05).

Live imaging of the treated mice supports the inhibition of tumor growth by demonstrating clear tumor regression (Figure 6a,b) from day 0 to day 7 following *in situ* treatment injection and LED illumination. This information also demonstrates the presence of 69-L_2_@F at the tumor site throughout the experiment (Figure 6a,c). In contrast, we did not find any fluorescence signal in any of the major organs, meaning that the hydrogel enabled the exclusive accumulation of 69-L_2_@F in the tumor tissue, as demonstrated by the *ex vivo* images of the organs (Figure 6a). Moreover, breast tumors treated either with the hydrogel (with or without LED irradiation) or with 69-L_2_@F-Gel without LED irradiation present adipocytes localized at the invasive tumor front (Figures 6d and S43). Cancer cells often invade the adipose tissue and induce adipocytes to release free fatty acids, which are absorbed by cancer cells and used to produce ATP, thus facilitating tumor growth.^65^ Note that, at the invasive front, the size and number of adipocytes (arrows in Figures 6d and S43) are reduced. It is also important to highlight that the close localization between adipocytes and invasive cancer cells (adipocytes in the vicinity of cancer cells) displays profound phenotypic and functional alterations. The role of adipocytes relies on their support and promotion of tumor growth.^66^ Furthermore, when compared to mice treated with 69-L_2_@F-Gel + LED irradiation, histological images of these solid tumors consistently showed an increase in the invasive front (brackets in Figures 6d and S43) of the tumor with a higher accumulation of active and dividing cells. There are also multifocal to coalescing areas of necrosis (asterisks in Figures 6d and S43) in the tumors treated with 69-L_2_@F-Gel with LED irradiation, corroborating the role of the LED-activated 69-L_2_@F in promoting a PDT that employs exogenously produced ROS to kill breast cancer cells by light activation. Furthermore, tumors from the 69-L_2_@F-Gel + LED-treated mice show much less actively proliferating cells when compared to the Hydrogel + LED group (Figure 6e). This is observed as a decrease in the brown color, which identifies the presence of the *K*_i_-67 protein, a marker for cellular proliferation and rRNA transcription. Likewise, the percentage of inflammatory foci/tumor in the 69-L_2_@F-Gel + LED group is highly significantly increased (*P* < 0.001) when compared to the controls (Figure 6f). This is consistent with ROS production in the treated group, as these molecules can trigger cell-death-associated pathways through necrosis, as we can observe by a slightly significant (*P* < 0.05) when compared to the controls (Figure 6g).

## Conclusions

We demonstrated the development of a novel nanoplatform for hypoxic PDT by combining fluorinated polymers, F-PEG, with a BODIPY-based Zr-MOF, 69-L_2_. While conventional direct solvothermal synthesis failed to produce a BODIPY-based Zr-MOF due to the limited chemical stability of the BODIPY ligand, we demonstrate how a PSE approach allows building a 2-fold interpenetrated UiO-69-type MOF. Importantly, this transformation for incorporating BODIPY into a MOF requires compatible, size-matched ligands. Further, encouraged by our previous PEGylation strategy and to increase the oxygen transport to solid tumors, required for PDT—we synthesized phosphate-functionalized fluoropolymer and performed a modification of 69-L_2_. We employed a series of techniques, including SCXRD, PXRD, N_2_ sorption, SEM, TEM, and DLS, to characterize both micro- and nanosized 69-L_2_, and its PEGylated analogues. In addition, the successful internalization of 69-L_2_, 69-L_2_@P, and 69-L_2_@F into triple-negative breast cancer MDA-MB-231 cells was verified using CLSM and Bio-TEM. Due to the oxygen-carrying ability of the perfluorooctyl group, 69-L_2_@F has been proven to enable hypoxia relief and generate ROS *in vitro* on LED light irradiation. Importantly, 69-L_2_@F exhibited enhanced ROS levels compared to those of the analogue without the perfluorooctyl group, 69-L_2_@P. *In vivo* delivery of 69-L_2_@F embedded in a hydrogel scaffold patch and implanted inside the breast tumor allowed the local and sustained release of the engineered MOFs and efficiently blocked breast cancer proliferation and growth, further improving preclinical outcomes such as survival. By focusing on localized delivery, we ensure that the MOFs have a direct and sustained impact on tumor cells. In contrast to systemic administration, which may dilute the therapeutic agent throughout the body, this localized approach maintains a high concentration of MOFs within the tumor, maximizing their therapeutic effect. Therefore, this work not only represents a mild way to prepare BODIPY-based Zr-MOF and further relieve tumor hypoxia by *in situ* oxygenation but also highlights the *in vivo* delivery strategy that combines the photodynamic action of 69-L_2_@F with a hydrogel, leading to a substantial reduction in tumor growth, thereby plausibly introducing this therapy as a more potent substitute to conventional cancer therapies.

## Supporting Information Available

Materials synthesis, characterization details, supporting data items, and supporting figures (PDF)
3D visualization of z-stack confocal images of 69-L_2_ (MP4)
3D visualization of z-stack confocal images of 69-L_2_-P (MP4)
3D visualization of z-stack confocal images of 69-L_2_-F (MP4)
Live cell imaging of cells under dark over 7 days (MP4)
Live cell imaging of light-treated cells over 7 days (MP4)
