# Supplementary material for: A Fluorinated BODIPY-Based Zirconium Metal–Organic Framework for In Vivo Enhanced Photodynamic Therapy
Source: J Am Chem Soc. 2024 Jan 4;146(2):1644–56. doi: 10.1021/jacs.3c12416 (PMC10797627; doi:10.1021/jacs.3c12416)
Supplement: Supplementary file 1 — ja3c12416_si_001.pdf [file ja3c12416_si_001.pdf]

Supporting Information for:

A Fluorinated BODIPY-Based Zirconium Metal-Organic Framework for *in vivo* Enhanced Photodynamic Therapy

Xu Chen[a], Bárbara B. Mendes[b], Yunhui Zhuang[a], João Conniot[b], Sergio Mercado Argandona[a], Francesca Melle[a], Diana P. Sousa[b], David Perl[c], Alexandru Chivu[d], Hirak K. Patra[d], William Shepard[c], João Conde[b,*] and David Fairen-Jimenez[a,*]

[a]Adsorption & Advanced Materials Laboratory (A^2ML), Department of Chemical Engineering & Biotechnology, University of Cambridge, Philippa Fawcett Drive, Cambridge CB3 0AS, U.K.

[b]ToxOmics, NOVA Medical School, Faculdade de Ciências Médicas, NMS|FCM, Universidade Nova de Lisboa; Lisboa, Portugal.

[c]Synchrotron SOLEIL-UR1, L'Orme des Merisiers, Départementale 128, 91190 Saint-Aubin, France.

[d]Department of Surgical Biotechnology, University College London, London NW3 2PF, UK

*e-mails: joao.conde@nms.unl.pt; df334@cam.ac.uk

Table of Contents

S1. General Experimental Remarks **S2**
S2. General Synthetic Procedures **S7**
S3 Characterizations **S21**
S4 *In Vitro* Study **S28**
S5. *In Vivo* Study **S32**
S6. BET Areas Calculation Using BETSI **S35**
S7. Additional X-ray Crystallographic Structures **S47**
S8 Reference **S50**

S1. General Experimental Remarks

Powder X-ray diffraction (PXRD): PXRD data were collected on a Bruker D8 DAVINCI diffractometer at 298 K using Cu K*α* radiation. The calculated PXRD patterns were produced using the Mercury program and single crystal reflection data.

Thermogravimetric analysis (TGA): TGA measurements were carried out using a TA Instruments Discovery SDT650. Measurements were collected from room temperature to 800 °C with a heating rate of 5 °C / min under nitrogen.

Fourier-transform infrared spectroscopy (FT-IR): FT-IR was carried out using a Bruker Tensor 27 FTIR with the attenuated total reflectance (ATR) method.

Gas uptake: N_2 adsorption isotherm measurements were performed on a Micromeritics 3-Flex analyzer at 77 K. Around 80 mg samples were used for each measurement, Prior to the measurement, all the samples were degassed under vacuum at 50 °C for 24 hours using the internal turbopump. Gas uptakes were performed on the air-dried samples.

Dynamic light scattering (DLS) and zeta potential: Measurements were recorded in an aqueous solution with the sample concentration of around 0.2 mg/ mL by a Zetasizer Nano ZS, (Malvern Instrument Ltd., U.K.) equipped with a He−Ne laser operating at 633 nm at 25 °C. Zeta potential was measured in a folded capillary Zeta cell DTS1070. The Smoluchowski equation was used to calculate the zeta potential. Measurements were performed three times with over 10 subruns for each sample. Error bars represent the standard deviation of three measurements.

Inductively coupled plasma-optical emission spectroscopy (ICP-OES): ICP-OES was performed using a Perkin Elemer ICP-OES Optima 2100DV. Samples were dispersed in 2 mL of nitric acid and 6 mL of hydrochloric acid (*CAUTION!*) and left to stand at room temperature in the fume cupboard for at least 2 h until all reactions have ceased. After that, samples were heated at 120 °C for 10 h to digest the sample completely. After this time, the clear solution was obtained, which was then diluted with Millipore water and analyzed for P and Zr content by comparing it to the standard P and Zr solutions. 3 repeats were performed for each sample.

UV-Vis spectroscopy: UV-vis and fluorescence spectra were recorded using a Tecan Spark® Multimode Microplate Reader.

Liquid nuclear magnetic resonance spectroscopy (NMR) was carried out using a Bruker 400 MHz Avance III HD Smart Probe Spectrometer.

Scanning electron microscopy (SEM): The samples for SEM tests were coated with Pt or Au for 40 seconds and imaged using a FEI Nova Nano SEM 450.

Transmission electron microscopy (TEM): The samples were prepared by dispersing the samples in ethanol using ultrasonication. After that, a small number of suspensions were drop-casted on a copper grid with a carbon support film. A Thermo Scientific (FEI) Talos F200X G2 TEM operating at 200 kV was utilized for TEM, HAADF-STEM and EDS analysis. TEM images were acquired using a Ceta, 4k × 4k CMOS camera. EDS data was collected in STEM mode using the HAADF detector with EDS spectra and maps collected using the Super-X EDS detector system, which consists of 4 windowless silicon drift detectors.

Transmission electron microscopy (TEM) of the stained cells: MDA-MB-231 cells were fixed in 2% glutaraldehyde/2% formaldehyde in 0.05 M sodium cacodylate buffer pH 7.4 containing 2 mM $CaCl_2$ at 4 °C for at least 4 h . Samples were washed 3x in 0.05 M sodium cacodylate buffer pH 7.4 and osmicated for 2h at room temperature with 1% osmiumtetroxide/1.5% potassium ferricyanide in 0.05 M sodium cacodylate buffer pH 7.4. Samples were washed 3x in deionized water and dehydrated in a series of ethanol solutions (50%/70%/95%/100%/100% dry ethanol), 2x in each, followed 2x with acetonitrile. The samples were resin embedded in Quetol resin, with fresh resin each day for 5 days. The resin mix was: 12 g Quetol 651, 15.7 g NSA, 5.7 g MNA and 0.5 g BDMA (all from TAAB). The resin was cured in an embedding oven at 60 °C for 2 days. Thin sections (~ 70 nm) were cut using an ultramicrotome (Leica Ultracut E) and placed on bare 300 mesh copper TEM grids. Samples were post-stained with 2% uranyl acetate/50% methanol for 3 min, followed by Reynold's lead citrate for 6 min. Samples were imaged in a Tecnai G20 TEM (FEI/Thermo Fisher Scientific) run at 200 keV using a 20 um objective aperture to improve contrast. Images were acquired using an ORCA HR high-resolution CCD camera (Advanced Microscopy Techniques Corp, Danvers USA).

Single crystal X-ray diffraction was performed on the PROXIMA 2A micro-focused beamline in SOLEIL synchrotron (λ = 0.729319 Å) using an EIGER X9M 2D hybrid photon counting detector. Data were collected at 100 K. Data integration and reduction were undertaken with *Xia2*[1]. No corrections for solvent scattering have been made. The structure suffers from a significant amount of disorder, some of which is included in the presented model and some which could not be modeled with standard tools. The conformation for the L_2 modeled in the structure represents the majority contributions, as evidenced by the acceptable R_1 value. It is possible that, beyond the currently modeled orientations of L_2, it also exists in reverse orientation, slightly offset from the modeled position. An illustration of this possibility is given in Scheme S1. Due to the pseudo-symmetry of the core of L_2, It is not possible to distinguish this from the modeled orientation at the resolution obtainable at the beamline PROXIMA 2A (approximately 0.7 Å). In addition, it is possible to identify two weak residual electron density peaks at (0.353, 0.652, 0.152) and (0.318, 0.625, 0.125), which may represent the two fluorine atoms in this reverse orientation. When only the reverse orientation is modeled, the R_1 and wR_2 values are higher than the presented model. This kind of disorder cannot be modeled at the same time as the two existing orientations using the standard SHELX tools. This

explains the combination of poor refinement statistics and thermal ellipsoids with very good integration statistics, a stable and chemically sensible model and an absence of confidently identifiable electron density peaks for the other orientations or ligands. The structure was solved using the direct method and refined by full-matrix least-squares on F2 by the SHELXTL-2014 software package. The disorder was modeled using standard crystallographic methods, including constraints, restraints and rigid bodies where necessary. All carbon-bound hydrogen atoms were added in idealized positions and refined using a riding model. DFIX, DELU, SIMU, SADI and SIMU restraints were used to obtain reasonable parameters. H-atoms were refined isotropically, while the other atoms were refined anisotropically. All the phenyl rings are constrained to the ideal six-membered ring. Crystal data and details of the data collection are given in Table S1, while the selected bond distances and angles are presented in Tables S2-S3.

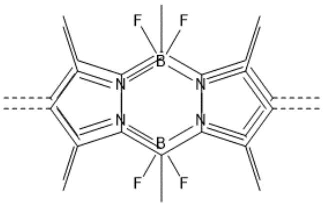

Scheme S1. Illustration of a possible disordered orientation of L_2.

Size exclusion chromatography with multi-angle light scattering (SEC-MLAS) was performed on a Shimadzu HPLC system consisting of an LC-20AD Pump, SIL-20A autosampler, CTO-20A column oven and CBM-20A control unit. The column set was made up of 1 x PSS SUPREMA analytical 100 Å (8 x 300 mm) and 2 x PSS SUPREMA analytical 3000Å (8 x 300 mm). Dual detection was achieved *via* a Wyatt DAWN HELEOS-II multiangle light scattering (MALS) detector (laser at λ = 658 nm), and Wyatt Optilab rEX differential refractive index (DRI) detector with a 658 nm light source. MilliQ Water containing 0.1 mol/L sodium nitrate and 0.01 mol/L sodium azide was used as the eluent at a flow rate of 1.0 mL/min. The column temperature and the detector temperature were kept at 30 °C. All data analysis was performed using Wyatt Astra V 6.1.1 software. A literature value for the dn/dc of poly(ethylene glycol) in water (0.134 mL/g)[2] was used to determine the molecular weight of all samples.

Cell culture: MDA-MB-231 cells were chosen as our *in vitro* and *in vivo* cellular system to evaluate the PDT efficacy of 69-L_2@P and 69-L_2@F against breast cancer cells. MDA-MB-231 cells were maintained at 37°C, and 5% CO_2 in high-rich glucose (4500 mg/L) DMEM (Gibco Dulbecco's Modified Eagle Medium, Gibco® 41965039) supplemented with 10% (v/v) fetal bovine serum (Sigma-Aldrich F9665), 100 units/mL penicillin, and 100 µg/mL streptomycin (Life Technologies 15140122). The supplemented medium is then named the 'complete' medium. PBS (Sigma D8537) and trypsin-EDTA (Life Technologies 25300054) were used to maintain the cell line. For hypoxic cultures, cells were split and incubated under normal conditions (with approximately 18.6% O_2 concentration)[3] for at least 8 h to allow cells to adhere to the bottom of the plates before they were put in a designated

hypoxic incubator with 1% O_2 concentration.

MTS cytotoxicity assay: The concentration (based on 69-L_2)-dependent viability of 69-L_2, 69-L_2@P and 69-L_2@F on MDA-MB-231 cell line were evaluated using MTS assay (Promega, USA). Cells were seeded into 96-well plates at a density of 10 000 cells/well in 100 µL of complete growth medium and incubated at 37 °C, 5% CO_2 for 24 h. Subsequently, 69-L_2, 69-L_2@P and 69-L_2@F were dispersed and diluted in complete medium in a range of concentrations accordingly, of which 100 µL were added to each well and incubated for 72 h at 37 ºC, 5% CO_2 in the dark. At the end of the incubation period for 72 h, cells were washed once with PBS followed by 2 h incubation of diluted MTS solution that was prepared according to manufacturer protocol. The absorbance of each well was measured at 490 nm using a Spark plate reader (TECAN, CH). Control measurements included negative control of cells with DMEM, cells with DMEM containing 1% of water, and cell-free cultured media (blank). All experiments were conducted in biological triplicates.

MTT cytotoxicity assay: The concentration-dependent viability of 69-L_2@P and 69-L_2@F was investigated using the Thiazolyl Blue Tetrazolium Bromide (Sigma, M2128-5G, dissolved as 40 mg/mL stock in DMSO). Briefly, MDA-MB-231 cells were seeded on a 96-well plate at a density of 5000 cells/well for approximately 24 h. 69-L_2@P and 69-L_2@F were dispersed in a complete medium, and a range of concentrations was prepared accordingly, of which 100 µL were added to each well and incubated at either normoxic or hypoxic condition for 24 h. Then, plates were treated with LED light irradiation (525 nm high-power LED, 3.1 W, SOLIS-525C, ThorLabs) for 10 min. Following a further incubation of 48 h, the treatment solutions were removed, and cells were washed once with PBS. 2 mg/mL of final MTT working solution was added to each well, and the contents were incubated for 4 h at 37°C/5% CO_2. After the incubation, the supernatant was removed, and 150 µL of DMSO was added to dissolve the formazan crystals. The resulting solution was then transferred to a clean 96-well plate, and the absorbance was recorded by UV-Vis SPECTROstar Nano at 570 nm. All experimental results were independently carried out three times with five technical repeats in each experiment.

Live cytotoxicity assay: The time-dependent cytotoxicity of 69-L_2, 69-L_2@P, and 69-L_2@F (100 µg/mL based on 69-L_2) in MDA-MB-231 cell line was evaluated using the IncuCyte®S3 Live Cell Analysis System (Sartorius). Cells were seeded into two 96-well plates at a density of 10000 cells/well in 100 µL of complete growth medium and incubated at 37 °C, 5% CO_2 for 24 h. Subsequently, different concentrations of 69-L_2, 69-L_2@P, and 69-L_2@F were diluted in a complete medium containing 250 nM of Incucyte® Cytotox Dye and incubated at 37 ºC, 5% CO_2. After 24h, one plate was treated with LED light irradiation using Lumidox® Gen II 96-Well LED Arrays at 527 nm wavelength for 10 minutes. Both light and non-light-treated plates were imaged every 3 h for 7 days under cell culture conditions with 10× objective using the brightfield and the red channels. Mean cell confluence was calculated using the images taken from 3 random fields of view per well using

the IncuCyte S3 v2022A software. All Incucyte experiments were performed in triplicate. Relative confluence values were obtained by dividing the Cytotox red signal by the total confluence and normalizing each value to the time zero value in each sample.

Flow cytometry: MDA-MB-231 cells were seeded on to 6-well plate the day before they were treated with 69-L_2@P and 69-L_2@F at a concentration of 25 µg/mL (based on 69-L_2) to each well for 24 h. Then, 10 µM of DCF-DA were added to each well and incubated for 2 h before their LED light (3.1 W, SOLIS-525C, ThorLabs) irradiation for 10 min. Following their light irradiation, cells were washed with PBS and detached from the plate using trypsin for 3 min at 37°C/5% CO_2. The cell pellets were then collected and washed with PBS twice by centrifugation and fixed on ice with 4% paraformaldehyde (PFA) for immediate acquisition on the same day. Unstained and single stain compensation tubes, with and without MOFs treatment, were also prepared for spectral overlap compensation. The samples are then acquired using BD LSRFortessa which is equipped with three lasers (405, 488 and 642 nm) in standard configuration and standard filter sets. FlowJo v10.8.1 was used for analysis.

Confocal microscopy: MDA-MB-231 Cells were seeded into an 8-well Nunc™ Lab-Tek™ II Chamber Slide™ System Measurements at a concentration of 100 000 cells/mL and incubated at 37 °C overnight. Subsequently, the cells were treated with water (control) or 69-L_2, 69-L_2@P and 69-L_2@F at a concentration of 100 µg/mL based on 69-L_2 for 24 h. For the internalization experiment, cells were then washed twice with 1xPBS and stained with CellMask™ Red (Thermo Fischer) Plasma membrane stain and Hoechst 33342 (Thermo Fisher) according to the manufacturer's instructions. For the ROS generation confocal experiment, cells were washed with 1xPBS and incubated with DCF for 2 h. Subsequently, one plate was left in dark conditions for the duration of the experiment, while another plate was treated with LED light irradiation for 10 minutes. Cells were washed three times with 1xPBS, left in growth media, and imaged using a confocal microscope (Axio Observer Z1 LSM 800, Zeiss). The 405 nm, 488 nm, 561 nm, and 640 nm lasers were used to excite MOFs and stains. Images were collected using an oil immersion 63X/1.4NA lens. Zen software (Zeiss) was used for acquisition and image processing.

S2. General Synthetic Procedures

All reagents, unless otherwise stated, were purchased from Sigma Aldrich, Fluorochem, or Alfa Aesar and used as provided. 2',3''-dimethyl-[1,1':4',1'':4'',1''']-quaterphenyl]-4,4'''-dicarboxylic acid (**L_1**), **S1,** Zr_6 cluster and P-PEG were synthesized according to a previously reported procedure.[4-7] Milli-Q water (18.2 MΩ.cm resistivity at 25 °C) was used throughout the experiment. Dialysis tubing (Molecular Weight Cut-off; MWCO 3,500 and12,000 - 14,000 Daltons) was obtained from Medicell Membranes Ltd. 525 nm high-power LED (3.1 W, SOLIS-525C, ThorLabs) was used for light irradiation. The concentrations of MOF samples used in the *in vitro* and *in vivo* studies were calculated based on the amount of bare 69-L_2.

Synthesis of L_2

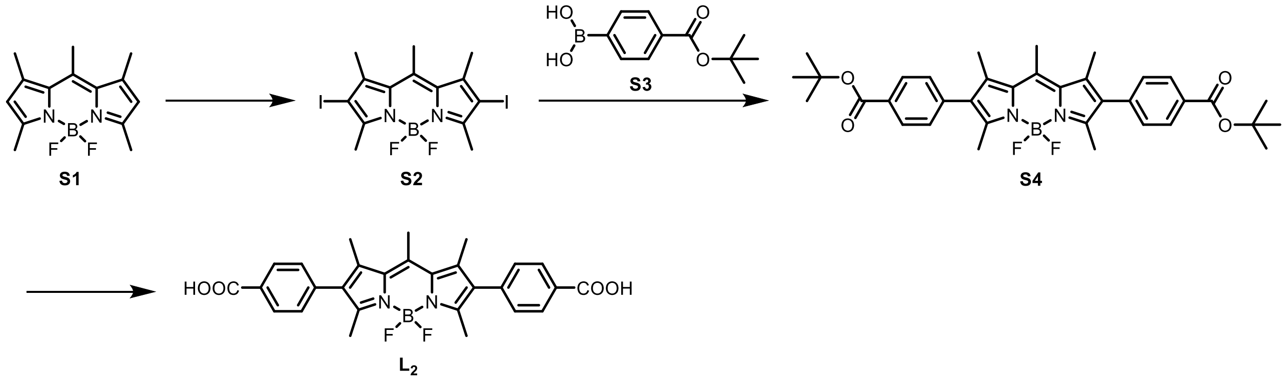

Scheme S2. Synthesis of L_2.

Synthesis of S2. To a flame-dried flask was added with **S1** (1.05 g, 4 mmol), NIS (1.8 g, 8 mmol), and anhydrous DCM (120 mL). The mixture was stirred at room temperature for 24 h. Afterward, the mixture was concentrated under reduced pressure, and the crude product was purified by column chromatography on silica gel (DCM/cyclohexane, 1:1 v/v) to give compound **S2** as a red solid (1.75 g, 85%). ^1H NMR (400 MHz, $CDCl_3$): δ 2.62 (s, 3H), 2.60 (s, 6H), 2.46 (s, 6H).

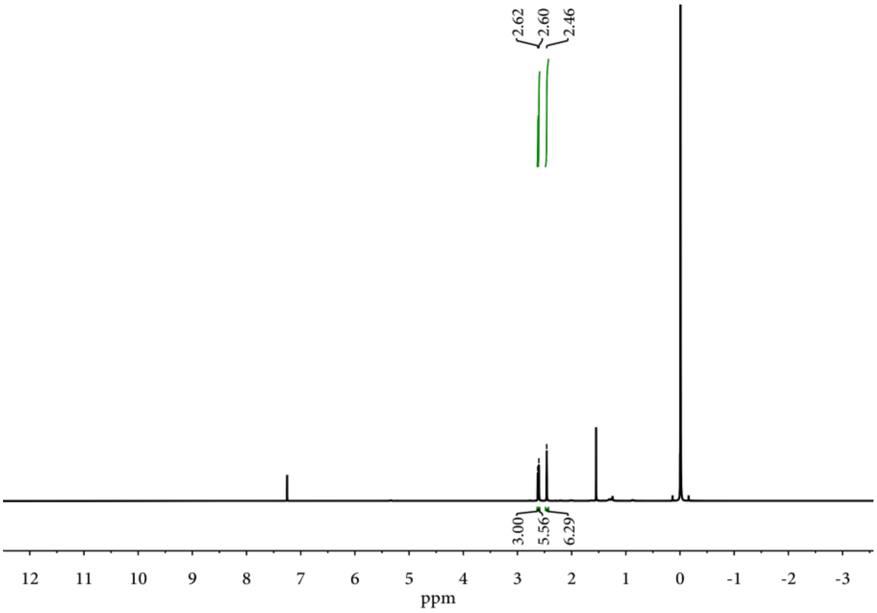

Figure S1. ^1H NMR spectrum of **S2**.

Synthesis of S4. A 100 mL flame-dried round-bottom flask was charged with **S2** (1.29 g, 2.5 mmol), **S3** (1.94 g, 8.75 mmol, 3.5 equiv), K_2CO_3 (2.07 g, 15 mmol, 6 equiv) and $Pd(PPh_3)_4$ (289 mg, 0.25 mmol), followed by addition of the degassed dioxane (30 mL) and H_2O (6 mL). The resulting suspension was heated at 90 °C for 24 h. After cooling to room temperature, water was added. The resulting mixture was then extracted with DCM * 3 times and washed with water. The combined organic extracts were dried over Na_2SO_4 and then concentrated under reduced pressure. The crude solid was purified by column chromatography on silica gel (EtOAc/cyclohexane, 1:10, v/v) to afford **S4** as a red solid (936 mg, 61%). ^1H NMR (400 MHz, $CDCl_3$): δ 8.07 (d, *J* = 8.3 Hz, 4H), 7.29 (d, *J* = 8.4 Hz, 4H), 2.73 (s, 3H), 2.49 (s, 6H), 2.35 (s, 6H), 1.62 (s, 18H). ^{13}C NMR (101 MHz, $CDCl_3$): δ 165.56, 152.36, 142.27, 138.15, 137.30, 132.87, 132.40, 130.79, 130.22, 129.52, 81.12, 28.23, 17.35, 15.52, 13.28.

HRMS-ESI (m/z): Calculated for **S4**: $C_{36}H_{41}BN_2O_4F_2$: 614.3127, Found: 614.3148.

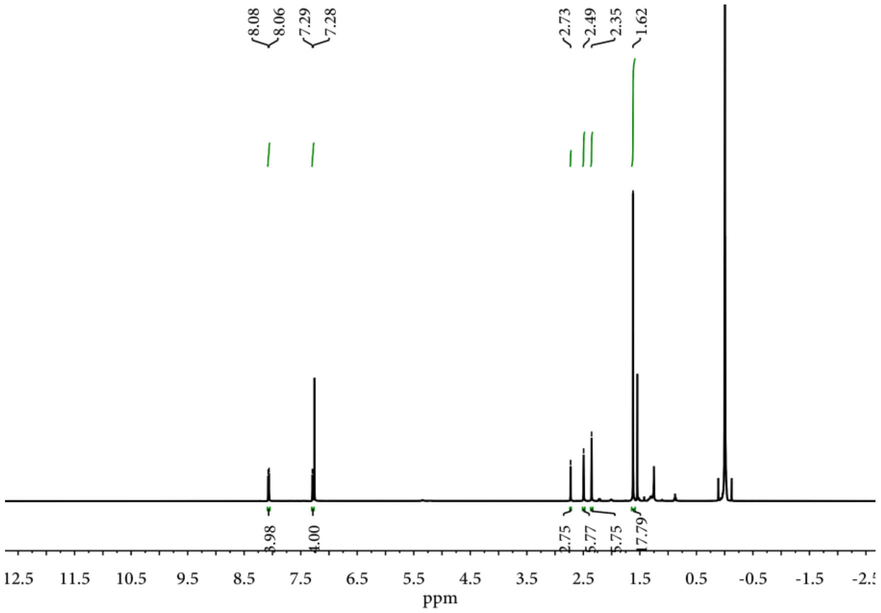

Figure S2. ^1H NMR spectrum of **S4**.

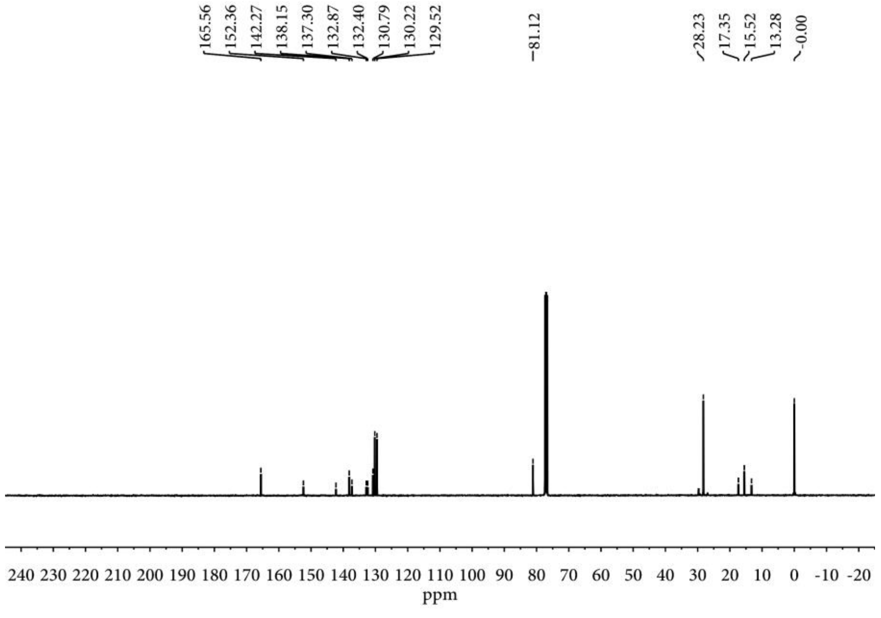

Figure S3. ^{13}C NMR spectrum of **S4**.

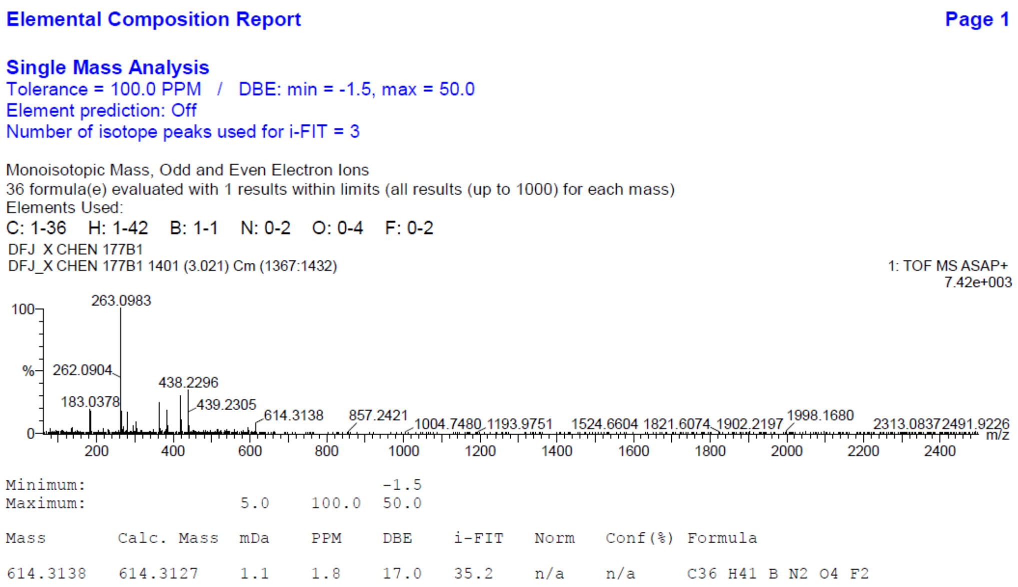

Figure S4. HRMS-ESI spectrum of **S4**

Synthesis of L$_2$: To a solution of **S4** (660 mg, 1.07 mmol) in dry CH_2Cl_2 (35 mL) was added trifluoroacetic acid (15 mL) at 0 ºC. The reaction mixture was allowed to stand at 0 ºC for 5 h, and then stirred at room temperature overnight. Afterward, the solvent was removed with the flowing nitrogen. The resulting residue was then washed with Et_2O (30 mL) * two times, affording **L2** as a red solid (483 mg, 90 %). ^{1}H NMR (400 MHz, DMSO-d_6): δ 8.04 (d, J = 8.3 Hz, 4H), 7.45 (d, J = 8.3 Hz, 4H), 2.79 (s, 3H), 2.43 (s, 6H), 2.39 (s, 6H). ^{13}C NMR (126 MHz, DMSO-d_6): δ 167.55, 151.68, 144.48, 138.36, 138.04, 132.40, 132.23, 130.82, 129.93, 129.90, 17.68, 15.62, 13.57.
HRMS-ESI (m/z): Calculated for [**L$_2$** + H]$^+$, $C_{28}H_{26}BF_2N_2O_4$: 503.1954, Found: 503.1954.

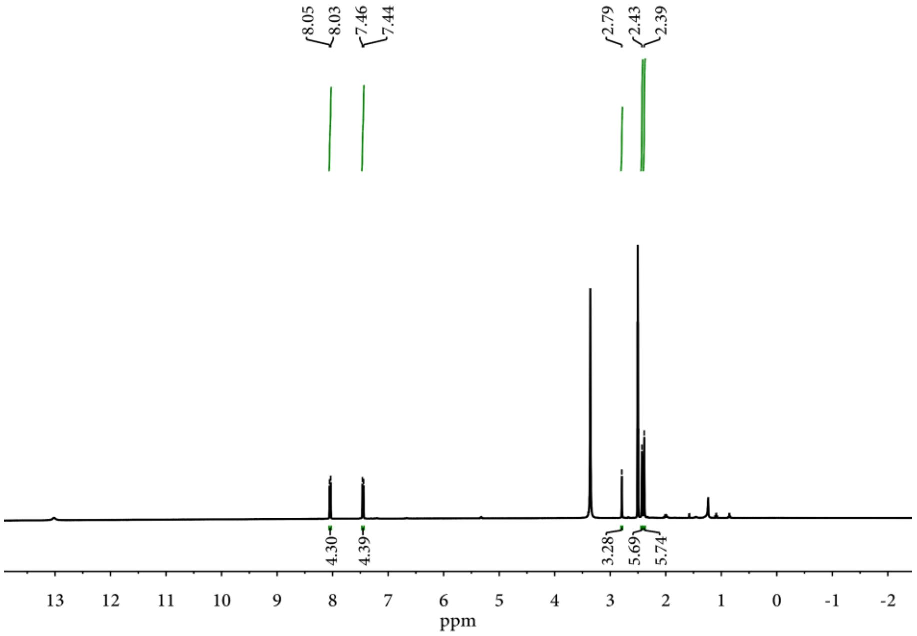

Figure S5. ^{1}H NMR spectrum of **L$_2$**.

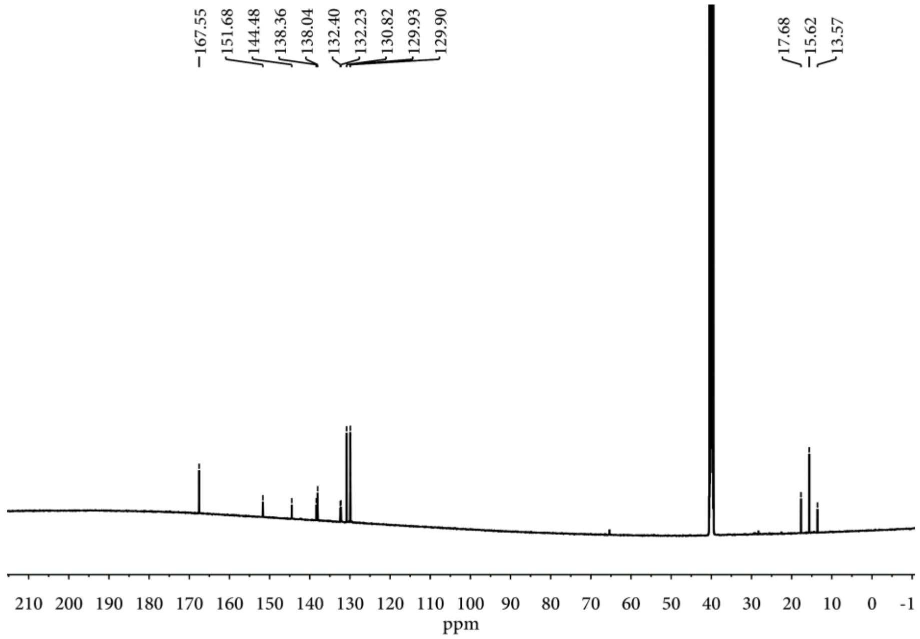

Figure S6. ^{13}C NMR spectrum of **L$_2$**.

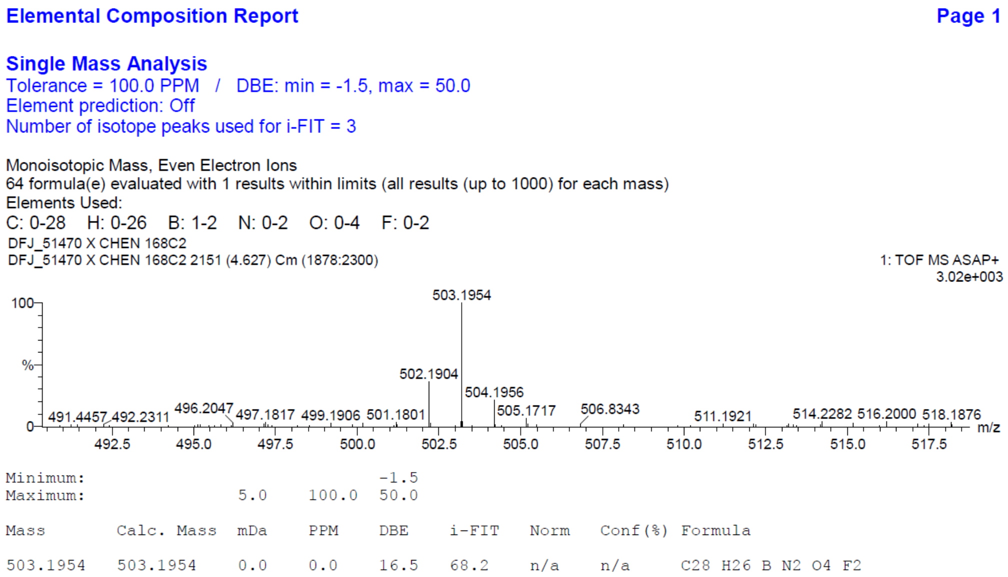

Figure S7. HRMS-ESI spectrum of **L$_2$**

Synthesis of L$_3$

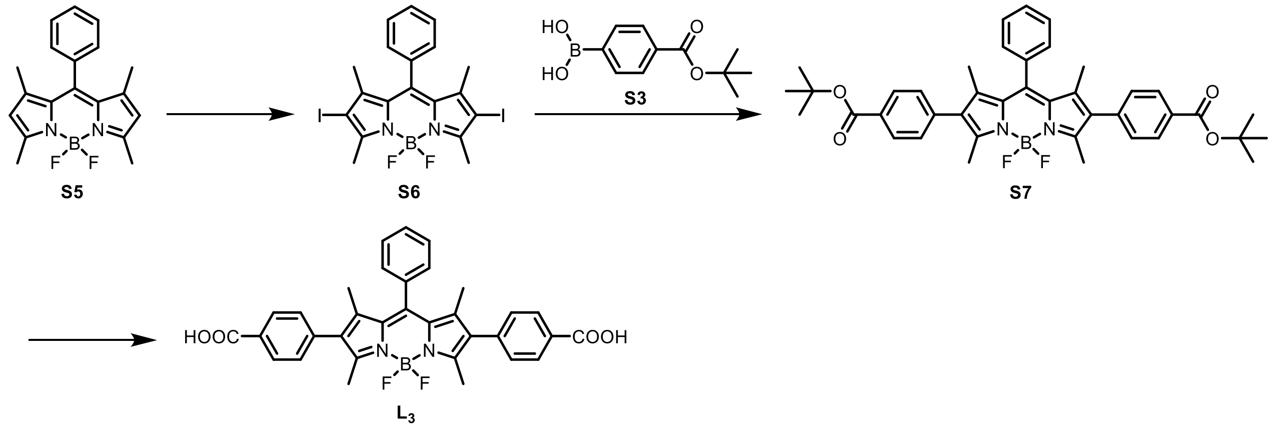

Scheme S3. Synthesis of **L$_3$**.

Synthesis of S6.[8] To a flame-dried flask was added with **S5** (648 mg, 2 mmol), NIS (900 mg, 4 mmol), and anhydrous DCM (80 mL). The mixture was stirred at room temperature for 24 h. Afterward, the mixture was concentrated under reduced pressure, and the crude product was purified by column chromatography on silica gel (DCM/cyclohexane, 1:2 v/v) to give compound **S6** as a red solid (933 mg, 81%). ^{1}H NMR (700 MHz, $CDCl_3$): δ 7.56 – 7.53 (m, 3H), 7.28 – 7.26 (m, 2H), 2.67 (s, 6H), 1.41 (s, 6H).

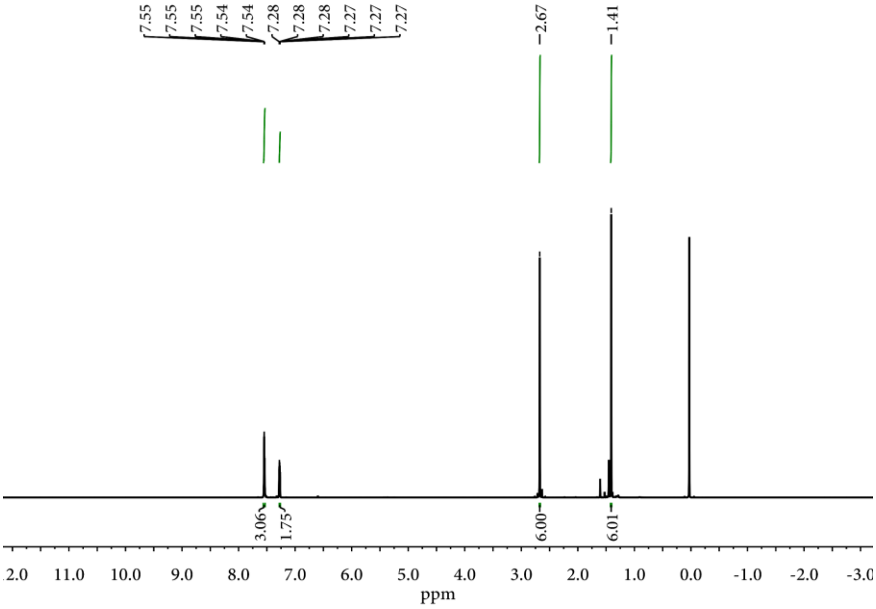

Figure S8. ^{1}H NMR spectrum of **S6**.

Synthesis of S7. A 150 mL flame-dried round-bottom flask was charged with **S6** (1.29 g, 4.3 mmol), **S3** (5.3 g, 4 equiv), K_2CO_3 (3.6 g, 6 equiv) and $Pd(PPh_3)_4$ (497 mg, 0.43 mmol), followed by addition of the degassed dioxane (60 mL) and H_2O (12 mL). The resulting suspension was heated at 90 °C for 36 h. After cooling to room temperature, water was added. The resulting mixture was then extracted with DCM * two times and washed with water. The combined organic extracts were dried over Na_2SO_4 and then concentrated under reduced pressure. The crude solid was purified by column chromatography on silica gel (EtOAc/DCM/cyclohexane, 1:1:30, v/v/v) to afford **S7** as a red solid (1.42 g, 49%). ^{1}H NMR (700 MHz, $CDCl_3$): δ 8.04 (d, *J* = 8.0 Hz, 4H), 7.52 (dd, *J* = 13.1, 7.1 Hz, 3H), 7.39 – 7.37 (m, 2H), 7.25 (d, J = 8.0 Hz, 4H), 2.57 (s, 6H), 1.63 (s, 18H), 1.34 (s, 6H). ^{13}C NMR (176 MHz, $CDCl_3$): δ 165.56, 154.21, 142.59, 139.47, 138.04, 135.13, 133.00, 131.46, 130.72, 130.23, 129.99, 129.45, 129.36, 129.23, 127.92, 81.10, 28.21, 13.40, 12.74.

HRMS-ESI (m/z): Calculated for **S7**: $C_{41}H_{43}BF_2N_2O_4$: 676.3284, Found: 676.3273.

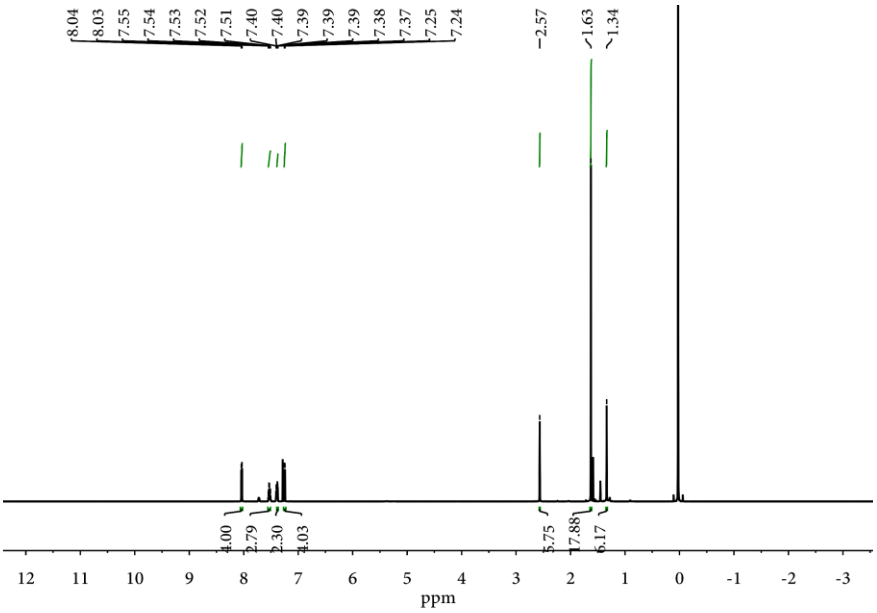

Figure S9. ^{1}H NMR spectrum of **S7**.

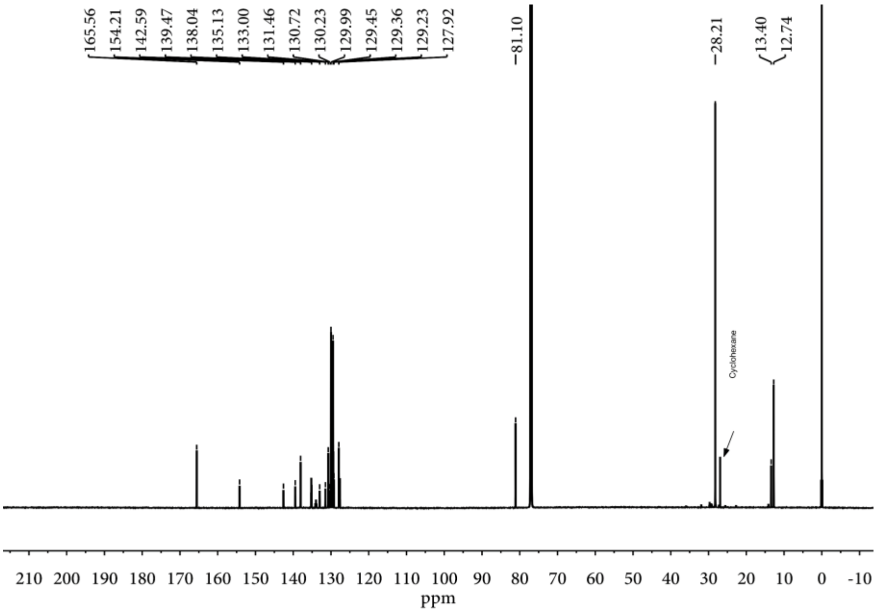

Figure S10. ^{13}C NMR spectrum of **S7**.

Elemental Composition Report **Page 1**

Single Mass Analysis
Tolerance = 100.0 PPM / DBE: min = -1.5, max = 50.0
Element prediction: Off
Number of isotope peaks used for i-FIT = 3

Monoisotopic Mass, Odd and Even Electron Ions
28 formula(e) evaluated with 1 results within limits (up to 50 closest results for each mass)
Elements Used:
C: 0-41 H: 0-44 B: 1-1 N: 0-2 O: 0-4 F: 0-2
X CHEN 52767 1190 2171 (4.665) Cm (2092:2181) 1: TOF MS ASAP+ 3.49e+003

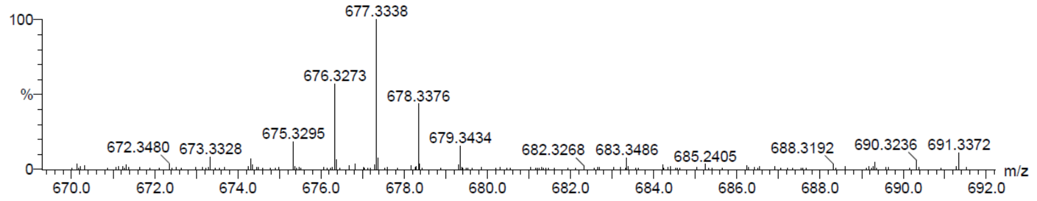

	Mass	Calc. Mass	mDa	PPM	DBE	i-FIT	Norm	Conf(%)	Formula
Minimum:					-1.5				
Maximum:			5.0	100.0	50.0				
	676.3273	676.3284	-1.1	-1.6	21.0	176.0	n/a	n/a	C41 H43 B N2 O4 F2

Figure S11. HRMS-ESI spectrum of **S7**

Synthesis of L$_3$: To a solution of **S7** (846 mg, 1.5 mmol) in dry CH_2Cl_2 (50 mL) was added trifluoroacetic acid (20 mL) at 0 ºC. The reaction mixture was allowed to stand at 0 ºC for 5 h, and then stirred at room temperature overnight. Afterward, the solvent was removed with the flowing nitrogen. The resulting residue was then washed with Et_2O (50 mL) * two times, affording **L3** as a red solid (719 mg, 85 %). ^{1}H NMR (500 MHz, DMF-d_7): 13.33 (s, 2H), 8.11 (d, J = 8.0 Hz, 4H), 7.66 (dd, J = 14.7, 7.1 Hz, 3H), 7.62 – 7.58 (m, 2H), 7.48 (d, J = 8.0 Hz, 4H), 2.59 (s, 6H), 1.42 (s, 6H). ^{13}C NMR (126 MHz, DMF-d_7) δ 167.41, 154.18, 143.60, 139.74, 138.02, 134.98, 132.92, 131.50, 130.53, 130.11, 129.82, 129.81, 129.76, 128.44, 13.19, 12.64.

HRMS-ESI (m/z): Calculated for [**L$_3$** + H]$^+$, $C_{33}H_{28}BF_2N_2O_4$: 565.2110, Found: 565.2082.

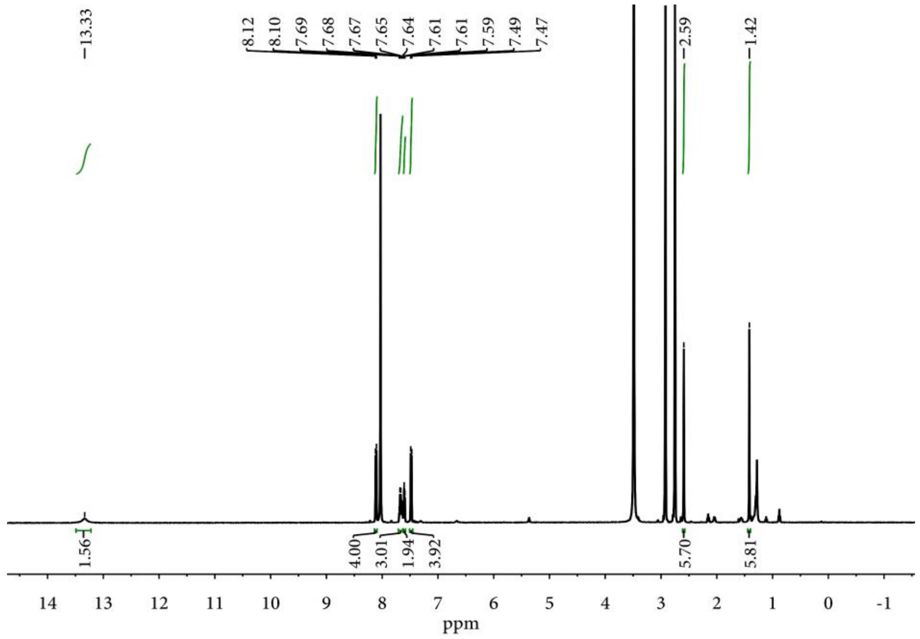

Figure S12. ^{1}H NMR spectrum of **L$_3$**.

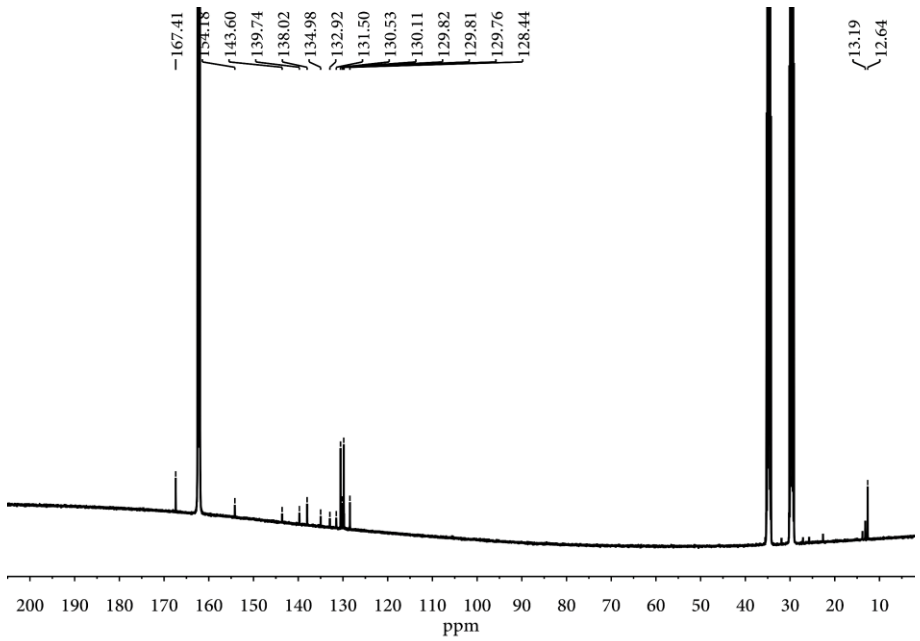

Figure S13. ^{13}C NMR spectrum of **L_3**.

Elemental Composition Report **Page 1**

Single Mass Analysis
Tolerance = 100.0 PPM / DBE: min = -1.5, max = 50.0
Element prediction: Off
Number of isotope peaks used for i-FIT = 3

Monoisotopic Mass, Even Electron Ions
29 formula(e) evaluated with 1 results within limits (up to 50 closest results for each mass)
Elements Used:
C: 0-33 H: 0-28 B: 1-1 N: 0-2 O: 0-4 F: 0-2
X CHEN 52768 1190B 1987 (4.275) Cm (1940:2049) 1: TOF MS ASAP+ 4.17e+003

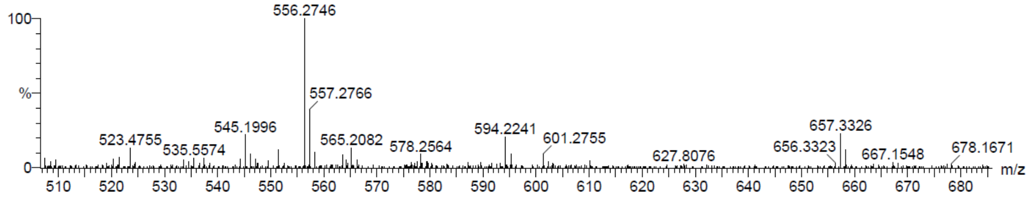

Mass	Calc. Mass	mDa	PPM	DBE	i-FIT	Norm	Conf(%)	Formula
Minimum:				-1.5				
Maximum:		5.0	100.0	50.0				
565.2082	565.2110	-2.8	-5.0	20.5	66.7	n/a	n/a	C33 H28 B N2 O4 F2

Figure S14. HRMS-ESI spectrum of **L_3**

Synthesis of F-PEG

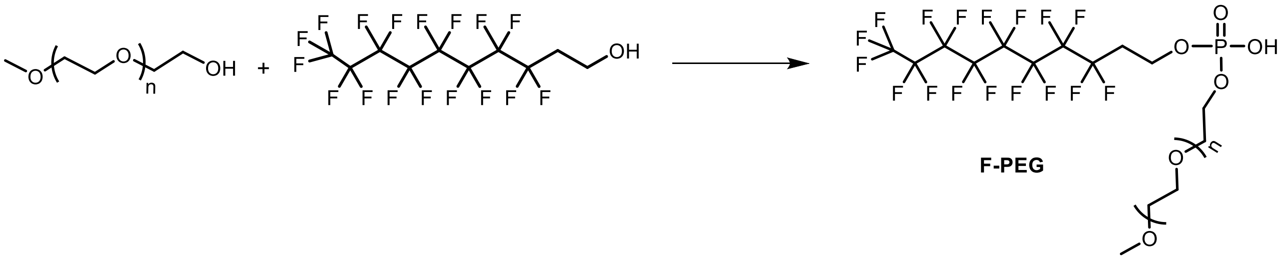

Scheme S4. Synthesis of **F-PEG.**

The synthesis of **F-PEG** was adapted from a reported method with slight modification.[9] To a flamed-dried two-neck flask, phosphorus oxychloride (1.6 mL, 17.2 mmol) in 40 mL of dry DCM was added, and the mixture was cooled to 0 °C. Afterward, 1*H*,1*H*,2*H*,2*H*-perfluoro-1-decanol (7.89 g, 17.0 mmol) and dry triethylamine (51.6 mmol) in 20 mL DCM were slowly added via syringe, leading to the formation of white precipitate. The mixture was then stirred at 0 °C for 1.5 h. After that, poly(ethylene glycol) methyl ether (Mn = 5000, 17 mmol) in dry DCM (20 mL) was added. The resulting solution was allowed to warm to room temperature and stirred under N_2 overnight. The white precipitate was filtered and washed with dry DCM (50 mL). Water (20 mL) was added to the filtrate and then was stirred at 40 °C overnight. Afterward, DCM was removed using the flowing N_2, and purified by dialysis (MWCO: 1,000), followed by removing the residual water with lyophilization. The crude solid was purified by column chromatography on silica gel (DCM/MeOH, 10:1, v/v) to afford **F-PEG** as a white solid (10 mmol, 59%). ^{1}H NMR (400 MHz, $CDCl_3$): δ 4.20 (q, J = 7.0 Hz, 2H), 3.55 (s, 471H), 3.28 (s, 3H), 2.45 (ddd, J = 18.4, 12.2, 6.5 Hz, 2H). ^{13}C NMR ($CDCl_3$, 101 MHz): δ 70.54. ^{31}P NMR ($CDCl_3$, 162 MHz): δ -4.04. ^{19}F NMR ($CDCl_3$, 376 MHz): δ -85.53, -118.33, -126.58, -127.47, -128.37, -130.86.

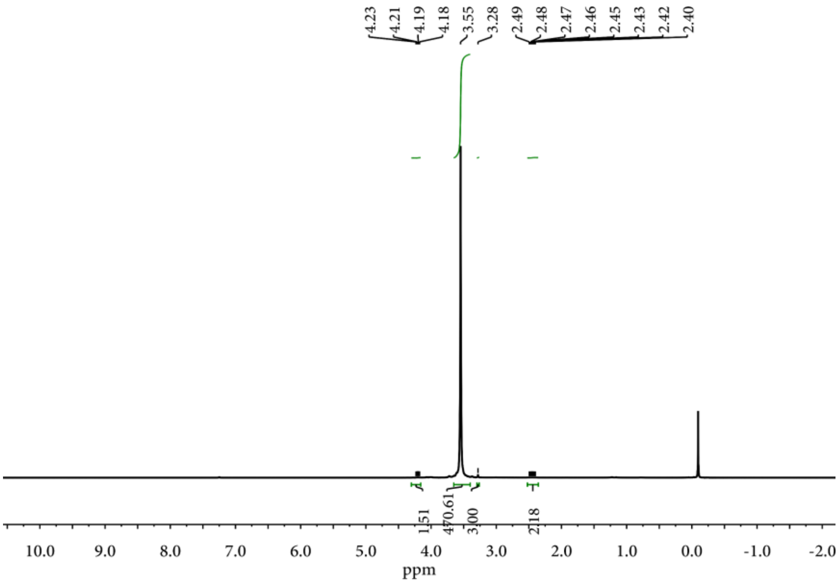

Figure S15. ^{1}H NMR spectrum of F-PEG.

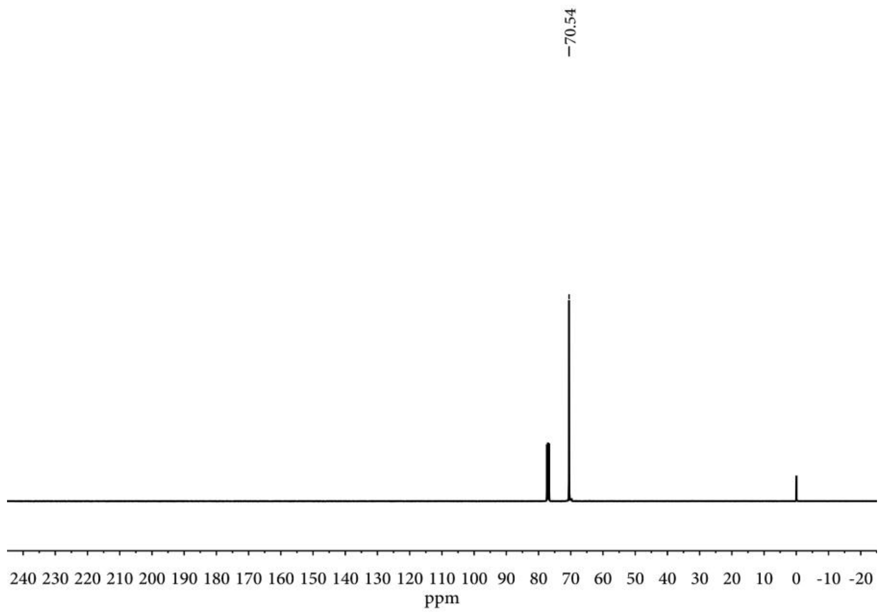

Figure S16. ^{13}C NMR spectrum of F-PEG.

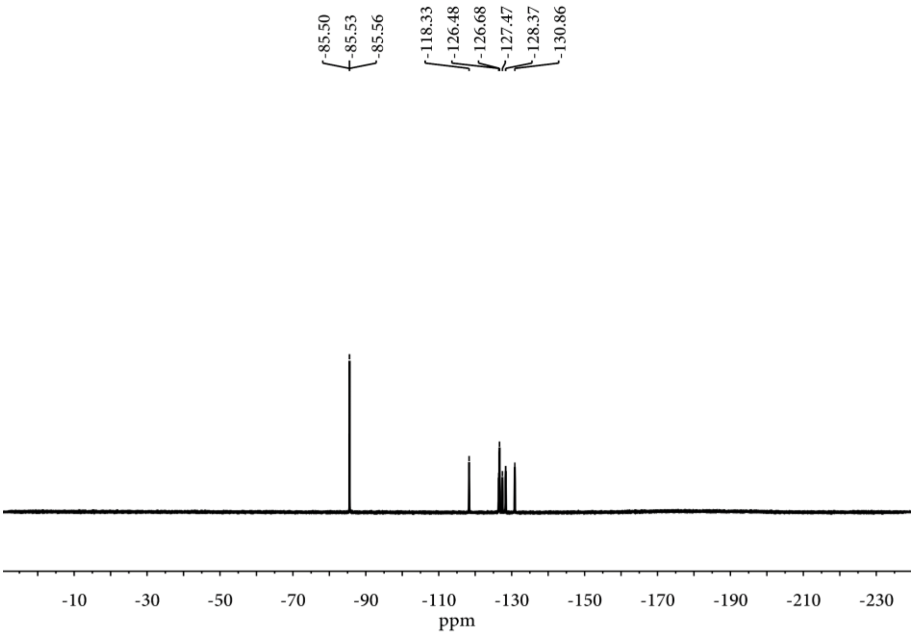

Figure S17. ^{19}F NMR spectrum of F-PEG.

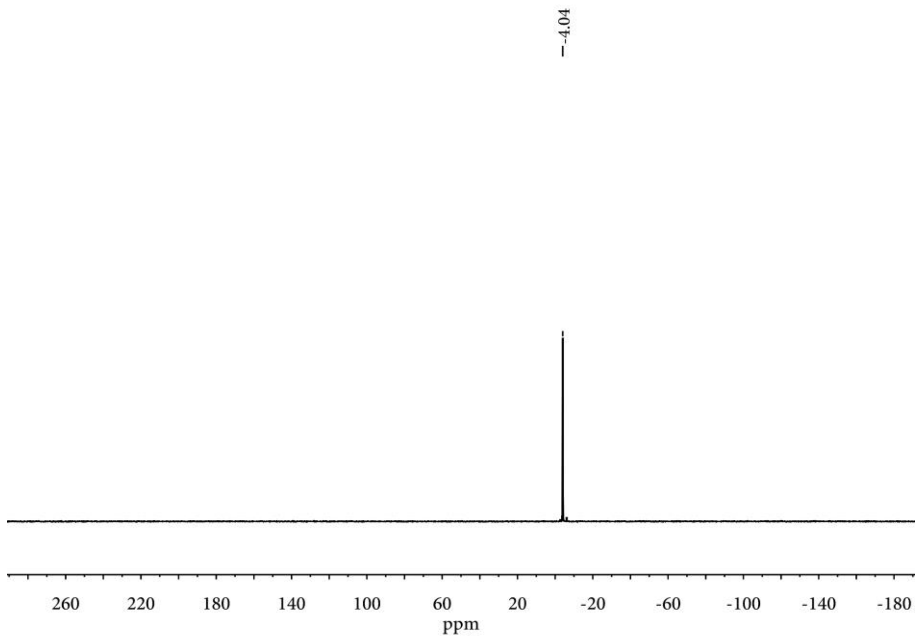

Figure S18. ^{31}P NMR spectrum of F-PEG.

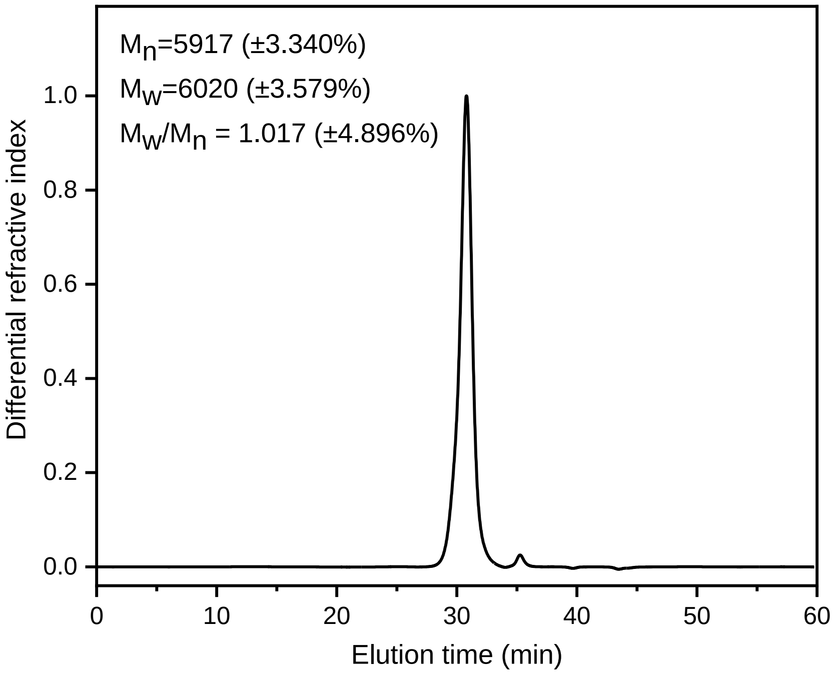

Figure S19. GPC trace of F-PEG

Synthesis of BODIPY-based MOFs

Synthesis of 69-Me$_2$ single crystal (SC 69-Me$_2$): 69-Me$_2$ was synthesized following a previously reported procedure.[5] To a 20 mL vial was added with $\mathbf{L_1}$ (36 mg), $ZrOCl_2 \cdot 8H_2O$ (28 mg), acetic acid (0.982 mL) and DMF (5 mL). The resulting mixture was sonicated for 10 min and then heated at 120 °C for 48 h. After cooling down to room temperature, colorless crystals of 69-Me$_2$ were harvested by filtration.

Synthesis of 69-Me_2 nanoparticle (69-Me_2): To a 20 mL vial was added with L_1 (18 mg), Zr_6O_8 cluster (30 mg), acetic acid (150 µL) and DMF (8 mL). The resulting mixture was sonicated for 10 min and then heated at 120 °C for 12 h. After cooling to room temperature, the obtained sample was collected by high-speed centrifugation (15,000 rpm, 35 min), followed by washing with hot DMF 3 times and exchanging with ethanol 3 times. The final product was dried or re-dispersed in ethanol for further use.

Synthesis of 69-L_2 and 69-L_3: 69-L_2 and 69-L_3 was synthesized by post-synthetic ligand exchange. Briefly, crystals or powders of 69-Me_2 (30 mg) were incubated in the DMF solution (4 mL) of $\mathbf{L_2}$ or $\mathbf{L_3}$ (100 mg) at 70°C for 4 day. The supernatant was exchanged with the fresh solution of $\mathbf{L_2}$ or $\mathbf{L_3}$ (100 mg) in DMF (4 mL) every 12 h. 69-L_2 and 69-L_3 in red color was harvested by centrifugation.

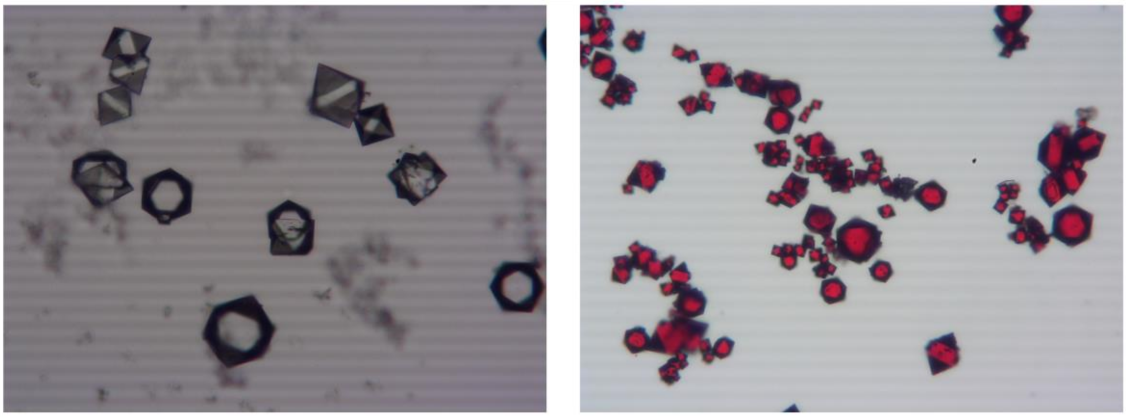

Figure S20. Optical microscope image of Me_2 (left) and 69-L_2 (right) crystals

Synthesis of 69-L_2@P: P-PEG solution (15 mL, 25 mg/mL in H_2O) was added into the aqueous suspension of 69-L_2 (5 mL, 10 mg/mL). After stirring at room temperature overnight, the reaction mixture was centrifuged (25 min, 16,000 rpm) to remove the unreacted P-PEG, and washed with fresh water three times. The final product, denoted as 69-L_2@P, was kept in water. The amount of P-PEG was determined by ICP-OES (P/Zr) and confirmed by TGA. The amount of P-PEG (wt %) = mass of encapsulated P-PEG/ 69-L_2@P × 100.

Synthesis of 69-L_2@F: F-PEG solution (15 mL, 25 mg/mL in H_2O) was added into the aqueous suspension of 69-L_2 (5 mL, 10 mg/mL). After stirring at room temperature overnight, the reaction mixture was centrifuged (25 min, 16,000 rpm) to remove the unreacted F-PEG, and washed with fresh water three times. The final product, denoted as 69-L_2@F, was kept in water. The amount of F-PEG was determined by ICP-OES (P/Zr) and confirmed by TGA. The amount of F-PEG (wt %) = mass of encapsulated F-PEG/69-L_2@F × 100.

The digestion of 69-L_2 samples

To a 6 mL vial, the activated 69-L_2 (10 mg) was stirred with 10X PBS (4 mL) at 50 °C for 7 days. Afterward, the solvent was removed under the vacuum. The residue was then incubated with 5M NH_4F (5 mL) at 50 °C for another 3 days. DMSO-d_6 (0.7 mL) was added after removing the water, and the mixture was sonicated until fully dissolved, which was used for ^1H NMR measurement.

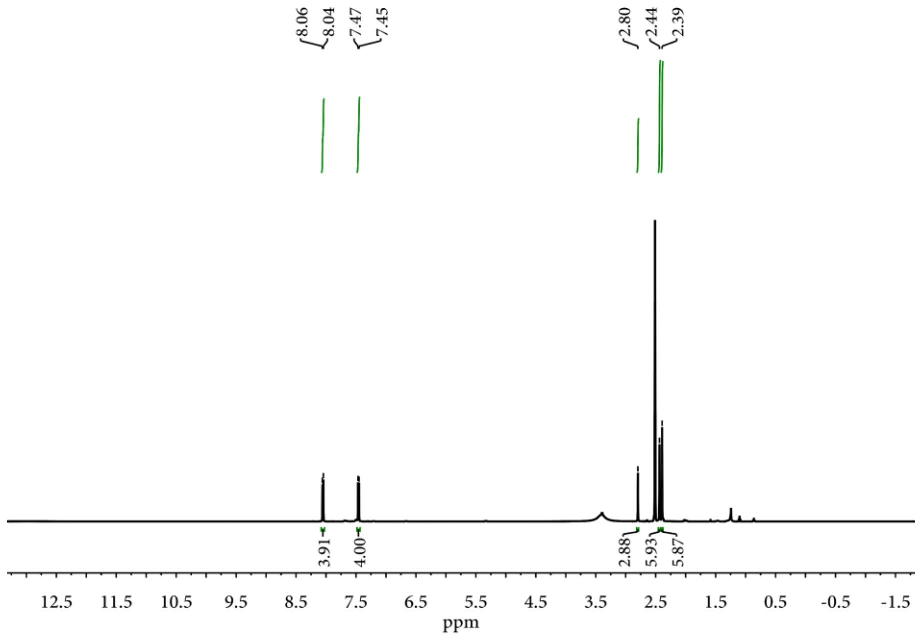

Figure S21. ^1H NMR spectrum of the digested SC 69-L_2.

69-L_2@F-Hydrogel (69-L_2@F-Gel) preparation. Hydroxyethylcellulose (HEC, Natrosol 250 HX pharma, Ashland, Spain) (1.25% w/v) was prepared in ultrapure water and sterilized under UV light for 15 minutes. Then, 1 mL of a stock solution of 5 mg/ mL 69-L_2@F was added to the hydrogel (69-L_2@F final concentration of 2.5 mg/mL), mixed thoroughly by vortexing, and stored at 4°C protected from light until further use.

69-L_2@F-Hydrogel (69-L_2@F-Gel) microstructure. After casting into the molds, only hydrogel or 69-L_2@F-Gel were frozen at −80 °C and subsequently freeze-dried. Moreover, the samples were freeze-fractured, after immersion in liquid nitrogen, to expose their inner structures and sputter coated (10 nm, Quorum) with iridium prior to observation in a scanning electron microscope (Hitachi, Japan). Upon SEM image acquisition, the EDS analysis was performed in the same equipment.

Evaluation of the Oxygen Loading Capability of 69-L_2@P and 69-L_2@F

Milli-Q water was firstly bubbled with the flowing O_2 to saturate the content of O_2. 69-L_2@P and 69-L_2@F were then diluted with the above Milli-Q water to 220 mL with a concentration of 2 mg/mL in terms of 69-L_2. We assume that 69-L_2@F, 69-L_2@P, and water had the same initial amount of O_2, which decreases over time due to the diffusion of dissolved O_2 into the air. The capped suspensions were then stabilized for 1 min. Afterward, the diluted suspensions were uncapped and stirred mildly, where the O_2 concentration was monitored using a portable dissolved oxygen meter (Hanna

Instruments HI-2004-02 Edge® Dissolved Oxygen Meter) for 30 min. The values were recorded automatically every 30 s.

Evaluation of ROS Generation Capability of 69-L_2@P and 69-L_2@F

The time-dependent ROS generation capabilities of 69-L_2@P, 69-L_2@F, and the control group were determined by a chemical acceptor, 1,3-diphenylisobenzofuran (DPBF, 97%, Acros Organics), under the LED light treatment (525 nm high-power LED, 3.1 W, SOLIS-525C, ThorLabs). The solvents were bubbled with flowing oxygen for 30 min before sample preparation. Briefly, DPBF (50 μM) was irradiated with stirring at 525 nm in the presence of the sample (20 μM based on 69-L_2) in MeOH (20 mL) at room temperature with the reaction vial open to the air. Aliquots of the reaction (100 μL) were taken every 30 s and measured by UV-Vis spectroscopy. The decrease of the λ_{max} of DBPF (410 nm) was then evaluated and plotted according to A/A_0 *vs*. time.

S3. Characterizations

SEM and TEM Imaging

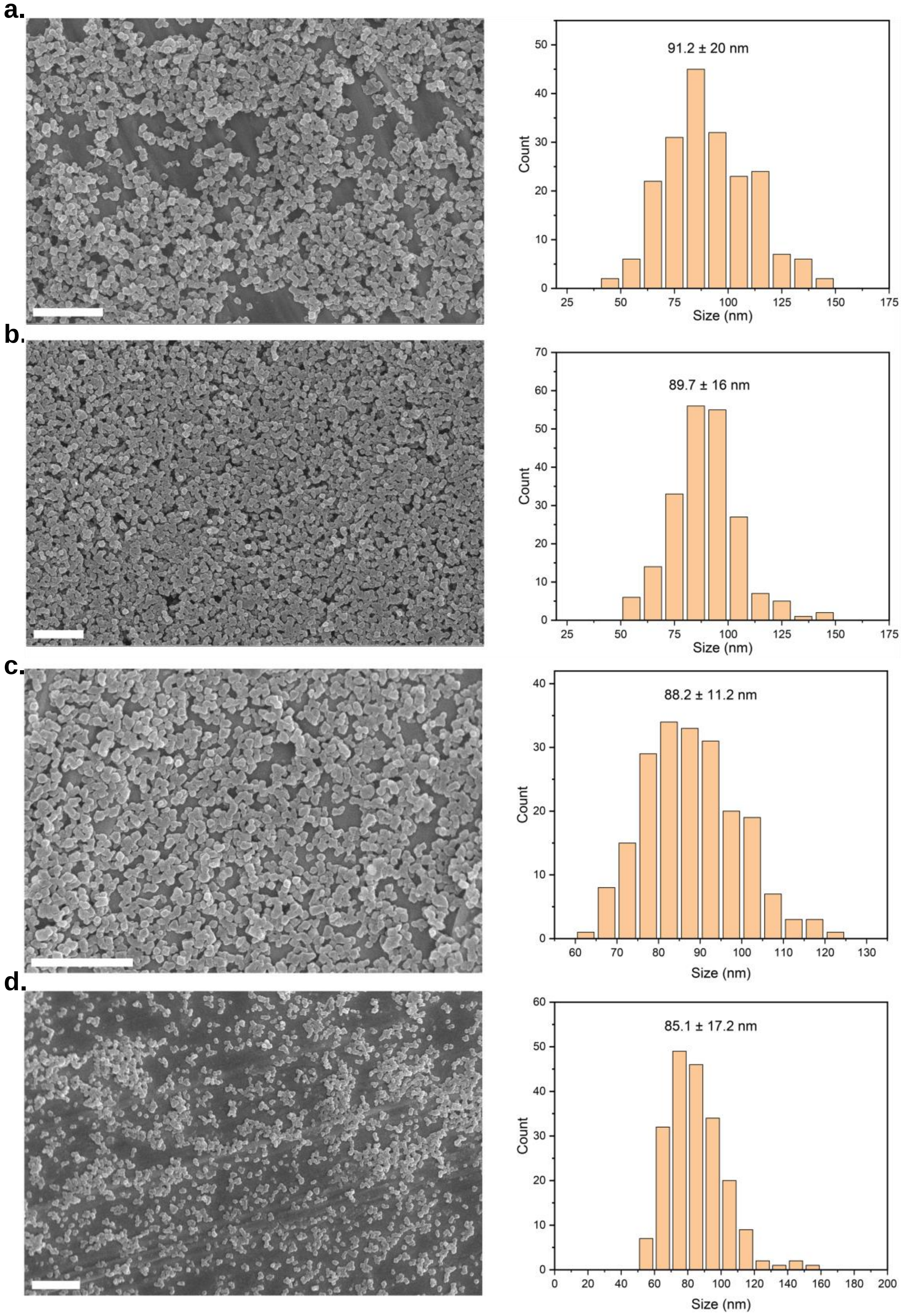

Figure S22. SEM images and particle size analysis of **a.** 69-Me_2, **b.** 69-L_2, **c.** 69-L_2@P and **d.** 69-L_2@F. Average particle sizes were determined by manually counting at least 200 particles. Scale bar, 1 µm.

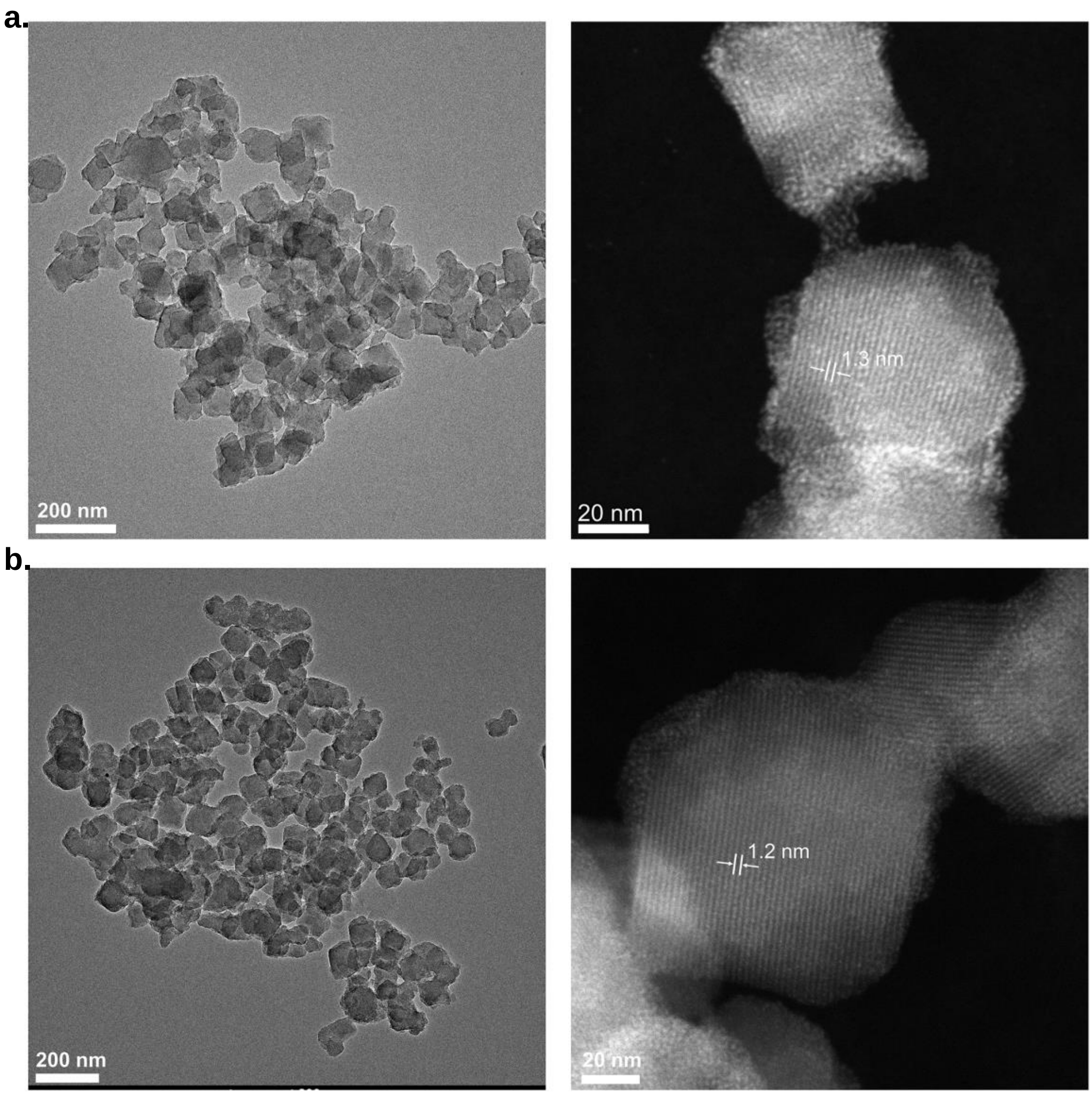

Figure S23. TEM (left) and HAADF-STEM (right) images of **a.** 69-Me_2 and **b.** 69-L_2

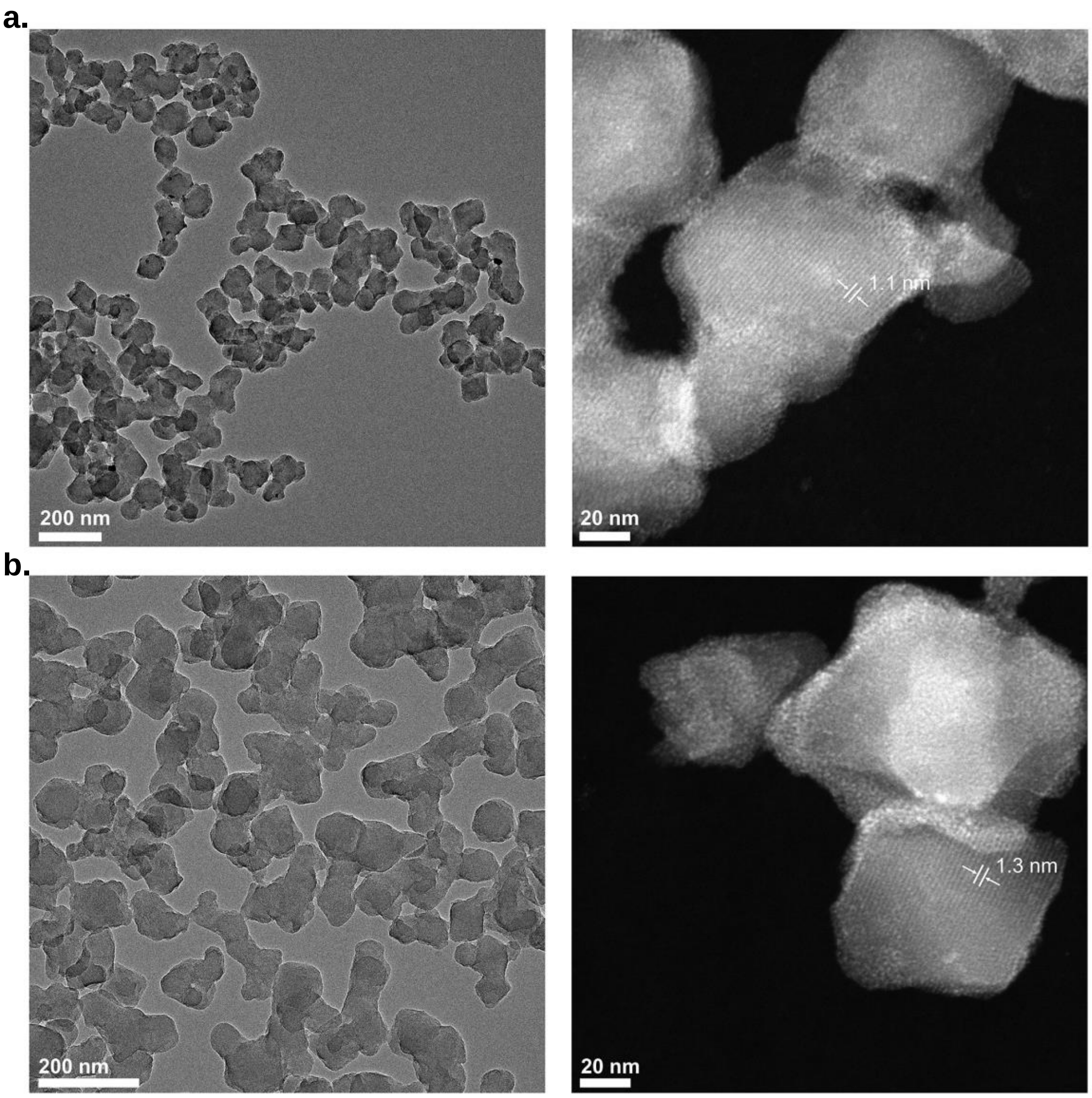

Figure S24. TEM (left) and HAADF-STEM (right) images of **a.** 69-L_2@P and **b.** 69-L_2@F.

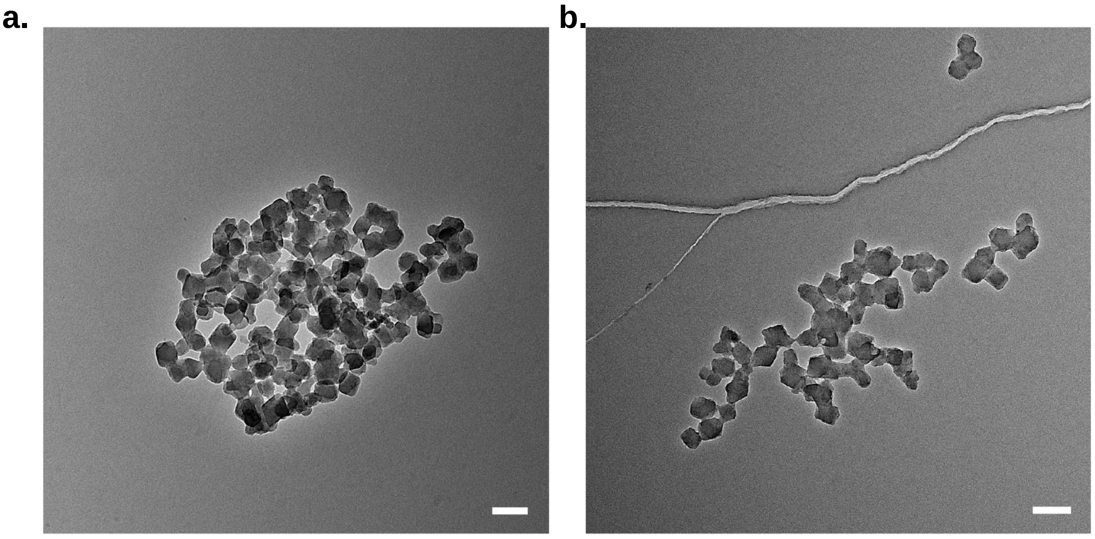

Figure S25. TEM images of **a.** 69-L_2 and **b.** 69-L_2@F in water after 3 weeks. Scale bar: 100 nm.

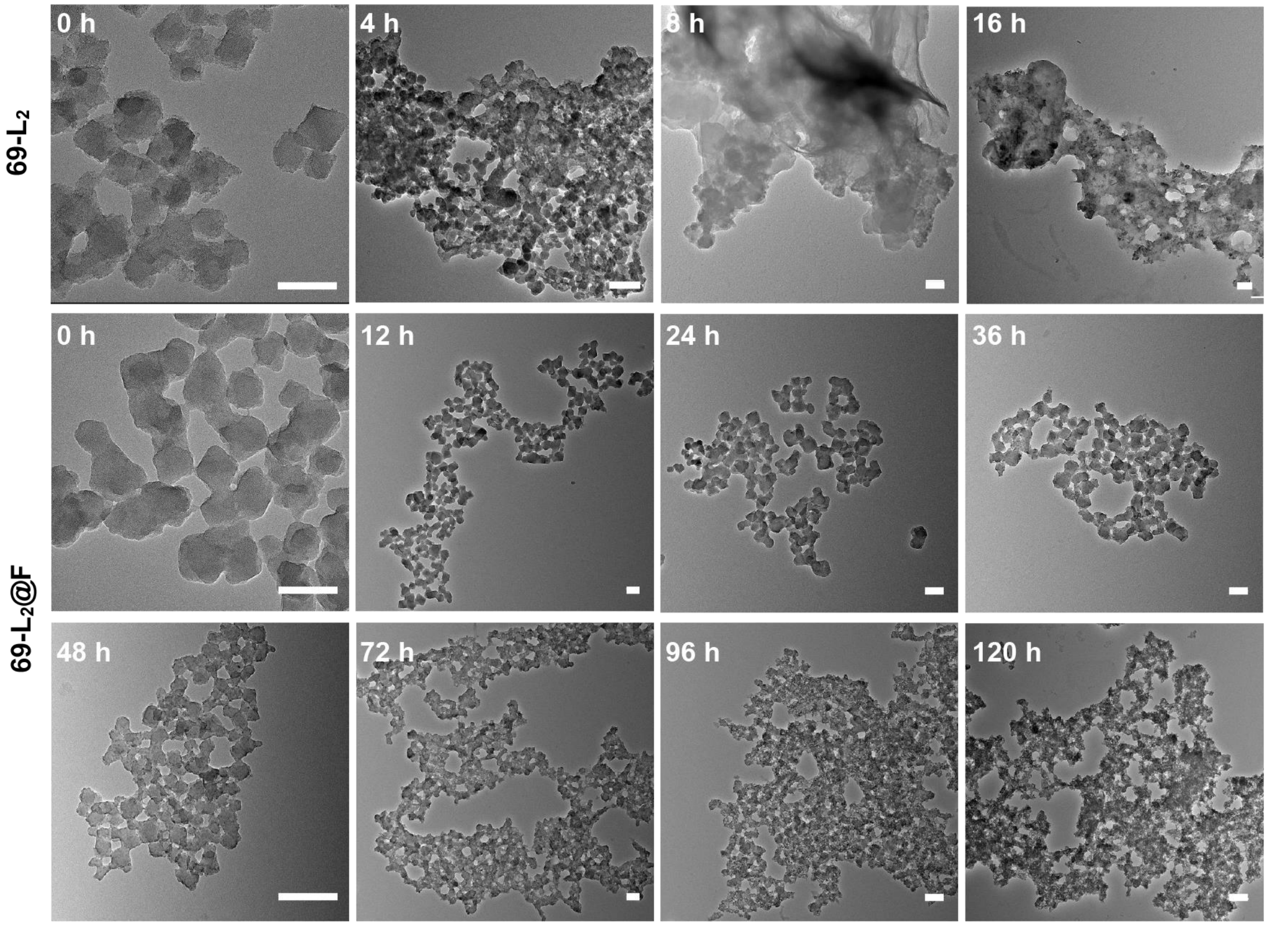

Figure S26. Time-dependent TEM bare 69-L_2 and 69-L_2@F in PBS (pH =7.4) monitored by TEM. Scale bar: 100 nm.

PXRD Patterns

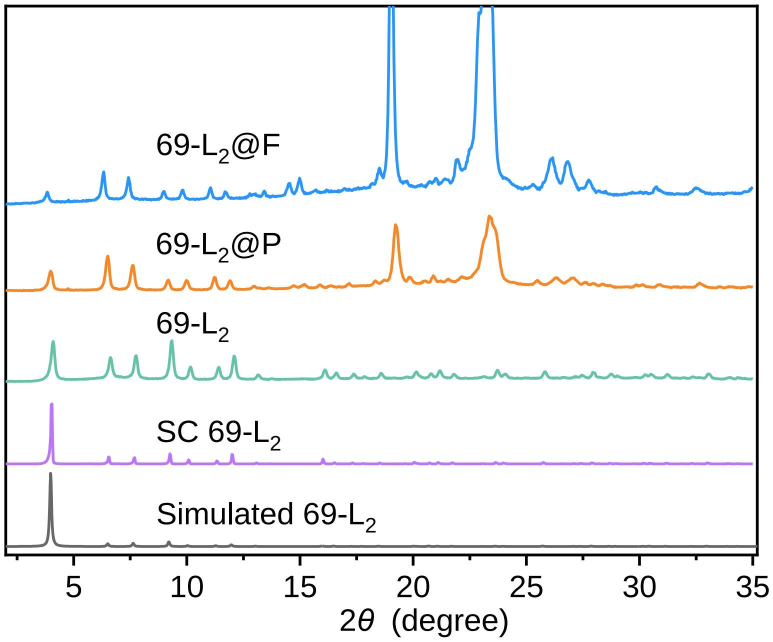

Figure S27. Simulated and experimental PXRD patterns.

FT-IR Spectra

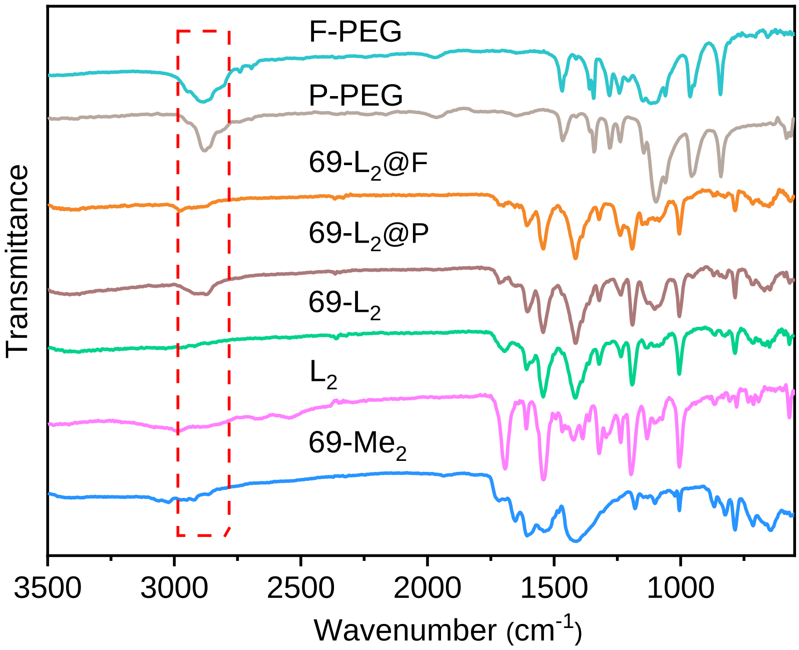

Figure S28. FT-IR spectra. The appearance of a new band at around 2880 cm^{-1} is attributed to the stretching vibration of C−H in the PEG chains,[10] indicating the existence of PEG in 69-L_2@P and 69-L_2@F.

TGA Curves

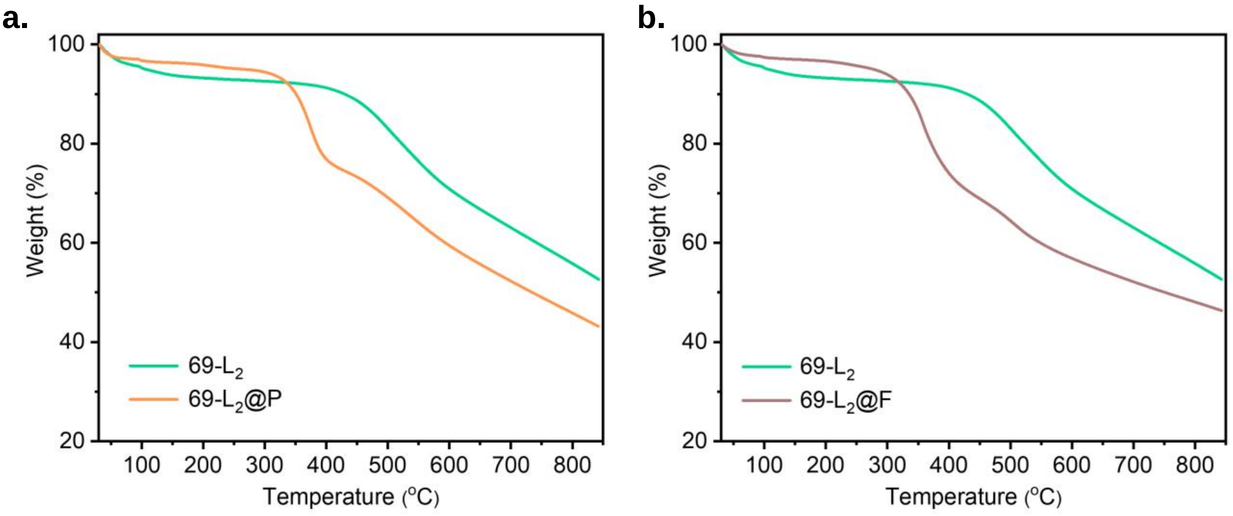

Figure S29. TGA profiles of **a.** 69-L_2 and 69-L_2@P, **b.** 69-L_2 and 69-L_2@P The encapsulated amount of P-PEG and F-PEG were calculated based on the difference between the TGA curve of the first plateau (376-452 °C).[11-12]

UV-Vis Spectra

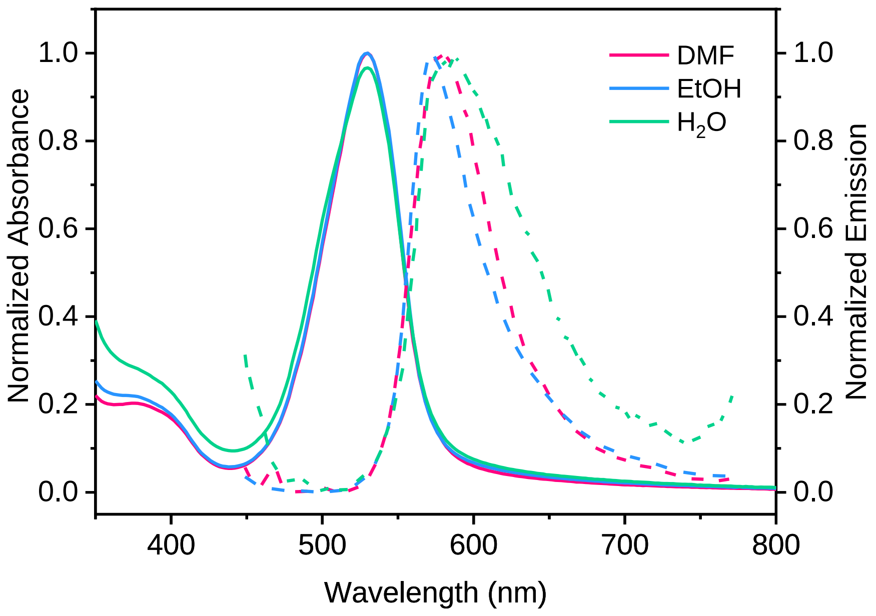

Figure S30. Normalized absorbance (solid line) and emission spectra (dashed line, λ_{ex} = 395 nm) of 69-L_2 in different solvents with varying polarities.

DLS

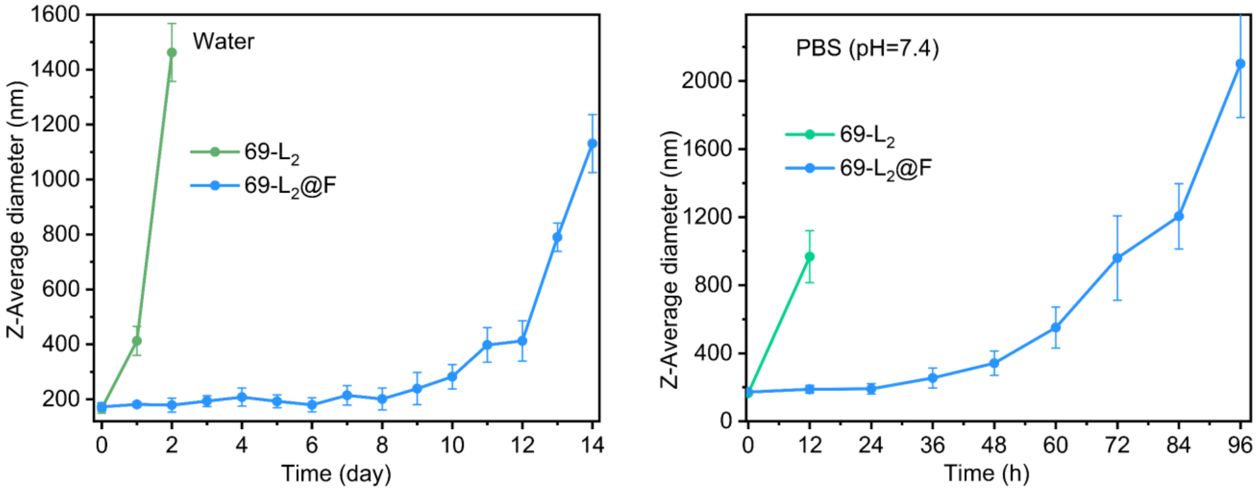

Figure S31. Long-term dispersity of 69-L_2 (green line) and 69-L_2@F (blue line) in water or PBS (pH = 7.4). The sizes were evaluated by DLS (n=3). During the period of measurement, the samples were stirred at room temperature

.

.

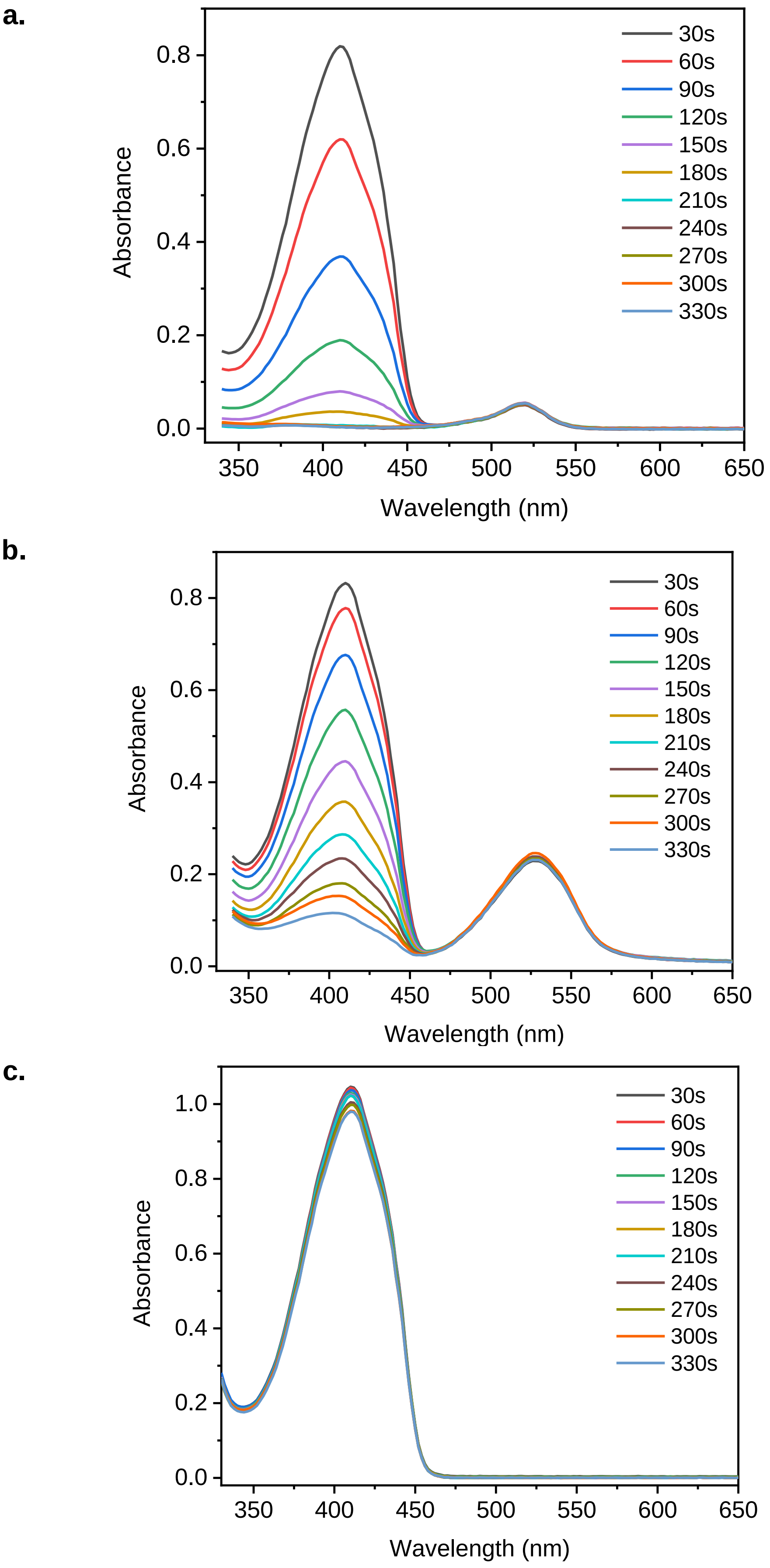

Figure S32. The detection of ROS generation using DPBF as $^{1}O_2$ indicator in water. **a,** 69-L_2@F. **b,** 69-L_2@P. **c,** DPBF. The absorbance at 410 nm of DPBF decreased gradually after reacting with $^{1}O_2$.

S4 *In Vitro* Study

Cytotoxicity In the Dark Conditions

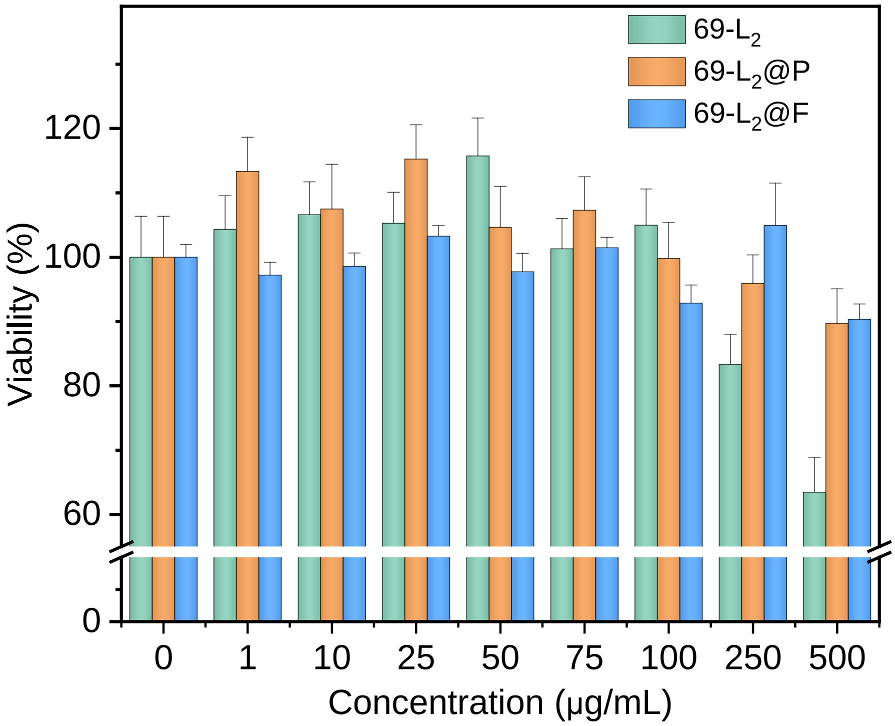

Figure S33. Cytotoxicity of 69-L_2, 69-L_2@P and 69-L_2@F under dark conditions. MDA-MB-231 cells' viability was measured by MTS assay after 72 h incubation.

TEM of the stained cells

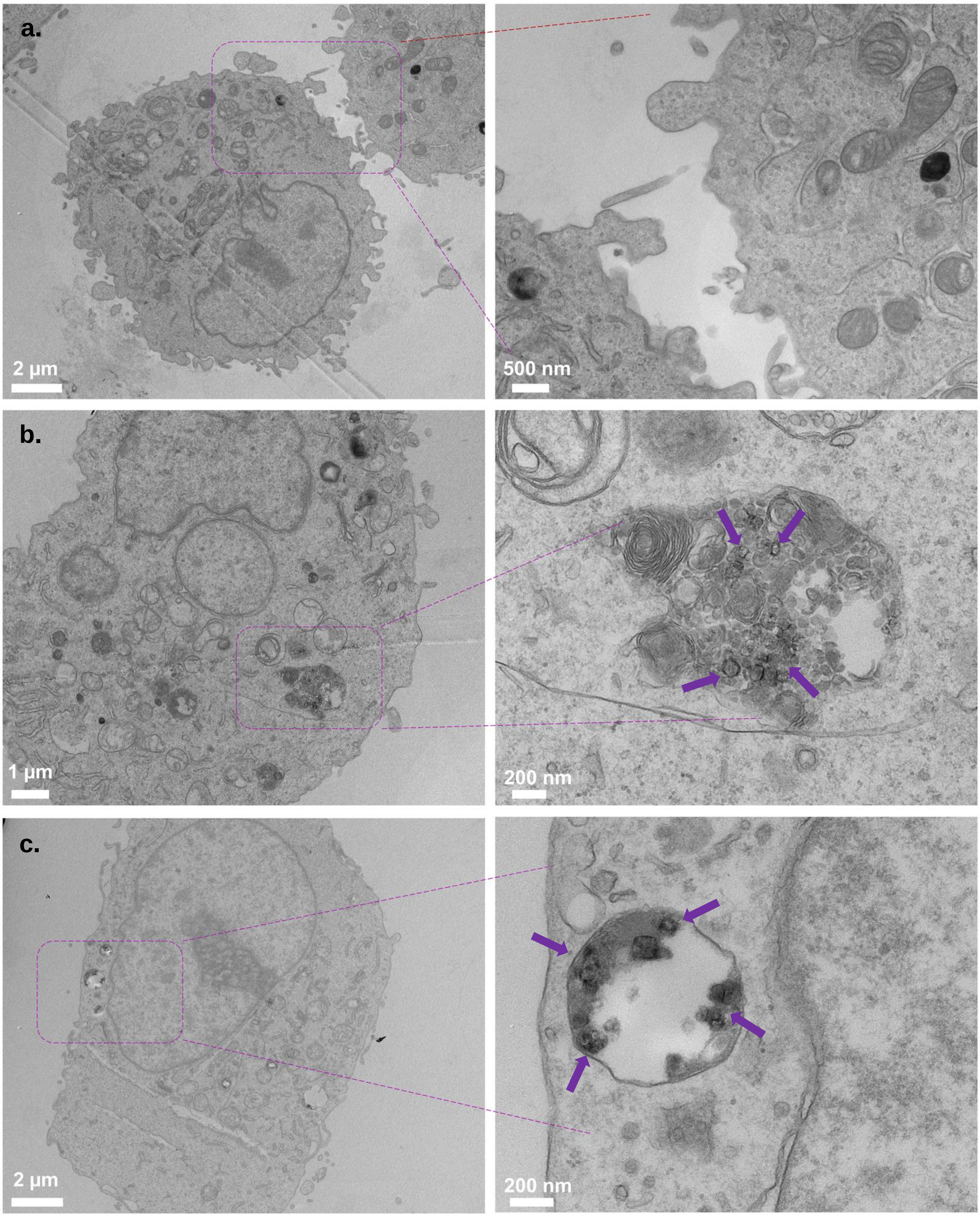

Figure S34. TEM images of the stained MDA-MB-231 cells after incubation with BODIPY-MOF samples. **a,** Untreated. **b,** 69-L_2@P. **c,** 69-L_2@F. Purple arrows denote MOFs nanoparticles.

Confocal Laser Scanning Microscopy (CLSM) Imaging

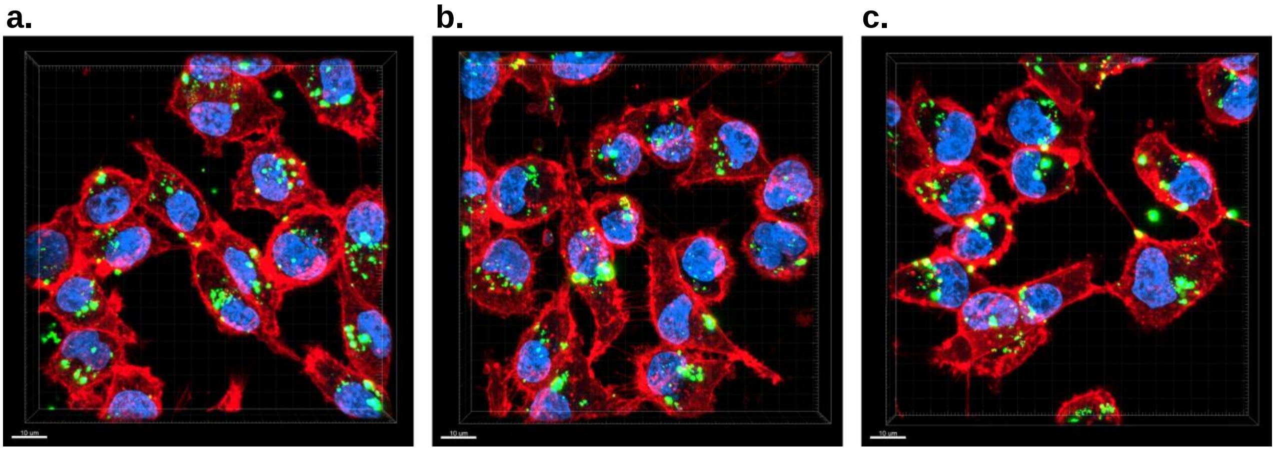

Figure S35. 3D z-stack CLSM of **a.** 69-L_2, **b,** 69-L_2@P and **c,** 68-L_2@F. Scale bar: 10 µm (Videos S1-S3).

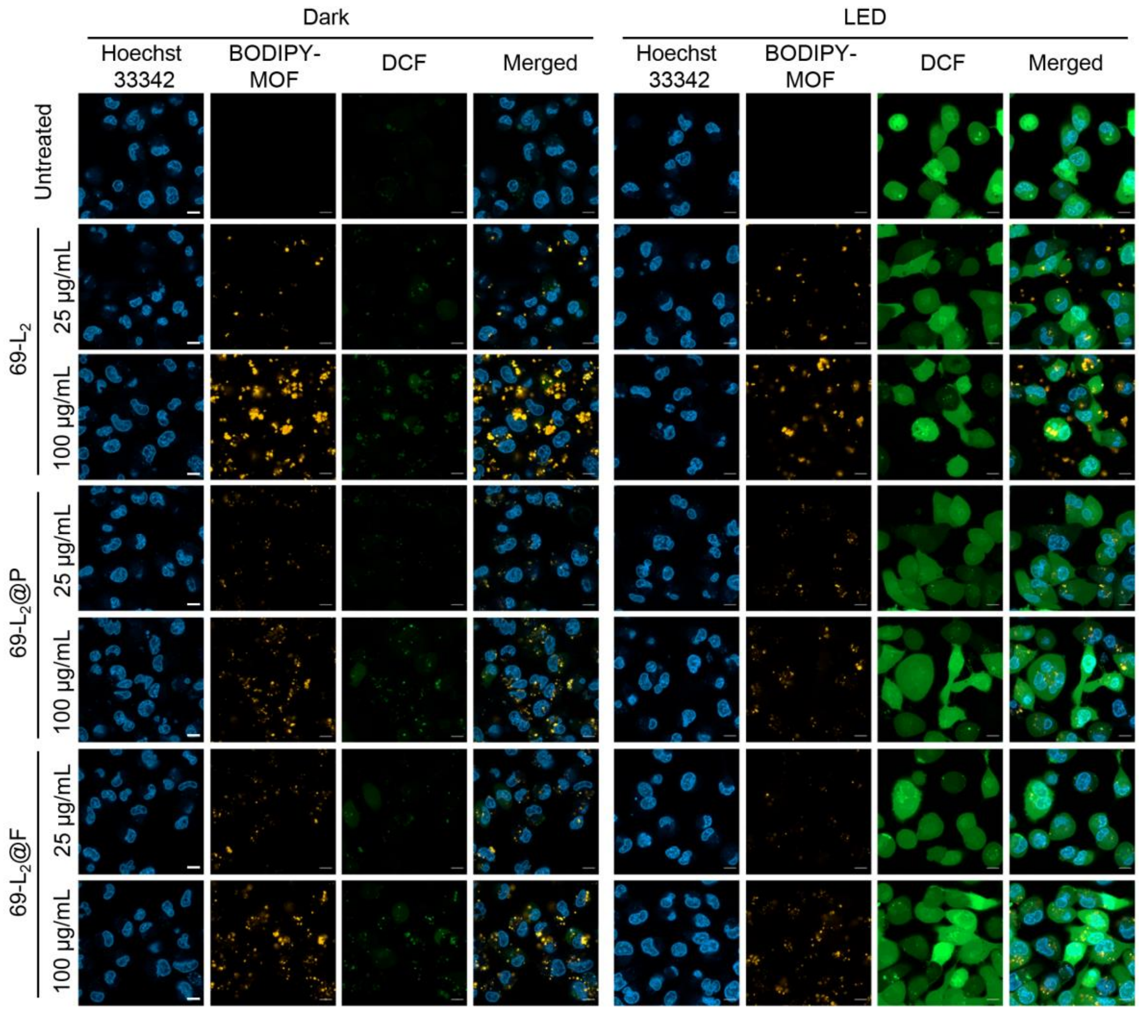

Figure S36. 2D CLSM observation of ROS generation of MDA-MB-231 cells incubated with 69-L_2, 69-L_2@P, and 69-L_2@F (25 µg/mL and 100 µg/mL based on the concentration of 69-L_2) under dark or LED light irradiation in normoxic conditions. When exposed to ROS, the nonfluorescent DCFH-DA could be efficiently converted into fluorescent DCF. The weak green emission in the untreated sample was due to intracellular oxidative stress. Scale bar: 10 µm.

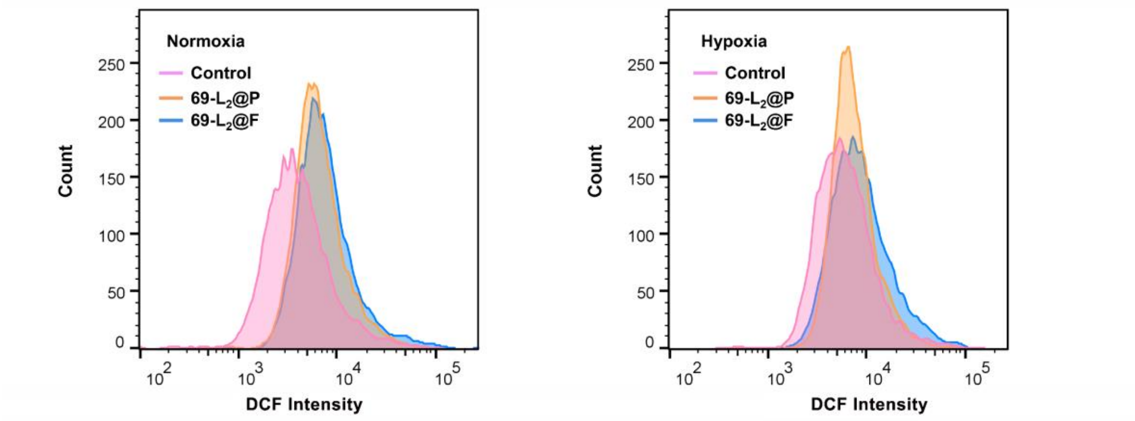

	Sample Name	Mean : Comp-FITC-A
	Control	6254
	69-L2@P	8478
	69-L2@F	10506

	Sample Name	Mean : Comp-FITC-A
	Control	8471
	69-L2@P	8903
	69-L2@F	11558

Figure S37. Overlapped histograms of DCF intensity for normoxic and hypoxic MDA-MB-231 cells incubated with 69-L_2@P and 69-L_2@F with light irradiation.

Live cell images of PDT treated MDA-MB-231 cells by IncuCyte

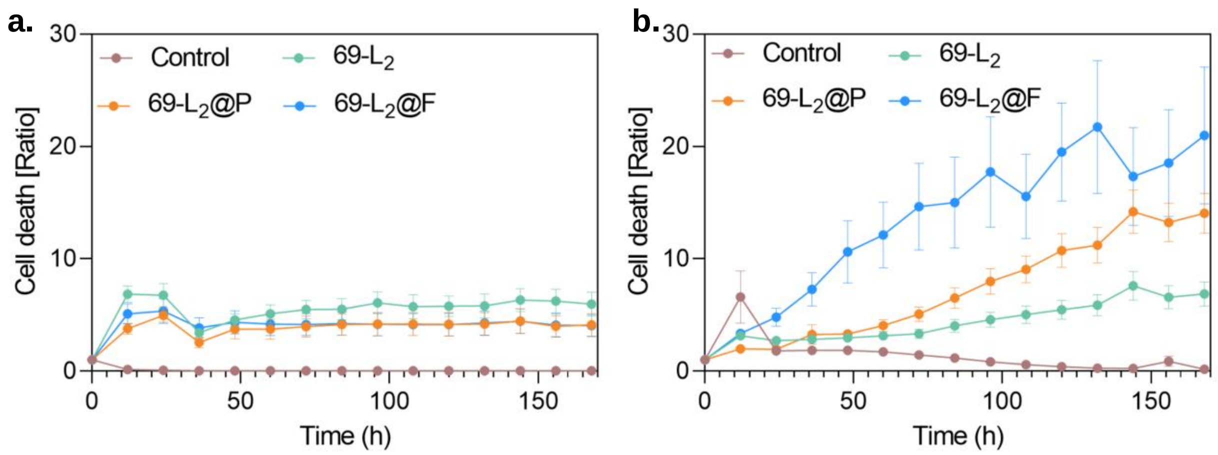

Figure S38. Relative cell death of MDA-MB-231 cells after incubation with the control, 69-L_2, 69-L_2@P or 69-L_2@F **a,** without, and **b,** with LED light irradiation.

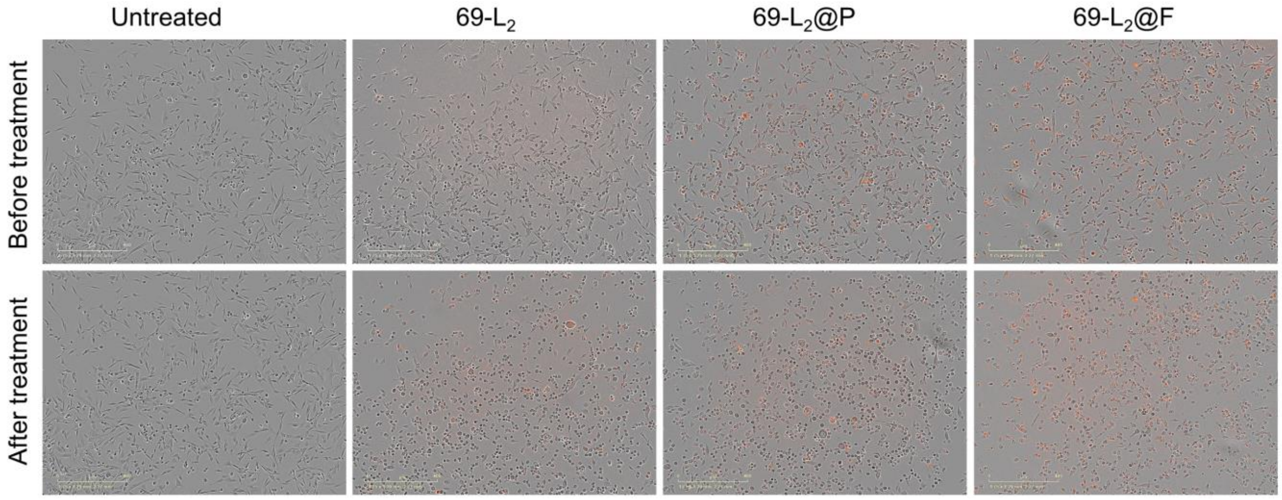

Figure S39. Live cell images of PDT treated MDA-MB-231 cells by IncuCyte before and after LED light treatment.

S5 *In Vivo* Study

***In vivo* tumor growth and PDT.** Athymic Balb/C female nude mice (5 weeks old, average weight = 20 g) were purchased from Charles River Laboratories (France) and acclimated for 1 week before the experiments, in aseptic conditions with water and standard pellet food provided *ad libitum* and with light/dark cycles of 12 h. For breast tumor induction, luciferase-expressing MDA-MB-231 cells ($5x10^6$/mouse) were injected subcutaneously on the right flank. The tumor volumes were measured every other day with a caliper and calculated by: tumor volume (mm^3) = width. ($length^2$)/2. When they reached approximately 30 mm^3, mice were distributed in four groups (5 mice/group): 1) Hydrogel, 2) 69-L_2@F-Hydrogel (69-L_2@F-Gel), 3) Hydrogel + LED, 4) 69-L_2@F-Hydrogel (69-L_2@F-Gel) + LED. After being anesthetized with isoflurane (IsoFlo 100%p/p), 50 µL per mouse of the hydrogel only or the 69-L_2@F-hydrogel were injected intratumorally (1.25% w/v HEC hydrogel or 2.5 mg/mL 69-L_2 in 1.25% w/v HEC hydrogel, respectively, in groups 1) and 3) or groups 2) and 4).). Mice from groups 3) and 4) were irradiated with a green LED (525 nm high-power LED, 3.1 W, SOLIS-525C, ThorLabs) for 10 minutes. After receiving the treatment, fluorescence imaging was performed on each group using the Newton FT500 imaging system (Vilber) after excitation at 540 nm. Following the fluorescence imaging, mice were injected subcutaneously with D-Luciferin (ABP Biosciences, 15 mg/mL in DPBS, 150 µL/mouse). After 10 minutes, the same system was used for bioluminescence imaging. The LED irradiation procedure was repeated on days 2 and 4 post intratumoral injection of the hydrogel or 69-L_2@F-Gel, and the fluorescence and bioluminescence imaging procedures were repeated on days 2, 4 and 7. At the endpoint (day 7), mice were euthanized by cervical dislocation. The tumors and the organs (liver, kidneys, spleen, heart, and lungs) were collected and imaged for fluorescence and bioluminescence and then fixed in 10% formalin for further histology studies. Histological sections of the tumors were stained with haematoxylin and eosin. The quantification was done with the Visiopharm Integrator System software (VIS; Visiopharm A/S, Hoersholm, Denmark) and a NanoZoomer-SQ Digital slide scanner (Hamamatsu Photonics). For the quantitative measurements, on each tumor, a systematic uniform random sampling (meander sampling) was carried out for each slide. Step-lengths of 1134.70 µm were used in both x-and y-directions, enabling the acquisition of 80% of the total area using an objective of 10x. The meander sampling generated an average of 50 fields for each tumor, which were overlapped using a test system. A total of 25 grid points were regularly arranged, covering 41201.51 µm2 per point (area per point; a/p). The sectional area of the tumor and of the necrosis were estimated by an unbiased, stereological technique based on point-counting (Howard and Reed, 2004), in which the total number of grid points in a section hitting the structures of interest (p structure) was calculated: Sectional area per structure = Σ (p structure) * (a/p) * 2. For immunohistochemical analysis, the tumors were stained with the antibody anti-Ki67. A representative picture at 40x per section was taken and the immuneratio was calculated using the immunoratio plugin on FIJI. Areas of necrosis were avoided. All the animal experiments were approved by the Ethical Committee and the Animal Welfare and

Ethics Body of the Nova Medical School (21_01_ORBEA.1) and followed the Animal Research guidelines of Nova Medical School and of the Directorate-General for Food and Veterinary Medicine.

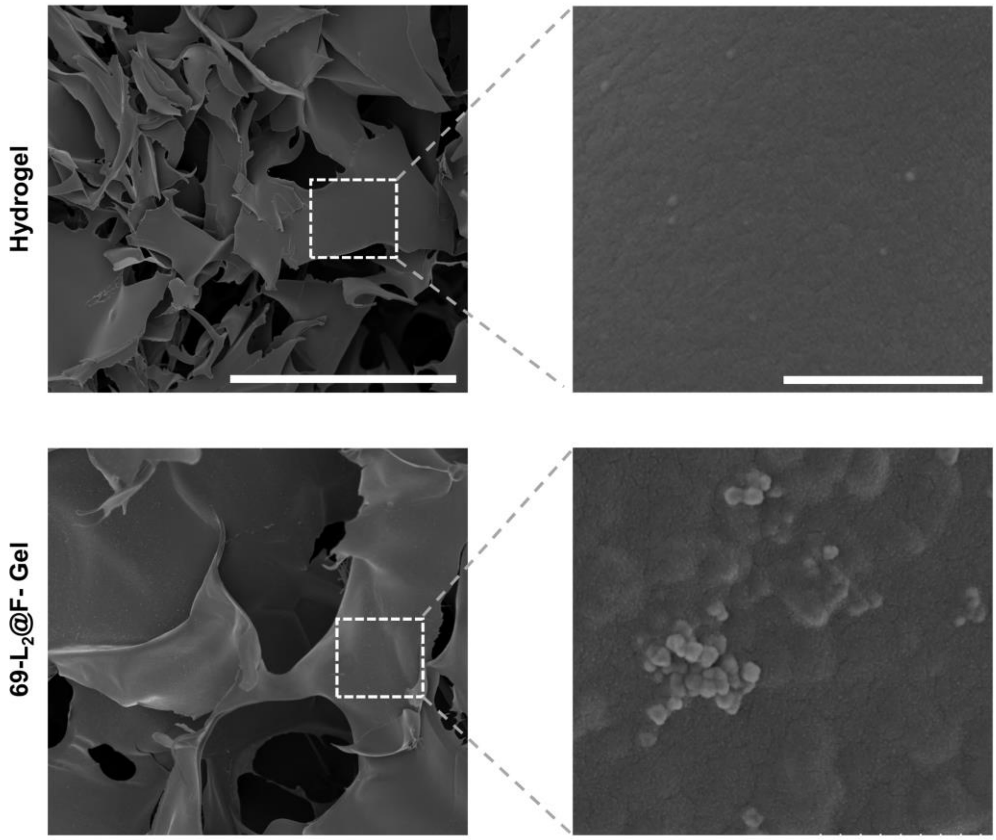

Figure S40. SEM imaging of only hydrogel (upper image) and 69-L_2@F-Gel (lower image). Scale bar: 100 µm (inset: 1 µm).

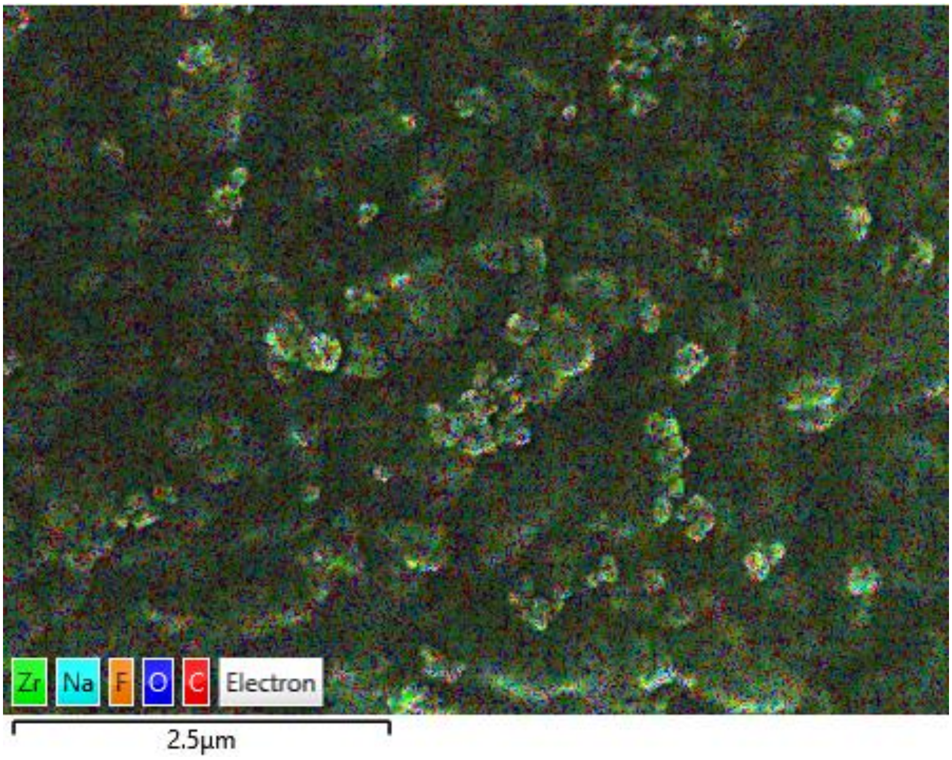

Figure S41. Energy-dispersive X-ray spectroscopy (EDS) mapping of 69-L_2@F-Gel confirms that 69-L_2@F composite is homogeneously distributed through the hydrogel.

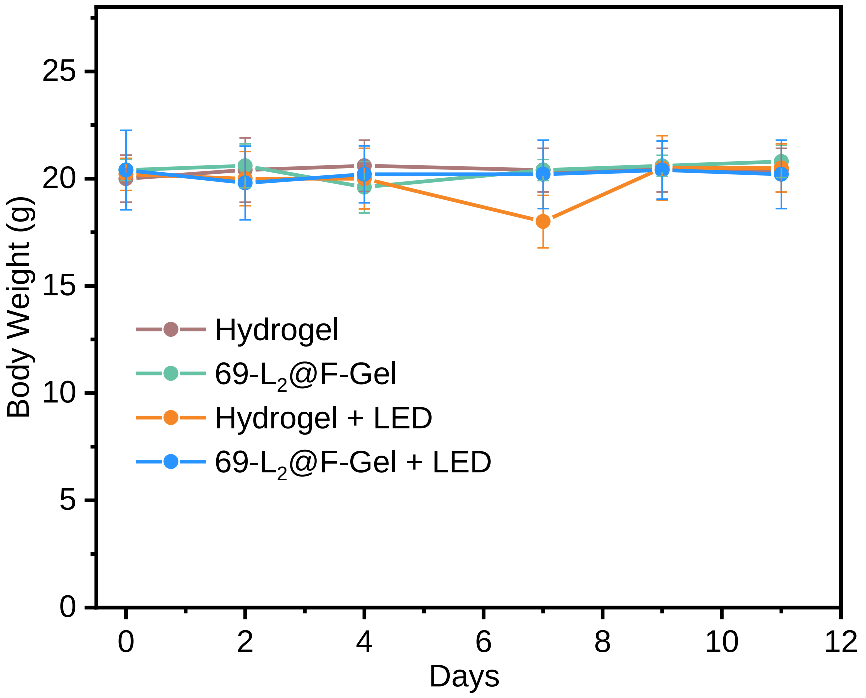

Figure S42. The safety of 69-L_2@F-Gel was confirmed by monitoring body weight. Body weight is depicted as the mean of each treatment group. No decrease or changes in body weight were found for all treatment mice groups (n= 5).

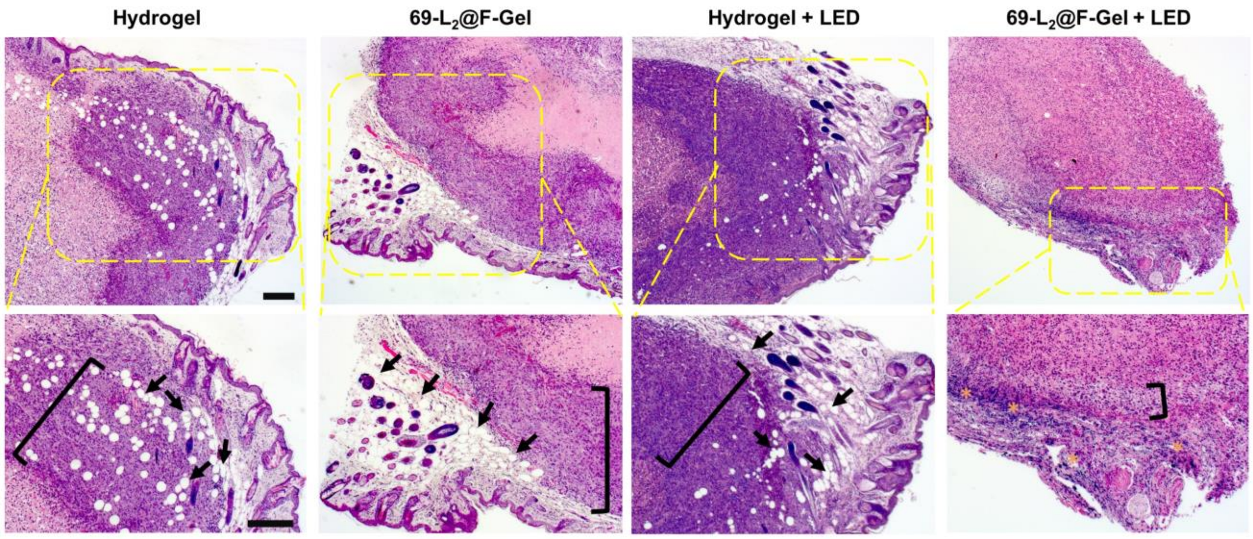

Figure S43. Haematoxylin and eosin (H&E) stains of tumors from treated groups with hydrogel or 69-L_2@F-Gel with or without green LED irradiation. Tumor front (Bracket), Adipocytes (Arrow), Necrotic tissue (Asterisk). Scale bar: 200 µm

S6. BET Areas Calculation Using BETSI.[13]

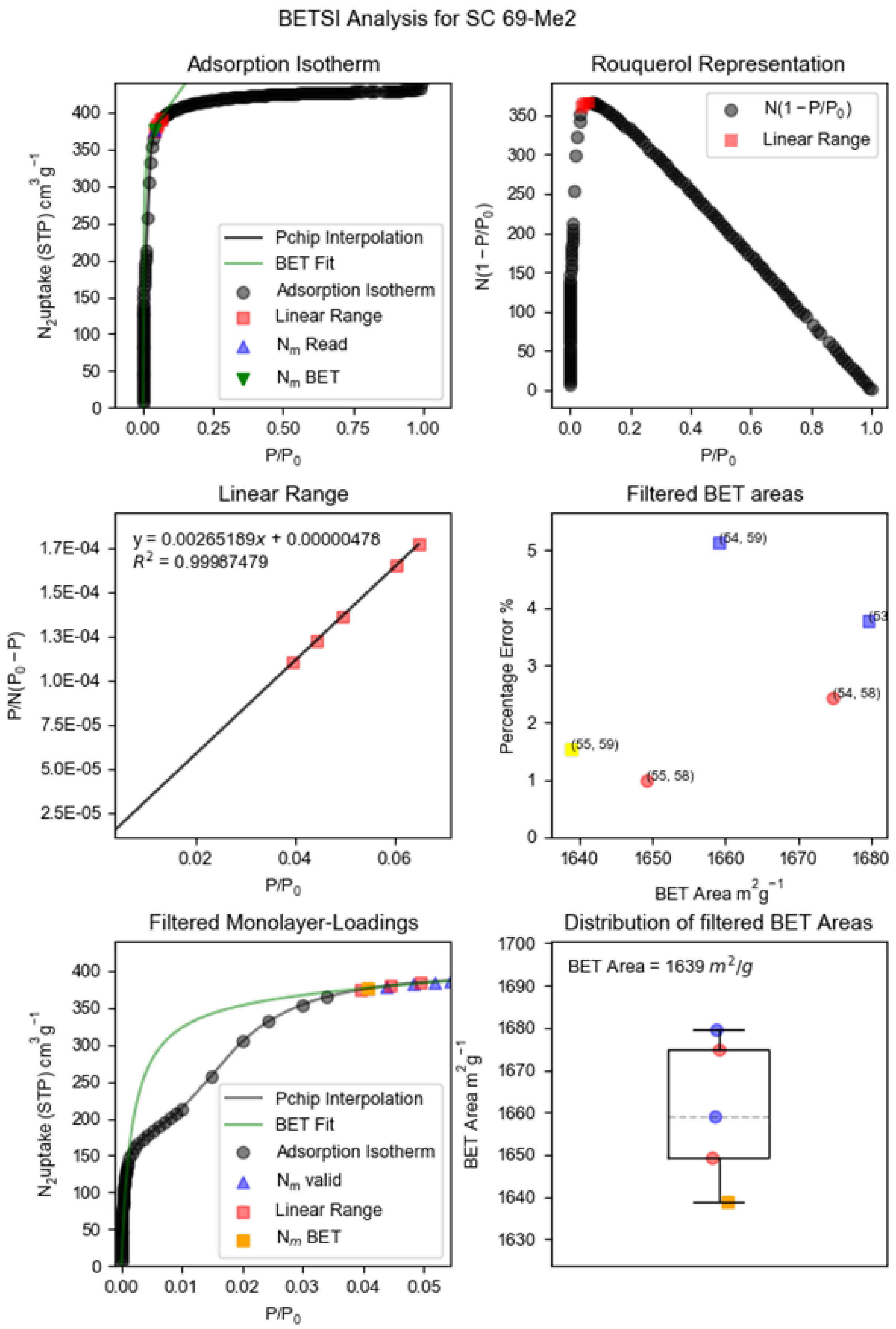

BETSI Regression Diagnostics for SC 69-Me2

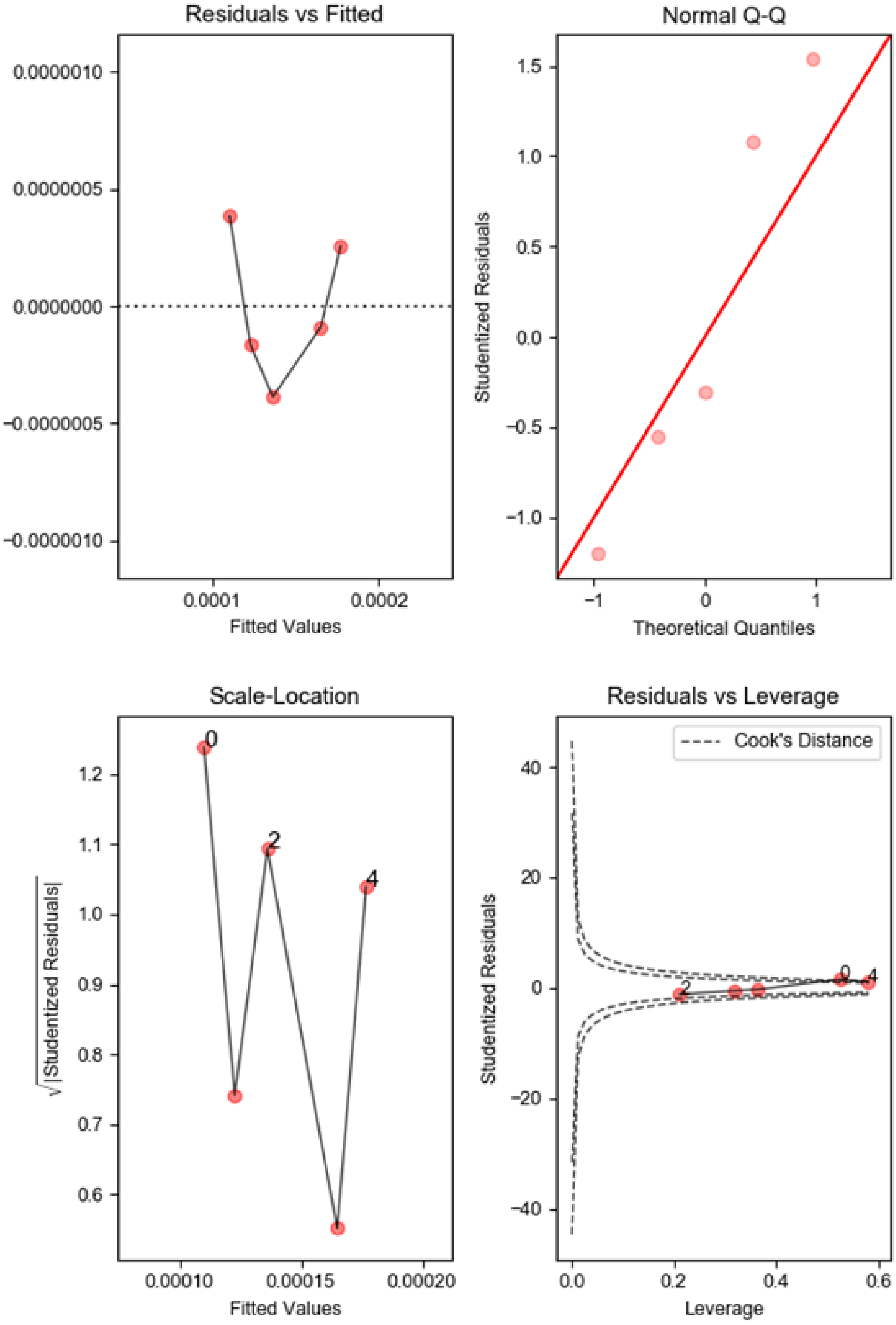

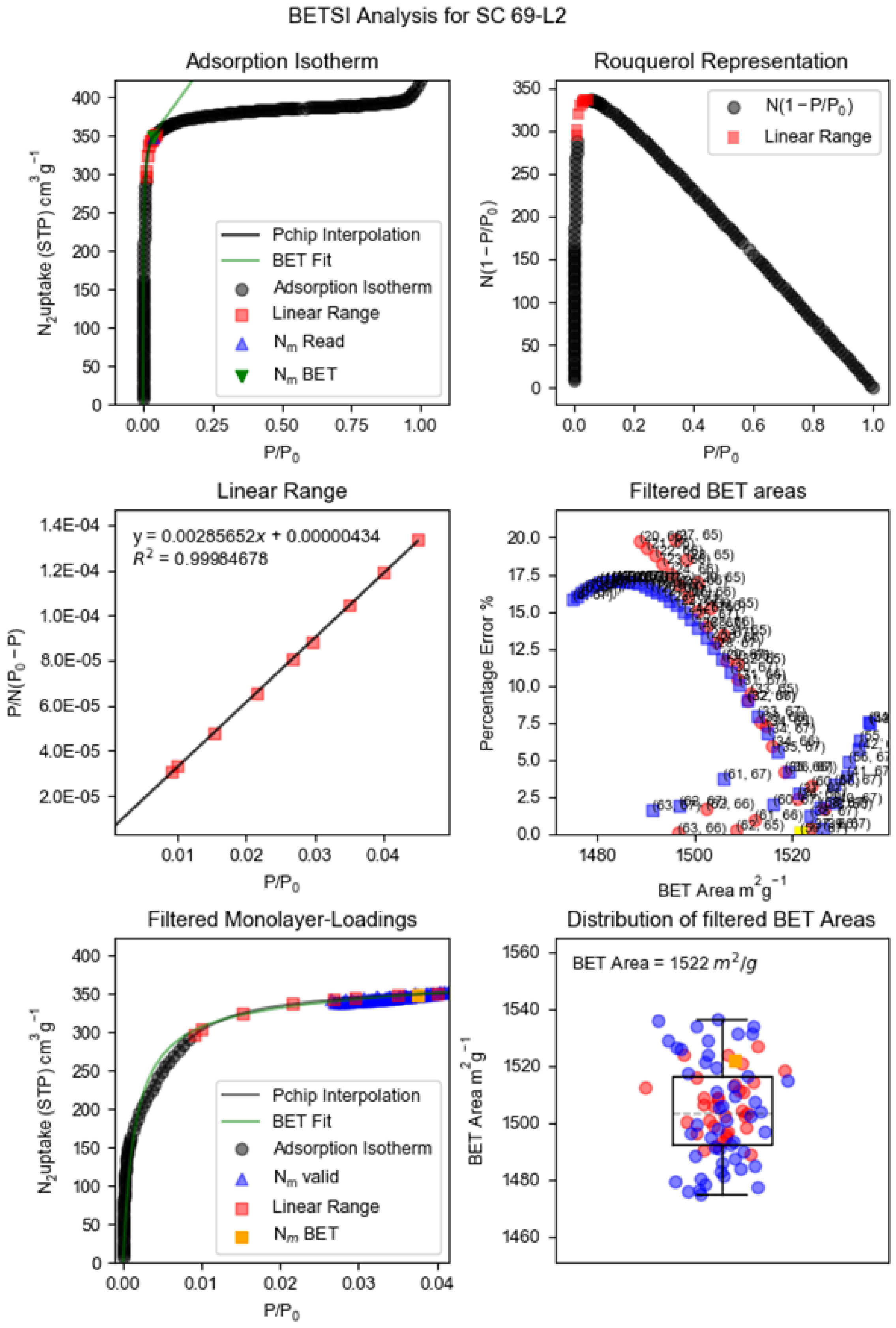
BETSI Analysis for SC 69-L2
Adsorption Isotherm
N2uptake (STP) cm3g-1
P/P0
Pchip Interpolation
BET Fit
Adsorption Isotherm
Linear Range
Nm Read
Nm BET
Rouquerol Representation
N(1 - P/P0)
Linear Range
Linear Range
P/N(P0 - P)
y = 0.00285652x + 0.00000434
R2 = 0.99984678
Filtered BET areas
Percentage Error %
BET Area m2g-1
Filtered Monolayer-Loadings
Nm valid
Nm BET
Distribution of filtered BET Areas
BET Area = 1522 m2/g
BET Area m2g-1

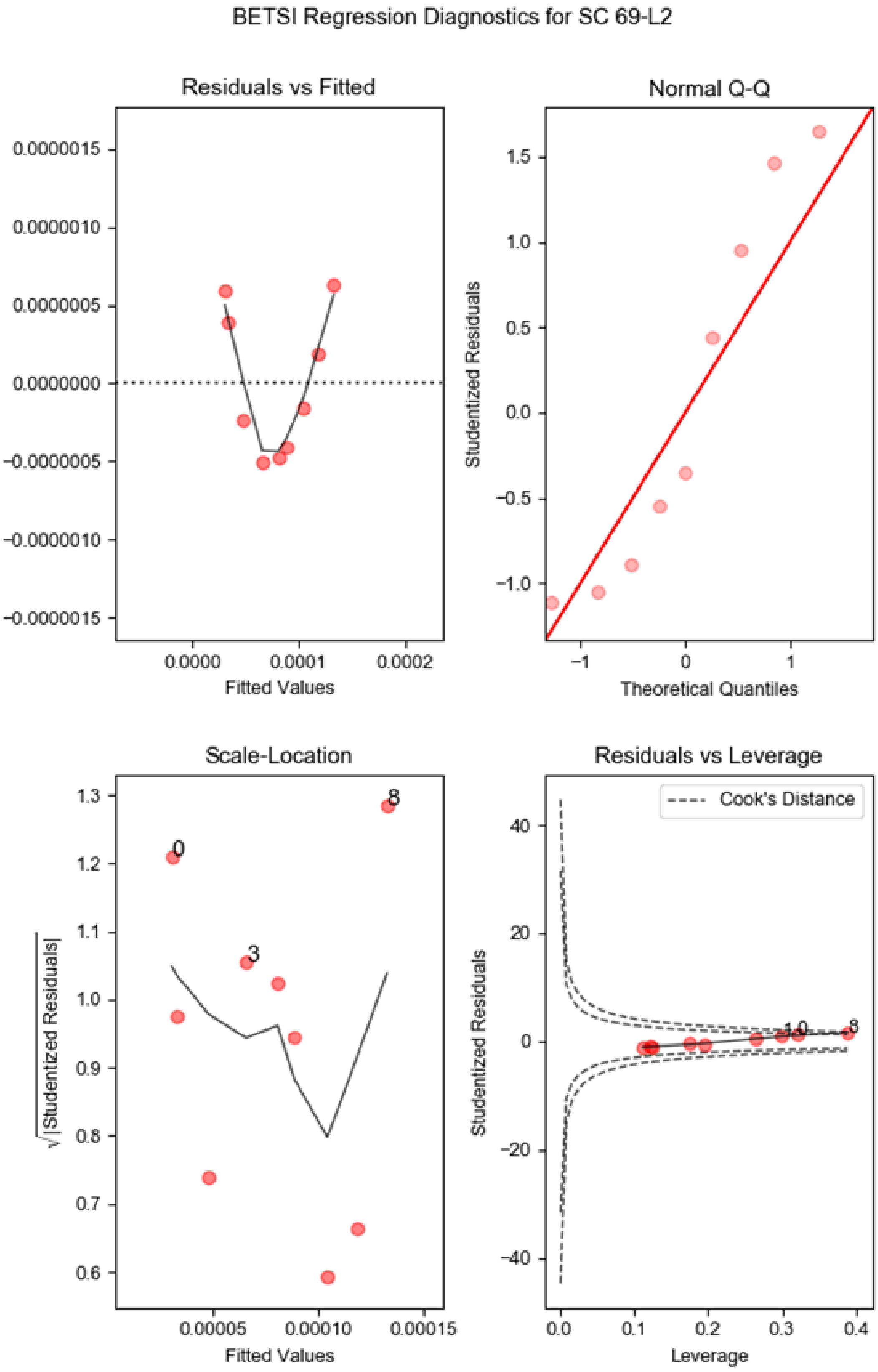
BETSI Regression Diagnostics for SC 69-L2
Residuals vs Fitted
0.0000015
0.0000010
0.0000005
0.0000000
−0.0000005
−0.0000010
−0.0000015
0.0000
0.0001
0.0002
Fitted Values
Normal Q-Q
Studentized Residuals
1.5
1.0
0.5
0.0
−0.5
−1.0
−1
0
1
Theoretical Quantiles
Scale-Location
√|Studentized Residuals|
1.3
1.2
1.1
1.0
0.9
0.8
0.7
0.6
0
3
8
0.00005
0.00010
0.00015
Fitted Values
Residuals vs Leverage
Cook's Distance
Studentized Residuals
40
20
0
−20
−40
8
0.0
0.1
0.2
0.3
0.4
Leverage

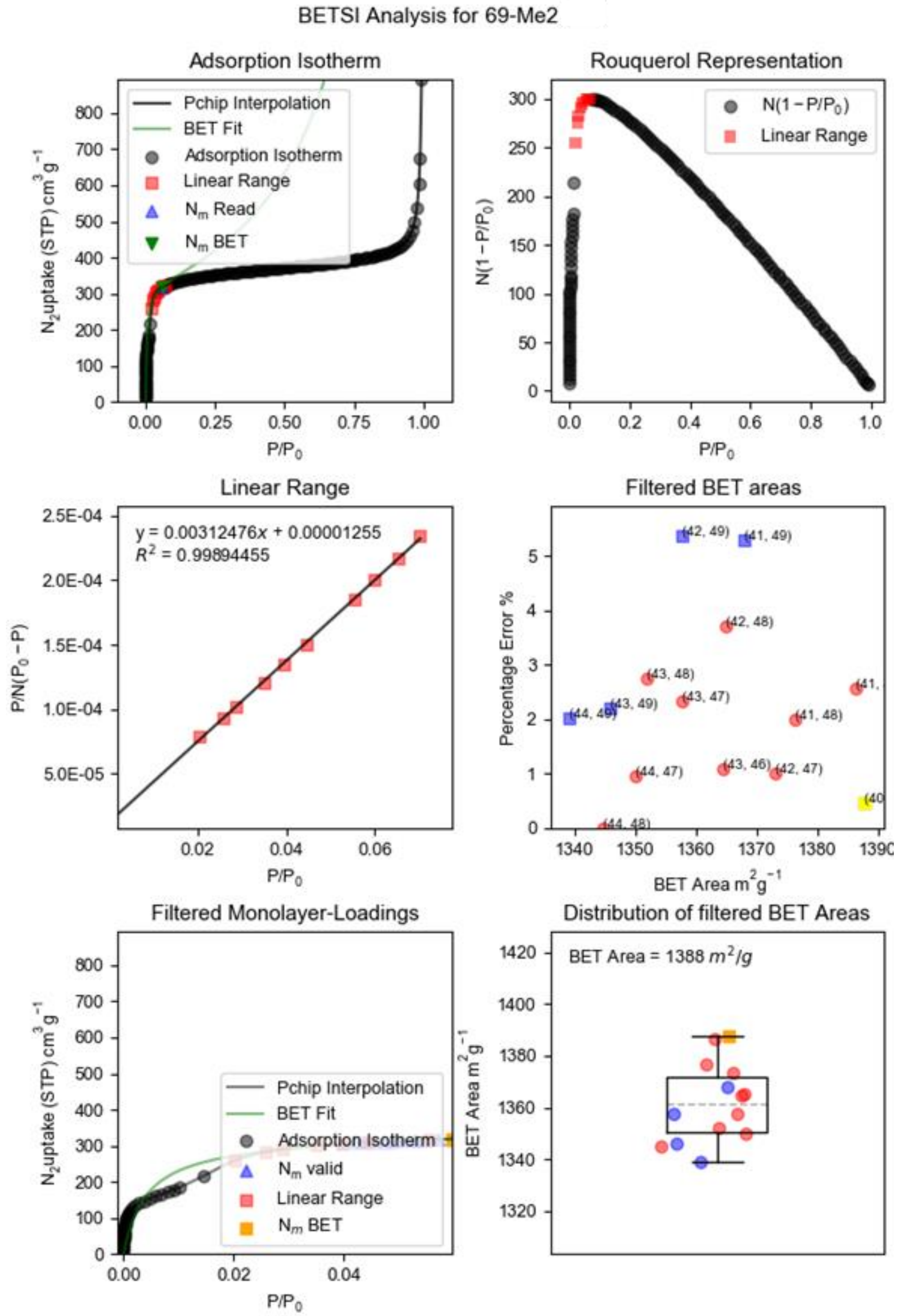
BETSI Analysis for 69-Me2
Adsorption Isotherm
Pchip Interpolation
BET Fit
Adsorption Isotherm
Linear Range
N_m Read
N_m BET
N_2 uptake (STP) cm^3 g^-1
P/P_0
Rouquerol Representation
N(1−P/P_0)
Linear Range
Linear Range
y = 0.00312476x + 0.00001255
R^2 = 0.99894455
P/N(P_0 −P)
Filtered BET areas
Percentage Error %
BET Area m^2 g^-1
Filtered Monolayer-Loadings
N_m valid
Distribution of filtered BET Areas
BET Area = 1388 m^2/g

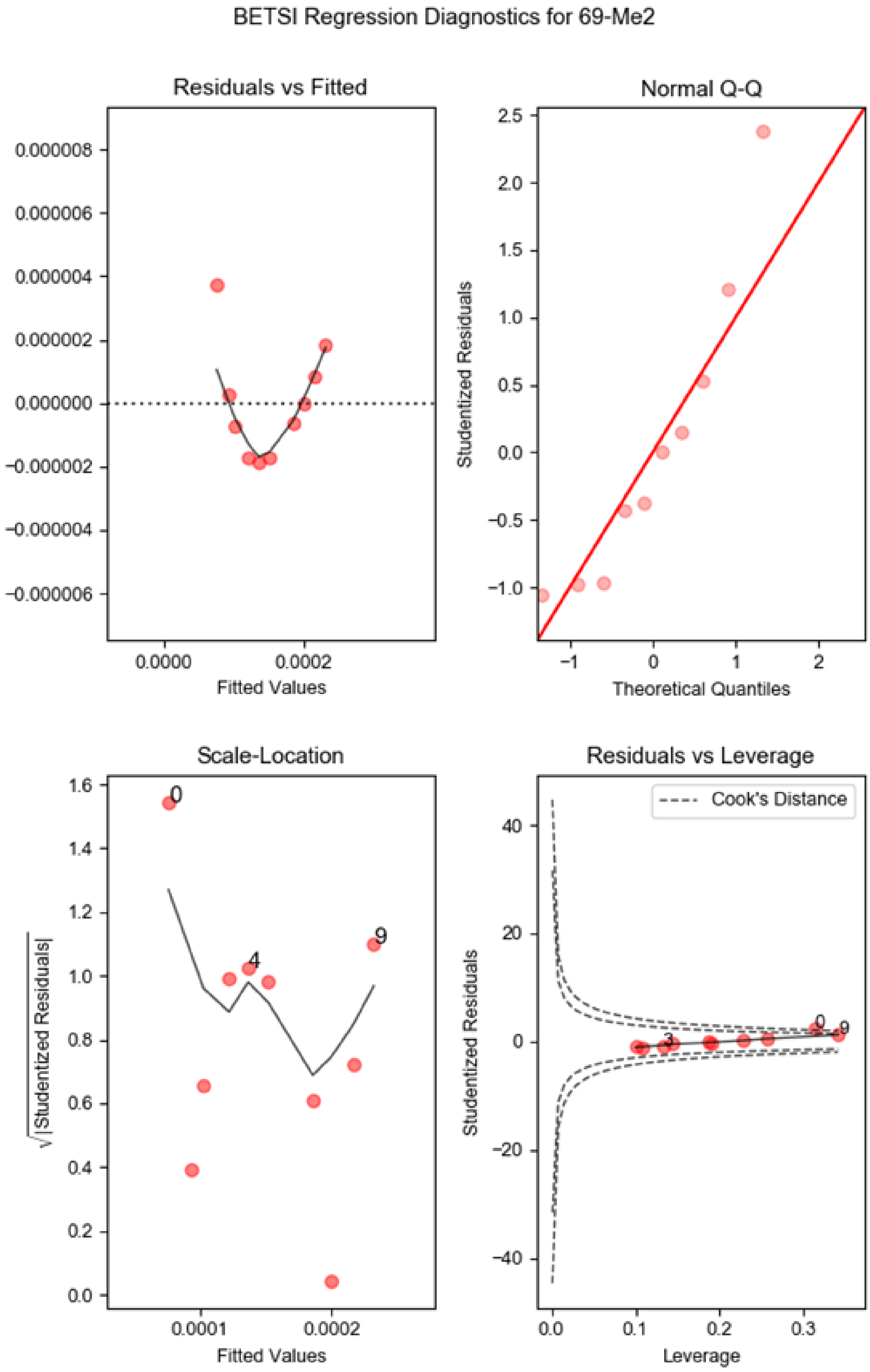
BETSI Regression Diagnostics for 69-Me2
Residuals vs Fitted
0.000008
0.000006
0.000004
0.000002
0.000000
−0.000002
−0.000004
−0.000006
0.0000
0.0002
Fitted Values
Normal Q-Q
2.5
2.0
1.5
1.0
0.5
0.0
−0.5
−1.0
−1
0
1
2
Studentized Residuals
Theoretical Quantiles
Scale-Location
1.6
1.4
1.2
1.0
0.8
0.6
0.4
0.2
0.0
0
4
9
√|Studentized Residuals|
0.0001
0.0002
Fitted Values
Residuals vs Leverage
Cook's Distance
40
20
0
−20
−40
0
3
9
Studentized Residuals
0.0
0.1
0.2
0.3
Leverage

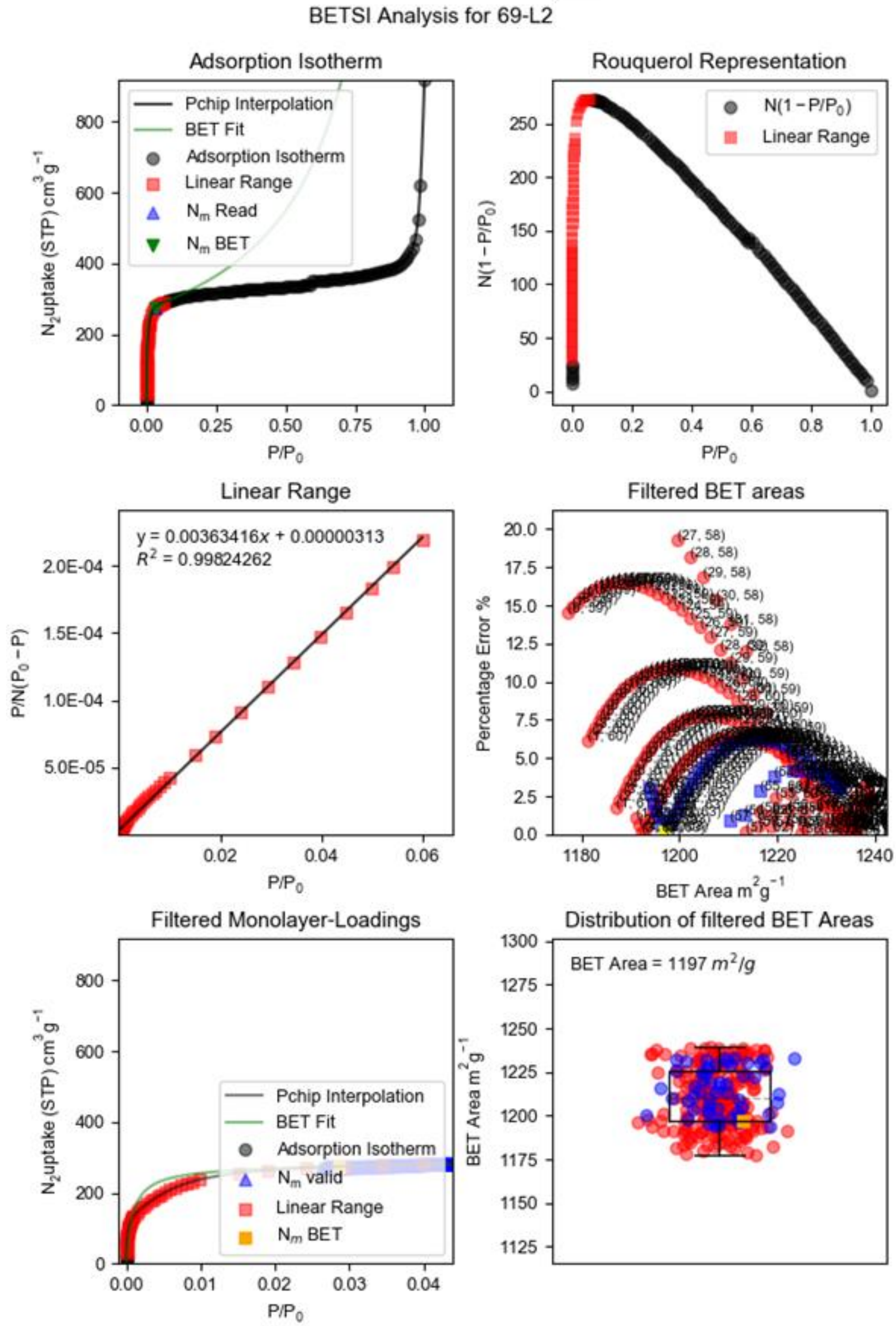
BETSI Analysis for 69-L2
Adsorption Isotherm
Pchip Interpolation
BET Fit
Adsorption Isotherm
Linear Range
Nm Read
Nm BET
N2 uptake (STP) cm3 g-1
P/P0
Rouquerol Representation
N(1 − P/P0)
Linear Range
Linear Range
y = 0.00363416x + 0.00000313
R2 = 0.99824262
P/N(P0 − P)
Filtered BET areas
Percentage Error %
BET Area m2 g-1
Filtered Monolayer-Loadings
Nm valid
Distribution of filtered BET Areas
BET Area = 1197 m2/g

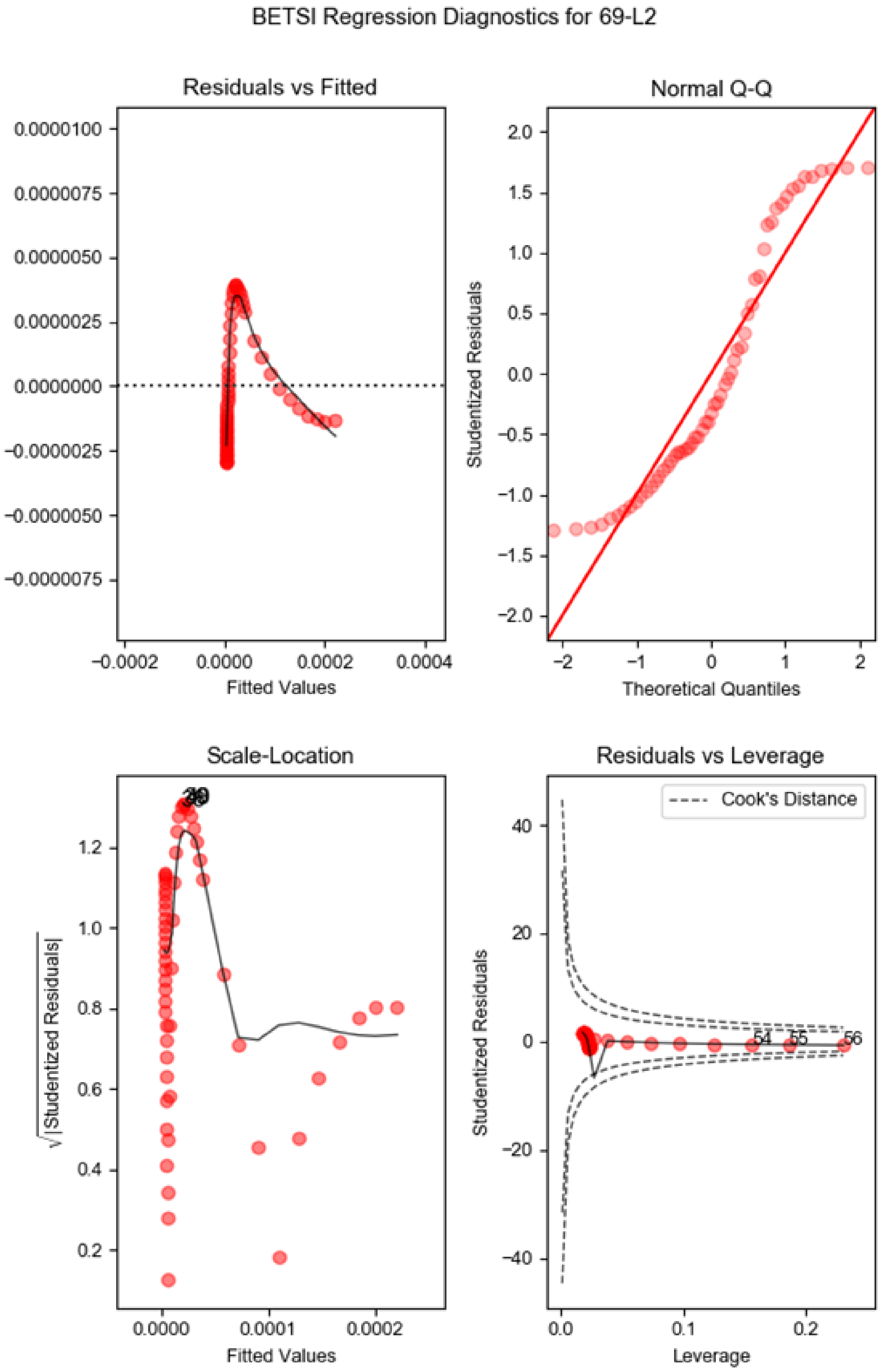
BETSI Regression Diagnostics for 69-L2
Residuals vs Fitted
Fitted Values
Normal Q-Q
Studentized Residuals
Theoretical Quantiles
Scale-Location
√|Studentized Residuals|
Fitted Values
Residuals vs Leverage
Cook's Distance
Studentized Residuals
Leverage
54
55
56

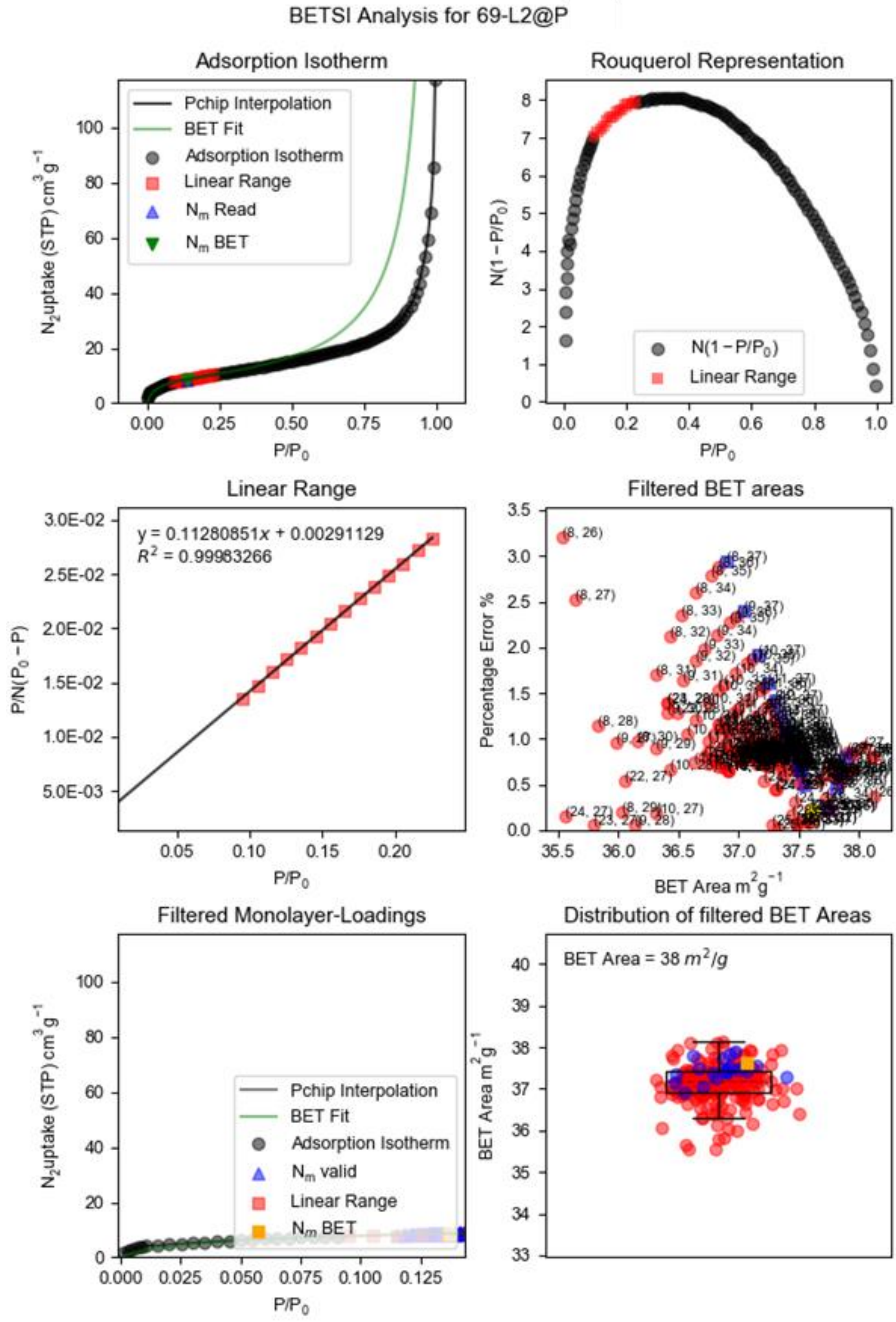

BETSI Analysis for 69-L2@P
Adsorption Isotherm
Pchip Interpolation
BET Fit
Adsorption Isotherm
Linear Range
N_m Read
N_m BET
N_2uptake (STP) cm^{3}g^{-1}
P/P$_0$
Rouquerol Representation
N(1 − P/P$_0$)
Linear Range
Linear Range
y = 0.11280851x + 0.00291129
R^2 = 0.99983266
P/N(P$_0$ − P)
Filtered BET areas
Percentage Error %
BET Area m^{2}g^{-1}
Filtered Monolayer-Loadings
N_m valid
Distribution of filtered BET Areas
BET Area = 38 m^2/g

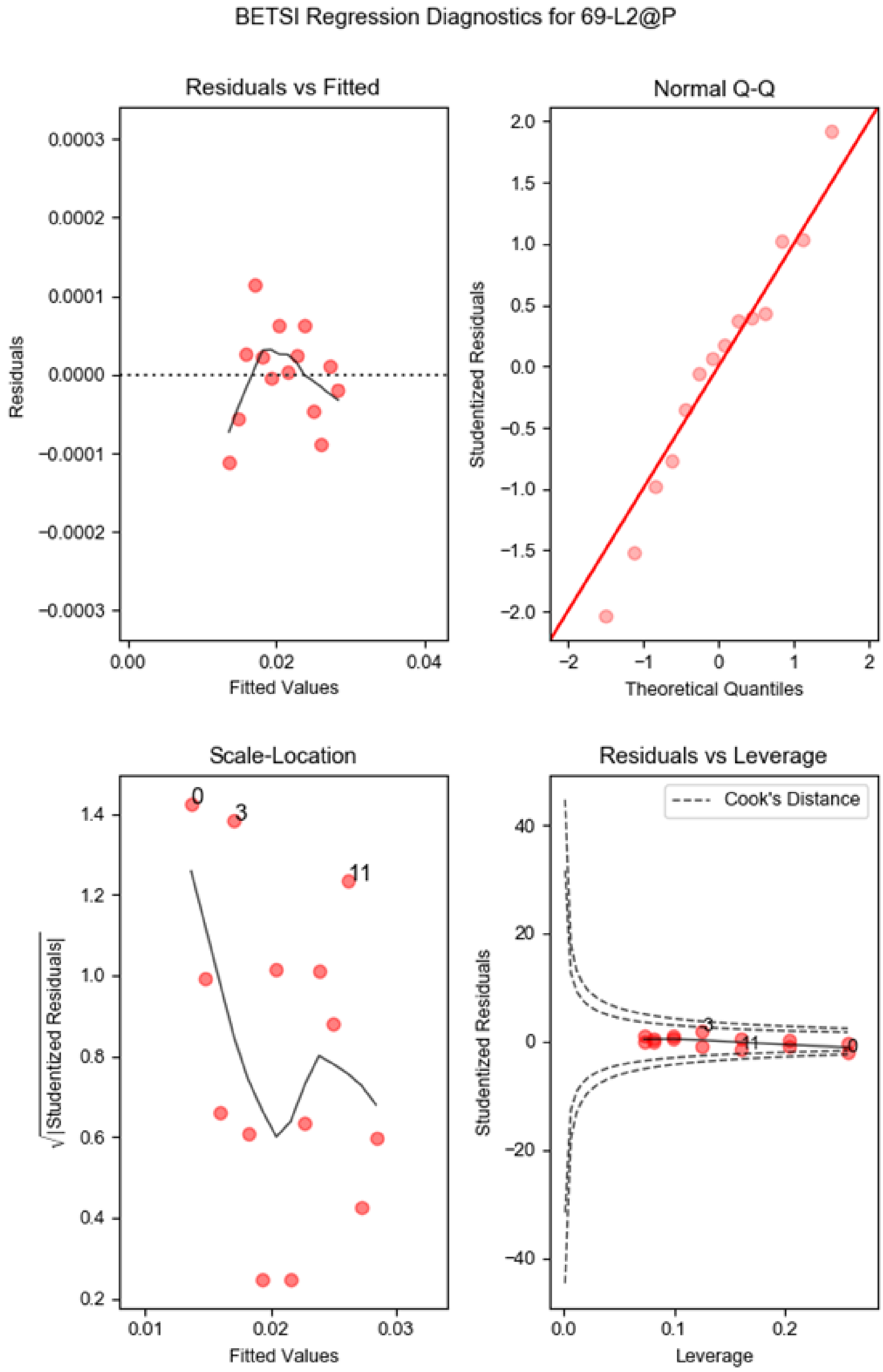
BETSI Regression Diagnostics for 69-L2@P
Residuals vs Fitted
Residuals
Fitted Values
0.0003
0.0002
0.0001
0.0000
−0.0001
−0.0002
−0.0003
0.00
0.02
0.04
Normal Q-Q
Studentized Residuals
Theoretical Quantiles
2.0
1.5
1.0
0.5
0.0
−0.5
−1.0
−1.5
−2.0
−2
−1
0
1
2
Scale-Location
√|Studentized Residuals|
Fitted Values
1.4
1.2
1.0
0.8
0.6
0.4
0.2
0.01
0.02
0.03
0
3
11
Residuals vs Leverage
Cook's Distance
Studentized Residuals
Leverage
40
20
0
−20
−40
0.0
0.1
0.2
3
11
0

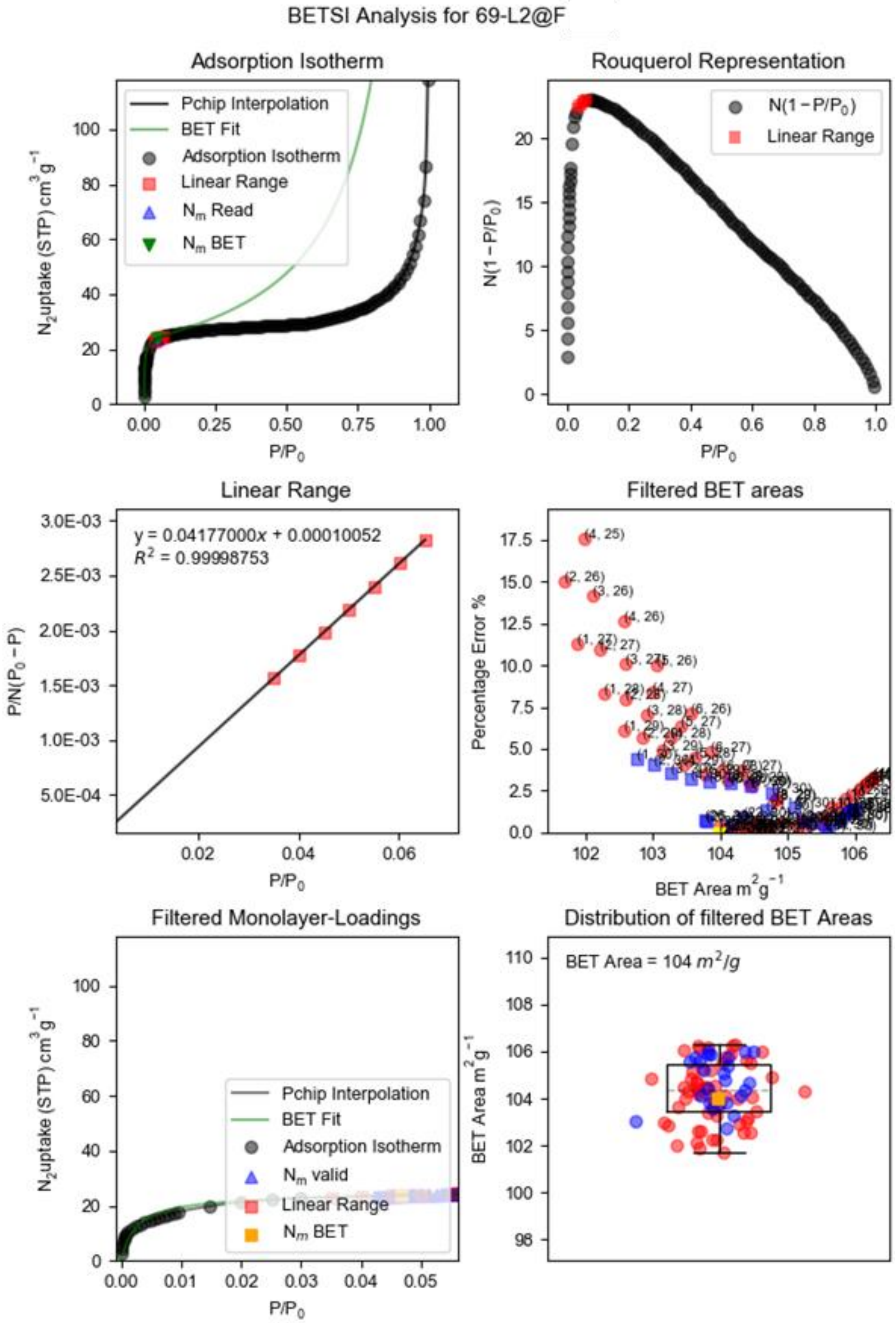

BETSI Analysis for 69-L2@F
Adsorption Isotherm
Pchip Interpolation
BET Fit
Adsorption Isotherm
Linear Range
N_m Read
N_m BET
N_2uptake (STP) $cm^3 g^{-1}$
P/P_0
Rouquerol Representation
$N(1-P/P_0)$
Linear Range
Linear Range
y = 0.04177000x + 0.00010052
R^2 = 0.99998753
$P/N(P_0-P)$
Filtered BET areas
Percentage Error %
BET Area $m^2 g^{-1}$
Filtered Monolayer-Loadings
Pchip Interpolation
BET Fit
Adsorption Isotherm
N_m valid
Linear Range
N_m BET
Distribution of filtered BET Areas
BET Area = 104 m^2/g
BET Area $m^2 g^{-1}$

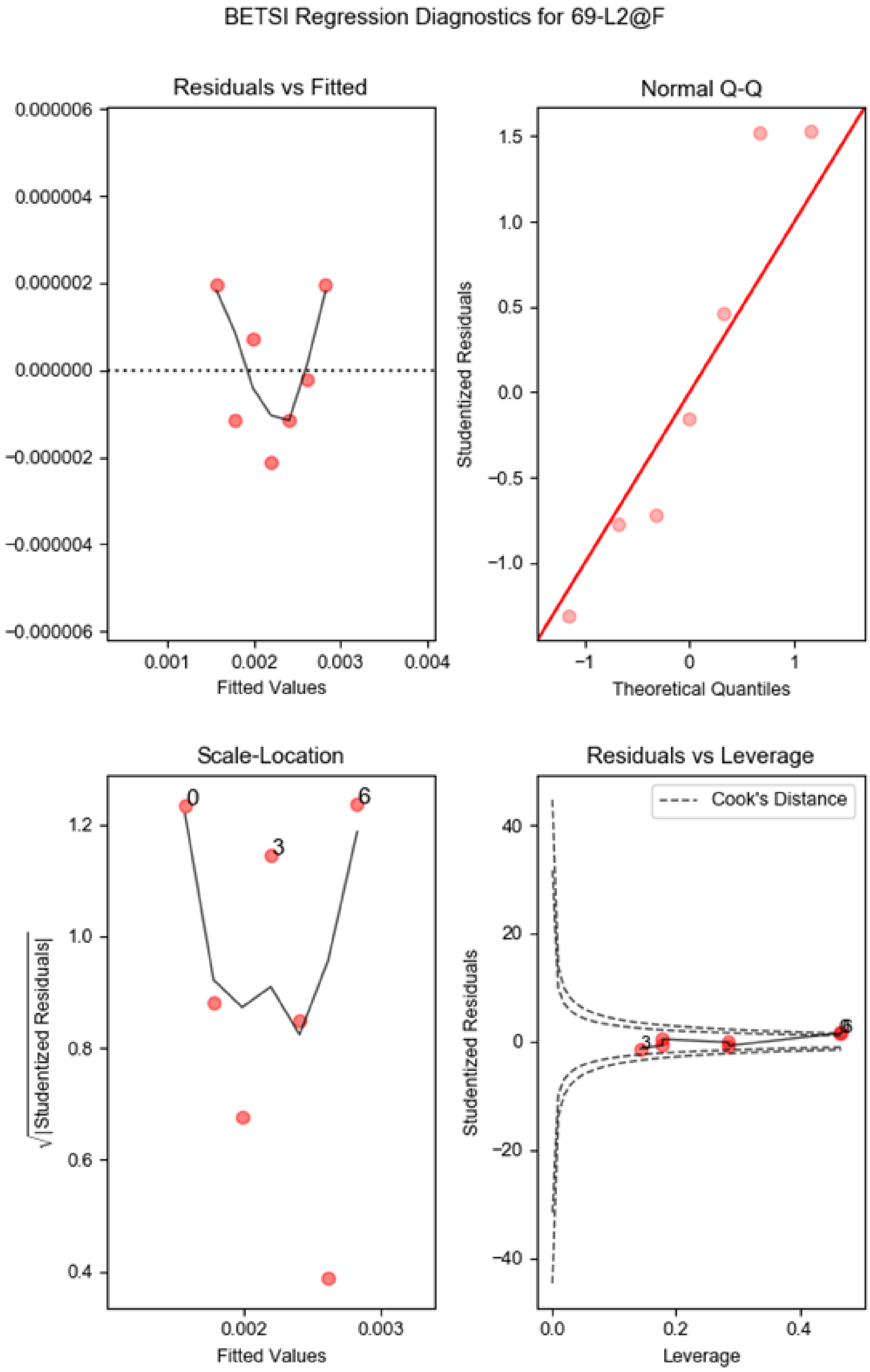
BETSI Regression Diagnostics for 69-L2@F
Residuals vs Fitted
Fitted Values
Normal Q-Q
Studentized Residuals
Theoretical Quantiles
Scale-Location
√|Studentized Residuals|
Fitted Values
Residuals vs Leverage
Cook's Distance
Studentized Residuals
Leverage

S7. Additional X-ray Crystallographic Ctructures

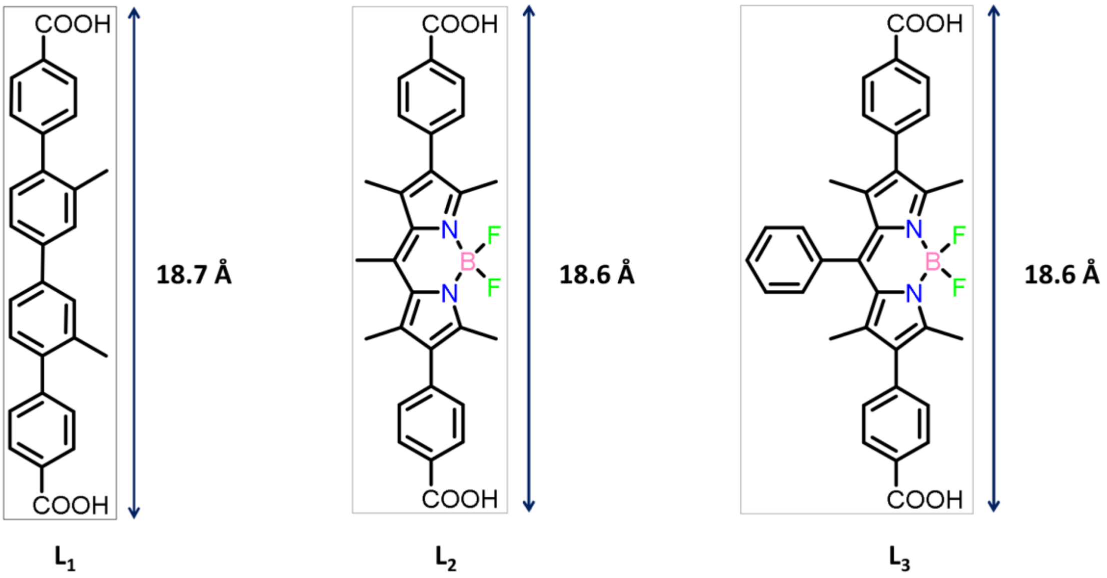

Figure S44. The lengths of L_1, L_2, and L_3.

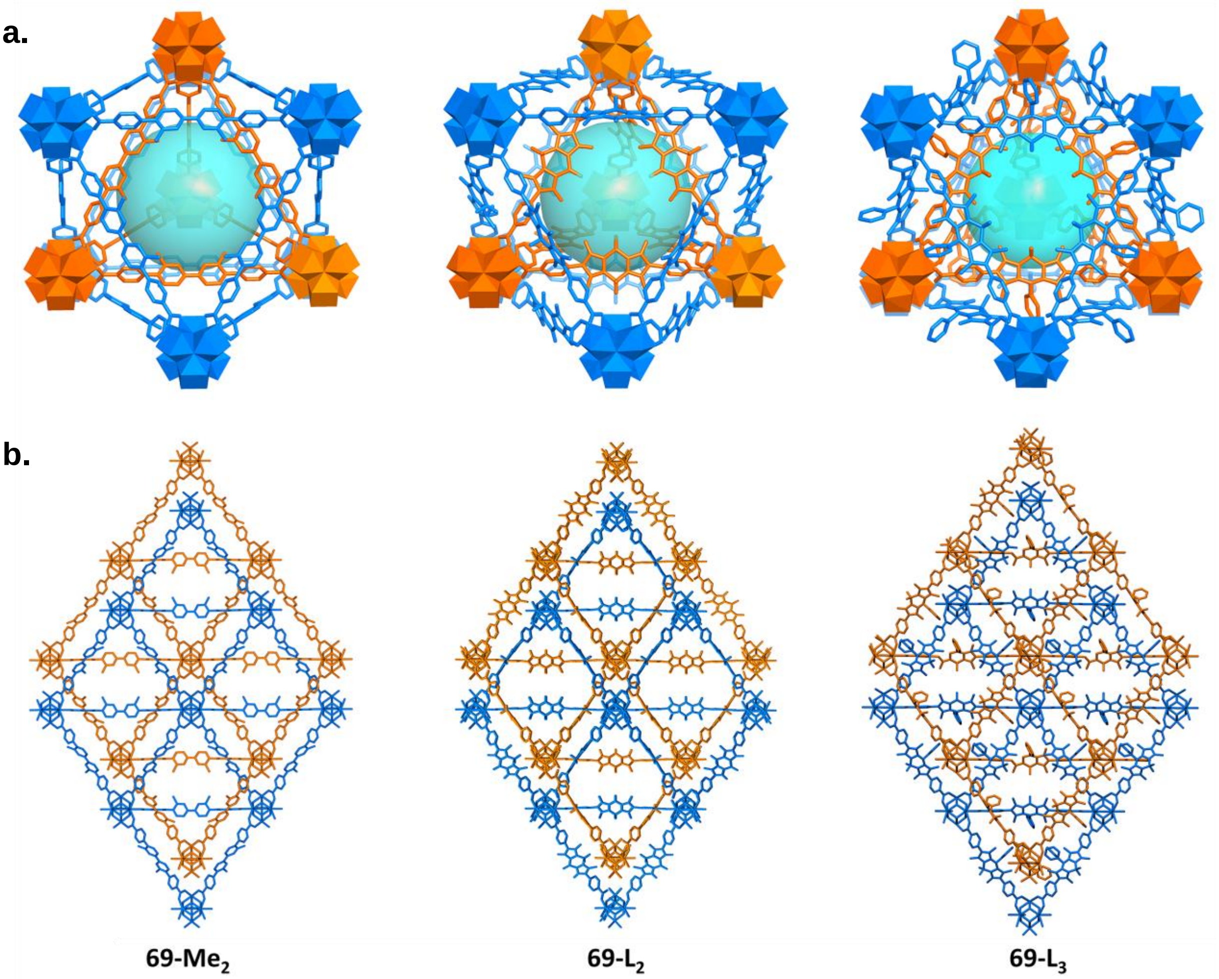

Figure S45. a. The structures of tetrahedral cavities (cyan), and **b**, two-fold interpenetrated frameworks of 69-Me_2, 69-L_2, and 69-L_3. Hydrogen atoms are omitted for clarity.

Table S1. Crystal data and structure refinement for 69-L_2 and 69-L_3

Identification code	CCDC: 2191861	CCDC: 2296146
Empirical formula	$C_{192}H_{178}B_6F_{12}N_{16}O_{36}Zr_6$	$C_{222}H_{190}B_6F_{18}N_{16}O_{36}Zr_6$
Formula weight	4125.67	4612.07
Temperature/K	100(2)	100(2)
Wavelength/Å	Synchrotron (λ = 0.72932)	Synchrotron (λ = 0.72932)
Crystal system	Cubic	Cubic
Space group	Fd-3m	Fd-3m
a/Å	38.39500(10)	38.50370(10)
b/Å	38.39500(10)	38.50370(10)
c/Å	38.39500(10)	38.50370(10)
α/°	90	90
β/°	90	90
γ/°	90	90
Volume/Å^3, *Z*	56601.0(4), 8	57083.1(4)
ρ_{calcg}/cm^3	0.968	1.073
μ/mm^{-1}	0.292	0.299
F(000)	16864.0	18832.0
2Θ range for data collection/°	5.334 to 54.18	5.318 to 54.23
Index ranges	-44<= *h* <=45<br>-47<= *k* <=47<br>-47<= *l* <=47	-48<= *h* <=48<br>-48<= *k* <=48<br>-47<= *l* <=48
Reflections collected	106801	106371
Independent reflections	2733 [R_{int} = 0.0304, R_{sigma} = 0.0081]	2762 [Rint = 0.0322, Rsigma = 0.0101]
Data/restraints/parameters	2733/287/235	2762/377/271
Goof	1.676	1.849
Final R indexes [I>=2σ (I)]	R_1 = 0.0971, *w*R_2 = 0.3332	R_1 = 0.0960, *w*R_2 = 0.3684
Final R indexes [all data]	R_1 = 0.1000, *w*R_2 = 0.3435	R_1 = 0.1002, *w*R_2 = 0.3796
Largest diff. peak/hole / e Å^{-3}	2.41/-0.80	1.78/-0.69

Table S2 Selected bond lengths [Å] and angles [º] for 69-L_2

Zr01 - O002	2.126(2)	O003 - Zr01 - O002^5	68.13(15)
Zr01 - O002^5	2.126(2)	O003 - Zr01 - O003^5	101.2(3)
Zr01 - O003	2.0870(17)	O003^5 - Zr01 - O004	145.21(18)
Zr01 - O003^5	2.0871(18)	O003 - Zr01 - O004	86.5(2)
Zr01 - O004	2.098(7)	O004 - Zr01 - O002	143.75(17)
O002^5 - Zr01 - O002	108.1(3)	O004 - Zr01 - O002^5	84.1(2)
O003^5 - Zr01 - O002^5	68.13(15)	O003^5 - Zr01 - O002	68.13(15)
O003 - Zr01 - O002	68.13(15)		

Symmetry transformations used to generate equivalent atoms:

^{1}3/4-Z,3/4-X,+Y; ^{2}+Y,3/4-Z,3/4-X; ^{3}+Y,+Z,+X; ^{4}+Z,+X,+Y; ^{5}3/4-X,3/4-Y,+Z; ^{6}+X,1/2+Z,-1/2+Y; ^{7}+X,5/4-Y,1/4-Z

Table S3 Selected bond lengths [Å] and angles [º] for 69-L_3

Zr01 - O002^5	2.156(3)	O003^5 - Zr01 - O002	70.17(17)
Zr01 - O002	2.156(3)	O003^5 - Zr01 - O003	105.0(4)
Zr01 - O003^5	2.109(2)	O003^5 - Zr01 - O004	145.8(4)
Zr01 - O003	2.109(2)	O003 - Zr01 - O004	82.5(5)
Zr01 - O004	2.124(15)	O004 - Zr01 - O002^5	80.0(3)
O002 - Zr01 - O002^5	112.3(4)	O004 - Zr01 - O002	81.8(3)
O003 - Zr01 - O002^5	70.18(17)	O003^5 - Zr01 - O002^5	70.18(17)
O003 - Zr01 - O002	70.17(17)		

Symmetry transformations used to generate equivalent atoms:

^{1}3/4-Z,3/4-X,+Y; ^{2}+Y,3/4-Z,3/4-X; ^{3}+Z,+X,+Y; ^{4}+Y,+Z,+X; ^{5}3/4-X,3/4-Y,+Z; ^{6}+X,1/2+Z,-1/2+Y; ^{7}+X,5/4-Y,1/4-Z

Table S4. ICP-OES analysis for 69-L_2@P and 69-L_2@F

Sample	P (ppm)	Zr (ppm)	PEG loading (wt%)
69-L_2@P batch 1	0.037549096	3.833998622	19.4
69-L_2@P batch 2	0.029658185	2.563730218	22.1
69-L_2@P batch 3	0.025422343	2.011467253	23.7
69-L_2@F batch 1	0.021017144	2.594717573	16.9
69-L_2@F batch 2	0.019827533	1.95927668	20.2
69-L_2@F batch 3	0.02381091	3.101977208	16.1

PEG loading = Mass of PEG loaded / (Mass of MOF + Mass of PEG loaded)

S8. References

1. Winter, G., xia2: an expert system for macromolecular crystallography data reduction. *J. Appl. Crystallogr.* **2009,** *43*, 186-190.
2. Hasse, H.; Kany, H. P.; Tintinger, R.; Maurer, G., Osmotic Virial Coefficients of Aqueous Poly(ethylene glycol) from Laser-Light Scattering and Isopiestic Measurements. *Macromolecules* **1995,** *28*, 3540-3552.
3. Wenger, R. H.; Kurtcuoglu, V.; Scholz, C. C.; Marti, H. H.; Hoogewijs, D., Frequently asked questions in hypoxia research. *Hypoxia (Auckl)* **2015,** *3*, 35-43.
4. Nepomnyashchii, A. B.; Broring, M.; Ahrens, J.; Bard, A. J., Synthesis, photophysical, electrochemical, and electrogenerated chemiluminescence studies. Multiple sequential electron transfers in BODIPY monomers, dimers, trimers, and polymer. *J. Am. Chem. Soc.* **2011,** *133*, 8633-8645.
5. Lippke, J.; Brosent, B.; von Zons, T.; Virmani, E.; Lilienthal, S.; Preusse, T.; Hulsmann, M.; Schneider, A. M.; Wuttke, S.; Behrens, P.; Godt, A., Expanding the Group of Porous Interpenetrated Zr-Organic Frameworks (PIZOFs) with Linkers of Different Lengths. *Inorg. Chem.* **2017,** *56*, 748-761.
6. Noh, H.; Kung, C.-W.; Islamoglu, T.; Peters, A. W.; Liao, Y.; Li, P.; Garibay, S. J.; Zhang, X.; DeStefano, M. R.; Hupp, J. T.; Farha, O. K., Room Temperature Synthesis of an 8-Connected Zr-Based Metal–Organic Framework for Top-Down Nanoparticle Encapsulation. *Chem. Mater.* **2018,** *30*, 2193-2197.
7. Chen, X.; Zhuang, Y.; Rampal, N.; Hewitt, R.; Divitini, G.; O'Keefe, C. A.; Liu, X.; Whitaker, D. J.; Wills, J. W.; Jugdaohsingh, R.; Powell, J. J.; Yu, H.; Grey, C. P.; Scherman, O. A.; Fairen-Jimenez, D., Formulation of Metal-Organic Framework-Based Drug Carriers by Controlled Coordination of Methoxy PEG Phosphate: Boosting Colloidal Stability and Redispersibility. *J. Am. Chem. Soc.* **2021,** *143*, 13557-13572.
8. Wu, W.; Guo, H.; Wu, W.; Ji, S.; Zhao, J., Organic triplet sensitizer library derived from a single chromophore (BODIPY) with long-lived triplet excited state for triplet-triplet annihilation based upconversion. *J. Org. Chem.* **2011,** *76*, 7056-7064.
9. Keiper, J. S.; Simhan, R.; DeSimone, J. M.; Wignall, G. D.; Melnichenko, Y. B.; Frielinghaus, H., New phosphate fluorosurfactants for carbon dioxide. *J. Am. Chem. Soc.* **2002,** *124*, 1834-1835.
10. Zhao, G.; Tong, L.; Cao, P.; Nitz, M.; Winnik, M. A., Functional PEG-PAMAM-tetraphosphonate capped $NaLnF_4$ nanoparticles and their colloidal stability in phosphate buffer. *Langmuir* **2014,** *30*, 6980-6989.
11. Uemura, T.; Yanai, N.; Watanabe, S.; Tanaka, H.; Numaguchi, R.; Miyahara, M. T.; Ohta, Y.; Nagaoka, M.; Kitagawa, S., Unveiling thermal transitions of polymers in subnanometre pores. *Nat. Commun.* **2010,** *1*, 83.
12. Xie, Z.; Wang, B.; Yang, Z.; Yang, X.; Yu, X.; Xing, G.; Zhang, Y.; Chen, L., Stable 2D Heteroporous Covalent Organic Frameworks for Efficient Ionic Conduction. *Angew. Chem. Int. Ed.* **2019,** *58*, 15742-15746.
13. Osterrieth, J. W. M.; Rampersad, J.; Madden, D.; Rampal, N.; Skoric, L.; Connolly, B.; Allendorf, M. D.; Stavila, V.; Snider, J. L.; Ameloot, R.; Marreiros, J.; Ania, C.; Azevedo, D.; Vilarrasa-Garcia, E.; Santos, B. F.; Bu, X. H.; Chang, Z.; Bunzen, H.; Champness, N. R.; Griffin, S. L.; Chen, B.; Lin, R. B.; Coasne, B.; Cohen, S.; Moreton, J. C.; Colon, Y. J.; Chen, L.; Clowes, R.; Coudert, F. X.; Cui, Y.; Hou, B.; D'Alessandro, D. M.; Doheny, P. W.; Dinca, M.; Sun, C.; Doonan, C.; Huxley, M. T.; Evans, J. D.; Falcaro, P.; Ricco, R.; Farha, O.; Idrees, K. B.; Islamoglu, T.; Feng, P.; Yang, H.; Forgan, R. S.; Bara, D.; Furukawa, S.; Sanchez, E.; Gascon, J.; Telalovic, S.; Ghosh, S. K.; Mukherjee, S.; Hill, M. R.; Sadiq, M. M.; Horcajada, P.; Salcedo-Abraira, P.; Kaneko, K.; Kukobat, R.; Kenvin, J.; Keskin, S.; Kitagawa, S.; Otake, K. I.; Lively, R. P.; DeWitt, S. J. A.; Llewellyn, P.; Lotsch, B. V.; Emmerling, S. T.; Putz, A. M.; Marti-Gastaldo, C.; Padial, N. M.; Garcia-Martinez, J.; Linares, N.; Maspoch, D.; Suarez Del Pino, J. A.; Moghadam, P.; Oktavian, R.; Morris, R. E.; Wheatley, P. S.; Navarro, J.; Petit, C.; Danaci, D.; Rosseinsky, M. J.; Katsoulidis, A. P.; Schroder, M.; Han, X.; Yang, S.; Serre, C.; Mouchaham, G.; Sholl, D. S.; Thyagarajan, R.; Siderius, D.; Snurr, R. Q.; Goncalves, R. B.; Telfer, S.; Lee, S. J.; Ting, V. P.; Rowlandson, J. L.; Uemura, T.; Iiyuka, T.; van der Veen, M. A.; Rega, D.; Van Speybroeck, V.; Rogge, S. M. J.; Lamaire, A.; Walton, K. S.; Bingel, L. W.; Wuttke, S.; Andreo, J.; Yaghi, O.; Zhang, B.; Yavuz, C. T.; Nguyen, T. S.; Zamora, F.; Montoro, C.; Zhou, H.; Kirchon,

A.; Fairen-Jimenez, D., How Reproducible are Surface Areas Calculated from the BET Equation? *Adv. Mater.* **2022**, e2201502.
